# Multilaboratory Untargeted Mass Spectrometry Metabolomics Collaboration to Identify Bottlenecks and Comprehensively Annotate A Single Dataset

**DOI:** 10.1021/acs.analchem.4c05577

**Published:** 2025-07-22

**Authors:** Joelle Houriet, Preston K. Manwill, Armando Alcázar Magaña, Victoria M. Anderson, Mehdi A. Beniddir, Samuel Bertrand, Jaewoo Choi, Trevor N. Clark, Leonard J. Foster, Maria Halabalaki, Alan K. Jarmusch, Niek F. de Jonge, Aswad Khadilkar, John B. MacMillan, Claudia S. Maier, Luke C. Marney, Guillaume Marti, Eleni V. Mikropoulou, Damien Olivier-Jimenez, Amélie Perez, Justin J. J. van der Hooft, Mitja M. Zdouc, Roger G. Linington, Nadja B. Cech

**Affiliations:** † Department of Chemistry & Biochemistry, 14616 University of North Carolina at Greensboro, Greensboro, North Carolina 27402, United States; ‡ Life Sciences Institute, Department of Biochemistry and Molecular Biology, 8166 University of British Columbia, Vancouver, BC V6T 1Z4, Canada; § Équipe “Chimie des substances naturelles” BioCIS, CNRS, Université Paris-Saclay, 17, avenue des Sciences, 91400 Orsay, France; ∥ Nantes Université, Institut des Substances et Organismes de la Mer, ISOMER, UR 2160, 44000 Nantes, France; ⊥ Nantes Université, École Centrale Nantes, CNRS, LS2N, UMR 6004, 44000 Nantes, France; # Department of Chemistry, 2694 Oregon State University, Corvallis, Oregon 97331, United States; ∇ Department of Chemistry, 1763 Simon Fraser University, Burnaby, BC V5A 1S6, Canada; ○ Division of Pharmacognosy and Natural Products Chemistry, Department of Pharmacy, 68993 National and Kapodistrian University of Athens, 157 71 Zographou, Greece; ◆ Immunity, Inflammation, and Disease Laboratory, Division of Intramural Research, National Institute of Environmental Health Sciences, National Institutes of Health, Research Triangle Park, North Carolina 27709, United States; ¶ Bioinformatics Group, 4508 Wageningen University & Research, Wageningen 6708 PB, The Netherlands; ⋈ Department of Chemistry & Biochemistry, 8787 University of California Santa Cruz, Santa Cruz, California 95064, United States; ⧓ Laboratoire de Recherche en Sciences Végétales, Metatoul-AgromiX Platform, Université de Toulouse, CNRS, INP, 31320 Auzeville-Tolosane, France; ⧖ MetaboHUB-MetaToul, National Infrastructure of Metabolomics and Fluxomics, Toulouse 31400, France; ● Center for Proteomics and Metabolomics, Leiden University Medical Center, 2333 ZA Leiden, The Netherlands; ¤ Department of Biochemistry, University of Johannesburg, Johannesburg 2006, South Africa

## Abstract

Annotation is the process of assigning features in mass spectrometry metabolomics data sets to putative chemical structures or “analytes.” The purpose of this study was to identify challenges in the annotation of untargeted mass spectrometry metabolomics datasets and suggest strategies to overcome them. Toward this goal, we analyzed an extract of the plant ashwagandha (*Withania somnifera*) using liquid chromatography–mass spectrometry on two different platforms (an Orbitrap and Q-ToF) with various acquisition modes. The resulting 12 datasets were shared with ten teams that had established expertise in metabolomics data interpretation. Each team annotated at least one positive ion dataset using their own approaches. Eight teams selected the positive ion mode data-dependent acquisition (DDA) data collected on the Orbitrap platform, so the results reported for that dataset were chosen for an in-depth comparison. We compiled and cross-checked the annotations of this dataset from each laboratory to arrive at a “consensus annotation,” which included 142 putative analytes, of which 13 were confirmed by comparison with standards. Each team only reported a subset (24 to 57%) of the analytes in the consensus list. Correct assignment of ion species (clusters and fragments) in MS spectra was a major bottleneck. In many cases, in-source redundant features were mistakenly considered to be independent analytes, causing annotation errors and resulting in overestimation of sample complexity. Our results suggest that better tools/approaches are needed to effectively assign feature identity, group related mass features, and query published spectral and taxonomic data when assigning putative analyte structures.

## Graphical abstract

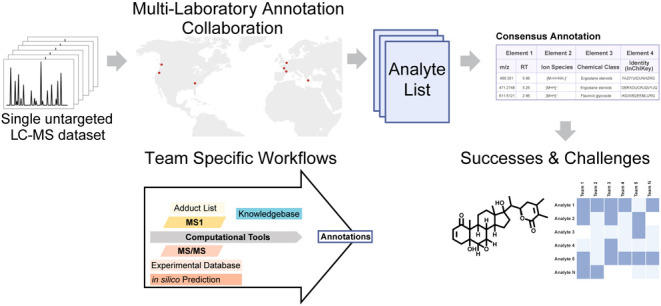

Untargeted mass spectrometry (MS) metabolomics is often employed to generate hypotheses and measure differences across biological samples.^1^ Rapid identification of known analytes in these samples remains a bottleneck that many teams are trying to address.^2–4^ To annotate (putatively identify) analytes detected in MS data sets, analysts rely on mass-to-charge ratio (*m*/*z*), isotope patterns of the detected ions, fragmentation patterns observed in MS/MS spectra, and chromatographic retention times (see Supporting Information file 01 (SI-01), Table S1 for the terminology and definition of key terms). Annotations derived from liquid chromatography – mass spectrometry (LC-MS) data are referred to as “tentative” or “putative” because a full three-dimensional structure cannot be assigned without orthogonal techniques such as NMR or X-ray crystallography. Annotation confidence can, however, be improved by comparison of retention time, mass, and fragmentation patterns with authentic standards,^5^ by interpreting isotope distributions,^6^ and, when ion mobility measurements are available, with collisional cross section (CCS) data.^7^ Such approaches are valuable because they can be accomplished rapidly and with analytes present in mixtures at levels too low to enable isolation.^8^

Numerous tools exist for annotation based on LC-MS data.^4^ Many of these rely on fragmentation spectra (MS/MS) to match spectral data with structures. Some of these approaches, such as the global natural product social molecular networking platform (GNPS)^9^ and Massbank,^10^ match measured fragmentation spectra with pre-existing databases, while other tools such as SIRIUS,^11^ CFM-ID,^12,13^ and MS-Finder^14^ rely on predicted fingerprints and/or fragmentation spectra. Annotation confidence can be improved by searching against existing resources (knowledgebases) such as KEGG,^15^ HMDB,^16^ the Natural Product Atlas,^17^ and LOTUS^18^ to determine whether the proposed analyte has previously been reported in the same or similar biological source.^19^

The “certainty” associated with a given annotation depends on how the structure was assigned. To capture differences in annotation confidence, some annotation tools provide quantitative scores based on spectral similarity, which may also be combined with other parameters.^19^ The “confidence levels,” first proposed 16 years ago^5^ and revisited again more recently,^20–22^ are another way to express the level of certainty for annotation.

Despite efforts to standardize approaches for annotating metabolomics data sets, heterogeneity remains in how mass spectrometry metabolomics data are processed and interpreted. Effective annotation is hampered by data set complexity and the presence of chemical interferences that are difficult to remove. Various preprocessing steps are employed for interpretation, which include “peak picking” or the detection of features, alignment, gap-filling, and filtration (or cleaning) of the data set (SI-01 Table S1). Several pipelines for data preprocessing exist, including open-source packages such as MZmine,^23^ MS-DIAL,^24^ XCMS,^25^ and vendor-specific packages such as Progenesis QI (Waters), Compound Discoverer (Thermo Fisher Scientific), and MetaboScape (Bruker).

Analyte annotation in metabolomics is further complicated by redundant features (degenerate features^26^) formed in the electrospray ionization (ESI) process.^22,27^ These include clusters (such as potassium, sodium, or ammonium adducts), fragments (formed, for example, by loss of water or cleavage of glycosidic bonds), or multimers of the analyte, such as proton-bound dimers. Several tools have been designed to group the features originating from the same analyte and annotate them, based on either peak shape and/or mass differences. These include CAMERA,^28^ RAMClust,^29^ BINNER,^26^ MS-CleanR,^30^ and ion identity networking (IIN).^31^

Data processing steps have already been shown to impact metabolomics data interpretation, and guidelines have been proposed to improve data set quality.^22^ Differences in data preprocessing can impact the quality of data sets and the conclusions drawn from them.^32–34^ The effectiveness of annotation tools has so far been compared in several challenges, where participants were provided with preselected spectra to annotate.^35,36^

With this study, we also employed a strategy of having multiple teams analyze the same data set. Our study is, however, unique in that we were interested in comparing the annotations of a complex metabolomics data set proposed by several teams using a truly untargeted design. Raw data from LC-MS metabolomics analyses conducted in 12 different acquisition modes (Orbitrap and Q-ToF data) were provided to multiple participants, and they were instructed to select a data set and annotate it using their own preferred workflows. Participants were told the species of the plant sample from which the extract was prepared, but they were given no reference standards or information in advance about the nature of the analytes present in the sample. The number of analytes present was completely unknown to everyone, including the two teams that initiated the analysis. Rather than choosing human biological samples for which ample data exist that enable targeted analysis, we chose an extract from the plant ashwagandha [*Withania somnifera* (L.) Dunal], a sample known to be rich in isomers.^37^ Each laboratory analyzed the LC-MS metabolomics data from this extract using whichever tools and pipelines they preferred and reported a list of analytes present in the sample, along with a reported confidence level. Our goal with this study was to assess how similar the results would be across teams and to generate an improved “consensus annotation” relying on the data that each team provided.

## Experimental Section

### Botanical Extraction

The roots of *Withania somnifera* (L.) Dunal (Solanaceae) were obtained as a Botanical Reference Material from the American Herbal Pharmacopoeia (Scotts Valley, CA). The roots were extracted in triplicate by combining ca. 20 mL of reagent-grade methanol with ca. 200 mg of botanical product (1:10 dilution). The mixtures were shaken for 24 h at room temperature, filtered, concentrated to dryness under nitrogen gas, and stored at −20 °C. The dried samples were reconstituted in 10 mL of methanol, aliquoted (100 μg) into LC-MS vials, dried under nitrogen, and stored at −20 °C. Process blanks (labeled as Extraction blanks) were prepared in parallel by subjecting the extraction solvents to the identical series of steps used to extract the plant material, but in the absence of plant material.

### Sample Preparation

The dried samples were reconstituted to 100 μg/mL in 1:1 Optima grade methanol and Optima grade water, sonicated, vortexed, and stored at −80 °C prior to analysis. The set of reference mixtures was prepared in equimolar concentrations of each compound at 10 and 100 μM in two solutions. Aliquots of the extract used in this study are available upon request.

### Chromatographic Conditions

Separation was performed with an Acquity HSS T3 C18 column of 1.8 μm (2.1 mm × 100 mm). Mobile phase A was 100% CH_3_CN, and mobile phase B was 100% H_2_O, both containing 0.01% HCOOH. Gradient elution mode was as follows: 0–0.3 min, 5% A; 0.3–9.1 min, 5 to 90% A; 9.1–10.7 min, 90 to 98% A; 10.7–11.0 min, 98% A; 11.01–12.8 min, 5% A. 0.5 mL/min, column temperature 40 °C, injection volume 5 μL.

### LC-MS Data Acquisition

Data were collected with an Acquity UPLC I-Class coupled to either (1) a Waters SYNAPT G2-Si (Q-TOF) or (2) a Thermo Q-Exactive Plus (Orbitrap). Triplicate analyses were conducted in full scan, data-dependent acquisition (DDA), and data-independent acquisition (DIA) modes using both positive and negative ionization. Details on LC-MS data acquisition and preprocessing are available in SI-01 Section 2.

### Instructions for the Collaboration

Participants downloaded raw or converted data from the *Withania somnifera* extract, process blanks, and solvent blanks. They had no access to commercial standard data. They were asked to select and annotate at least one positive ionization data set (from among the six different positive ion data sets provided) (SI-02). The inclusion of the analysis of a negative ion mode data set was optional. In analyzing the selected data set, participants were asked to provide annotation data for analytes, not an exhaustive list of detected features. Participants were free to provide a single feature per analyte or multiple features. The participants ranked their annotations on a confidence level scale (SI-01 Tables S2–S3) adapted from.^5,20–22^

Details of the strategies applied by each team are available in SI-01 Section 4 and Figure S1–S8.

### Annotation Table Harmonization

Annotation tables from all participants were loaded in Excel 16 with ChemDraw v.21.0 add-ins (PerkinElmer Informatics, Inc.). Participants were asked to provide the annotation InChIs and InChIKeys, and their chemical classes according to ClassyFire^38^ or Natural Product Classifier (NP Classifier).^39^ Where possible, missing InChIs and/or InChIKeys were inferred from the common name or descriptor provided by consulting PubChem and LOTUS^18^ and/or generated by the APIs provided by GNPS.^40^ Next, the InChIs were converted to SMILES using the appropriate GNPS API through the Excel formula “webservice”. The SMILES was then employed to generate the chemical classes defined by the NP Classifier API to obtain homogenized chemical classes. LogPs were calculated from the SMILES using SwissADME (consensus LogP).^41^ The ion species descriptions were standardized using the notation defined in Kachman et al.:^26^ [M+ charge carrier ± neutral gain(s)/loss­(es)]^+^.

### Annotation Comparison

A total of ten teams were involved in the collaboration. Only two teams chose to analyze the Q-ToF data, so the Q-ToF results were omitted from further analysis. The remaining eight teams chose to annotate the Orbitrap data collected in positive ion data-dependent acquisition (DDA) mode; therefore, these data were chosen as the focus of the study. The results reported for each laboratory using this dataset were merged into a single table, named “Merged Annotation Table” (SI-03 Table S1), with 799 features. Rows containing multiple ion species were split so that only one feature was represented in each row. Features reported by more than one laboratory are displayed in individual rows with their respective proposed annotations. Annotations at the solvent front (before 0.8 min) and in the washout time (after 9 min) that are particularly difficult to confirm were excluded from the “Merged Annotation Table” (SI-03 Table S1) and are not included in the total of 799 features.

### Annotation Agreement Scores

The Merged Annotation Table was checked for duplicated entries (same reporting team, retention times, *m*/*z*, areas, and identities), and 31 duplicates were rejected, leaving a total of 768 features. For these remaining features, four “Elements” of the annotations were compared to assess agreement across teams: (1) “feature reported” (the number of teams that reported a given features with the same RT (±0.05 min) and *m*/*z* (±0.003)), (2) “ion species description” (the number of teams that assigned a particular ion species, [M+X]^+^, to a given feature) (3) “chemical class” (the number of teams that assigned a particular chemical class, as defined by NP Classifier,^39^ to a given feature) and (4) “identity” (the number of teams that assigned a particular 2-dimensional structure to a given feature) (SI-01 Table S1).

*Annotation Agreement Scores* (eq 1) were calculated to numerically compare the annotations across the 8 laboratories. To calculate the *Annotation Agreement Scores,* an *Assignment Match* (*j*) was first obtained by counting the number of teams that agreed (gave the same assignment) for a given Element (*i*) of a given annotation. For example, an *Assignment Match* of 3 for Element 1 (feature reported) would indicate that three teams reported the same feature. The *Occurrence* (*A*_*j*,*i*_) was then obtained by counting the number of times a given *Assignment Match* (*j*) was obtained for each Element (*i*). Finally, the *Annotation Agreement Score* was calculated as the ratio of *Occurrence* (*A*_*j,i*_) to *Assignment Match* (*j*) *(* eq 1 *)*. The purpose of taking the ratio was to avoid duplication in cases where features were reported by more than one team.

1 $$\mathrm{Annotation}\;\mathrm{Agreement}\;\mathrm{Score}=\frac{A_{j,i}}{j}$$

Qualitatively, the *Annotation Agreement Score* is a measure of how many teams agreed on the assignment of a given element for a given feature. For an in-depth explanation with examples for calculating the *Annotation Agreement Score*, please see Figure S18 (SI-01).

### Consensus Annotations

The 768 features from the Merged Annotation Table that remained after duplicate removal were checked against various criteria to keep only high-quality features (see definition in SI-01 Table S1, data preprocessing details in SI-01 Section 2 and Figure S20A). Features were rejected if they (1) were not detected in the peak-picking strategy used by the organizing team (SI-01 Section 2) (18 features (2.3%)), (2) were detected in the blanks (46 features (5.8%)), (3) had a relative standard deviation in *W. somnifera* extract triplicate analyses >30% (16 features (2.0%)), (4) did not have an MS/MS spectrum (71 features (8.9%)) (5) did not have at least two isotopes (7 features, 0.9%) (Figure S23A). Discarding features without MS/MS spectra reduced the labor of data interpretation, but these could represent analytes below the MS/MS threshold or analytes that coelute with higher abundance features.

The annotations were cross-compared across teams, relying on the collective data and published literature to decide which assignments were most probable. The structures of withanolides were assigned based on previously published fragmentation patterns.^42,43^

When the proposed annotations had been described in phyla other than *W. somnifera* (Streptophyta), searches were conducted to propose a more plausible annotation. These searches involved analyzing MS/MS data with SIRIUS 5.6.3 software or finding more taxonomically reasonable alternatives. The taxonomy was retrieved from LOTUS,^18^ KNApSAcK,^44^ HMDB,^45^ from a recent review on *Withania* genus,^46^ or from relevant publications.^47–49^ To assign the ion species, a published^50^ list of putative fragments or complexes was employed (SI-04). Ion species for features were assigned based on observed mass differences (SI-04) and, where available, by comparing data with standards.

As a final step, 18 standards were analyzed to check some of the most common annotations. The assignments of feature identities after all annotation steps are summarized in the Consensus Annotation Table (SI-03, Tables S2 and S3). As descriptors, the InChiKeys, InChIs, and 2D SMILES and a common name (if available) are provided. Reference is made to international identifiers (LOTUS identifier and, if the molecule was absent in LOTUS, PubChem identifier). For 3-dimensional isomers, only one international identifier is provided, as its 2D SMILES can lead to the 3-dimensional isomers. All possible isomers are referenced if the annotation leads to several possible structures. No annotation could be proposed for 15 analytes (unresolved), and two analytes were annotated up to the chemical classes with CANOPUS^51^ but were not assigned a specific identity.

We limited the annotation to features that were reported by at least one participant. While more features could have been found and annotated, this was beyond the scope of this project. Figure S38A in SI-01 illustrates the features that were annotated in the Consensus Annotation Table in the LC-MS metabolite profiling, while Figure S38B shows the numbers of features retained after our filtering strategies but not annotated in the project.

### Confidence Level of the Consensus Annotations

For the features that could not be confirmed with standards, confidence levels were assigned in two ways: if the final annotation matched annotations made by some participants, the best level indicated by the participant(s) was retained, given that the participants could, for example, have access to *in-house* or commercial experimental databases that justified a level 2. If the annotation was an in-source feature, a level of 5 was assigned. Finally, if the annotations were refined through fragmentation prediction, a level of 3 was assigned (SI-01 Table S2).

### Consensus Annotation Scores

A second scoring system was established to measure the extent to which the annotations reported by each team agreed with the consensus annotations (SI-01 Figure S21). The same four annotation elements used in the *Annotation Agreement Score* calculations were considered: reported feature, ion species, chemical class, and identity, and a score of 1 was assigned if the proposed annotation agreed with the consensus annotation. The sums for each team and each element were then assigned at the feature level (SI-01 Figure S22) and the analyte level (Figure 3). For the analytes with more than one feature, the sum of the scores for each feature for each laboratory was first calculated for the corresponding analyte, and then the scores were reduced to 1 to consider only the analyte.

## Results and Discussion

Ten teams participated, including two teams that initiated the project. Five teams were from Europe and five from North America (SI-01 Section 4). Three of the ten teams had previously worked on *W. somnifera*, eight teams considered themselves end-users, and two considered themselves both end-users and developers. Eight participants selected the dataset acquired on the Orbitrap in positive ionization DDA mode, and only two teams selected the data set obtained from the Q-TOF in positive ionization DIA mode. Only two teams analyzed the negative mode data sets. The results presented here are thus limited to the datasets acquired on the Orbitrap in positive ionization mode.

### Interlaboratory Comparison of Data Preprocessing and Annotation Methods

All teams relied on automatic peak-picking using either open-source or vendor-specific tools (SI-01 Figure S9). A range of signal intensity thresholds was selected (SI-01 Figure S10). Teams applied several strategies to filter and reduce the number of features (SI-01 Figure S11), with the most popular being isotopic feature grouping (i.e., removing isotopes from the features lists) and blank filtering. For the removal of features in the blanks, three teams applied a qualitative strategy, *e.g*. removing the features detected in a defined number of blanks and two used a quantitative strategy similar to that published previously,^30^ in which the ratio in peak areas of each feature was compared between the sample and blank to determine if a feature should be retained.

The annotation strategies employed ranged from almost fully automatic to manual methods (SI-01 Figures S1–S8). Some of the annotation strategies were already published, some were under review, and some were developed *in-house*. The teams relied on MS data to determine molecular formulas, and features in the MS data were annotated based on specific *in-house* lists of adducts, neutral losses, or gains. There was variability in the *in-house* lists of adducts, neutral losses or gains used by each team, with some using as few as four species and others several hundred (SI-01 Figures S1–S8, and SI-04, Table S2).

Participants were asked to provide annotation data for analytes, not an exhaustive list of detected features, and they were free to report redundant features or not. Reporting analytes implied that the teams had to group or delete redundant features associated with a given analyte. To group redundant features, four teams employed automatic approaches. One used CAMERA,^28^ others used MS-CleanR,^26^ MolNotator,^52^ or the *in-house* prototype software MS2Analyte.

All teams used MS/MS data for the annotation. Among these, six teams also used tools to search *in silico* annotation such as SIRIUS,^11^ CFM-ID,^12^ MS-Finder,^14^ and MS2Query^53^ (SI-01 Section 4 and Figures S1–S8). Finally, multiple knowledge bases were consulted to find analytes with relevant taxonomy (SI-01 Figures S1–S8). The teams differed in whether they considered data at the species, genus, or order level. The teams spent various amounts of time on the project (SI-01 Figure S12): from 1 to 60 h for preprocessing and from 2 to 84 h for annotation. The total time varied from 3 to 144 h. Collectively, the ten teams spent 445 h on the project, of which 312 h (70%) were spent on manual tasks.

### Global Comparison of Annotation

The number of reported analytes was similar across the ten teams, ranging from 58 to 149 (SI-01 Figure S13). However, the raw numbers of features detected differed considerably from one team to another (Figure S13A), even after filtering and reduction (Figure S13B). The reported confidence levels also differed. The teams mainly used annotation tools based on fragmentation spectra, but the extent and method in which they were employed differed. Because data for standards was not provided to the teams alongside the data from the extract, the highest reported annotation accuracy possible (Table S2) was a level 2 annotation (probable structure: Unambiguous matching literature or library MS/MS spectrum). According to Figure S15, the number of analytes reported with level 2 confidence varied greatly across the teams, with some teams (1 and 8) reporting zero analytes with level 2 confidence, and, at the other extreme, several teams (3 and 4) reporting approximately 80 analytes with level 2 confidence (SI-01 Figure S15). Reported confidence levels differed depending on which types of fragmentation spectral databases the teams consulted (experimental or predicted, e.g., confidence levels 2 to 4) and whether or not they considered taxonomic information (SI-01 Tables S2 and S3). When it came to assigning identities to the features, some chemical classes (such as ergostane steroids (“ergostane steroid” refers to “withanolide” in NP classifier class terminology), ergostane steroid glycosides, flavonol glycosides, and amino acids) were reported by all teams (SI-01 Figure S16). Only one team annotated several features as tigliane diterpenoids (Figure S16F).

### Interlaboratory Comparison of Analyte Annotation

The annotation tables reported by each team were merged into a single table which included 799 rows (referred to hereafter as “Merged Annotation Table”, SI-03 Table S1).

This table was first evaluated to assess agreement among the teams regarding the analyte identity. A total of 385 analytes (defined as unique identities reported in a 0.05 min window) were reported (SI-01 Figure S17). Of these, 322 analytes were reported by only one of the eight teams, while 32 analyte identities were reported by just two teams. The remaining analyte identities were reported by three or more teams, for a total of nine analyte identities by three teams, ten analyte identities by four teams, nine analyte identities by five teams, one analyte identity by six teams, and two analyte identities by seven teams. No analytes were reported by all eight teams.

### Interlaboratory Comparison of Feature Annotation

To understand the poor agreement among teams as to the identities of the analytes (SI-01 Figure S17), the annotations of the features reported by the 8 teams were compared in detail. *Annotation Agreement Scores* (eq 1) were calculated to compare the annotations assigned to the features in the Merged Annotation Table (SI-03 Table S1) by the 8 teams (Figure 1). The *Annotation Agreement Scores* (bold numbers in Figure 1) represent the number of teams that agreed on the various annotation elements for a given feature. For example, 246 features had an *Annotation Agreement Score* of 1 for the “feature reported” element (upper left box Figure 1), meaning that 246 features were reported by only one of the 8 teams. Moving one cell to the right, 76 features were given an *Annotation Agreement Score* of 2, meaning that 76 features were reported by two teams. As another example, 337 features were assigned an *Annotation Agreement Score* of 1 for the “ion species” element (second box down on the first column in Figure 1), meaning that there were 337 features for which a given assigned ion description (*i.e*., [M + H]^+^) was reported for only 1 of the 8 participating teams. Overall, the *Annotation Agreement Score* analysis shows that there was low consensus about which features were present in the dataset and which ion species, chemical class, and identity should be assigned to those features. The level of agreement was poorer for elements that represented tasks of higher complexity (i.e., assigning identity) than for those elements representing simpler tasks (*i.e*., reporting the presence of a feature). No feature achieved an annotation agreement score of 8, which would have indicated agreement by all 8 teams. However, the number of teams reporting the same features would likely have been higher if we had asked the teams to report on all features in the datasets, rather than allowing them to select one or more features for each annotated analyte.

**Figure 1:**
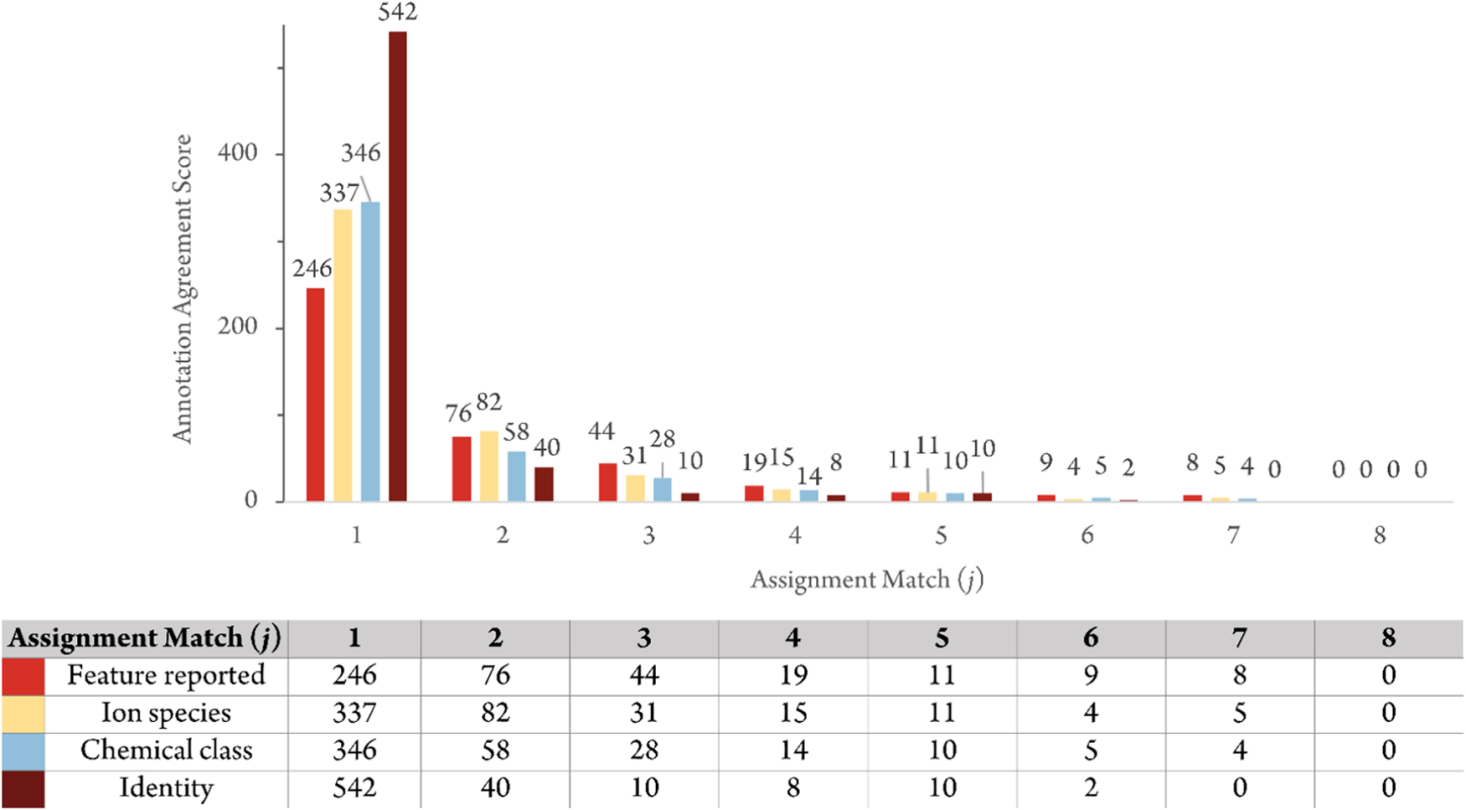
*Annotation Agreement Scores* assess the feature-level agreement between the 8 teams for the Orbitrap data-dependent analysis (DDA) positive ionization dataset. *Assignment Match* (j) is defined as the number of teams that agreed on the assignment for a given *Element*. Four *Elements* of the annotations were considered: the “feature reported” element (defined by *m*/*z* and retention time), the “ion species description” element (ion species, [M + H]^+^, [M + Na]^+^, etc.), the “chemical class” element (defined by the NP classifier), and the “identity” element (two-dimensional identity). The *Annotation Agreement Score* is calculated from eq 1 and described in more detail in SI-01 Figure S18. As an example, for *Assignment Match* 2 and the “Chemical class” element, an *Annotation Agreement Score* of 58 indicates that 58 features were assigned to the same chemical class by two teams.

Among these features reported by 7 teams, the one that accumulated the highest scores of the three other elements was described by 7 teams as an [M + H]^+^ feature belonging to the flavanol glycosides chemical class. Six teams agreed on its identity (rutin InChIKey = IKGXIBQEEMLURG). The second-highest scores were for a feature described by 7 teams as an [M + H]^+^ feature of an ergostane steroid. Five teams agreed on the identity of this feature (withaferin A (InChIKey = DBRXOUCRJQVYJQ)).

### Consensus Annotation

One of the goals of this study was to produce annotated lists of high-quality features and analytes detected in the *W. somnifera* extract (see definitions of “high-quality feature” and “analyte” in SI-01 Table S1, and annotation lists in SI-03 (features in Table S2 and analytes in Table S3)). Toward this goal, we first applied detection and filtration criteria to remove some of the features in the Merged Annotation Table (SI-01 Section 2 and Figures S20 and S23). Next, a manual evaluation of the annotations was performed to reach consensus annotations (SI-03 Table S2). A “Consensus Annotation Score” was established to evaluate the extent to which the results reported by each individual team agreed with the final consensus annotations. Finally, we removed all of the redundant reports of a given feature by multiple laboratories so that each feature was represented once as an analyte (SI-03 Table S3).

### Filtering the Consensus Features

Starting from the Merged Annotation Table, the 799 reported features were checked as described in SI-01 Figure S23. A total of 189 features were rejected (23.7%). Among the rejected features, 137 were reported by only a single team (Figure S23B). The number of rejected features that had been reported by more than one team was very low. Thus, features reported by at least two teams were more reliable, demonstrating the value of the collaboration.

### Annotation of the Consensus Features

To arrive at the “best” designation of ion species, chemical class, and identity for each feature, the filtered feature list (610 high-quality features) was manually interpreted, as described in the Experimental Section. To improve the annotation confidence, we analyzed several commercial standards using the same method employed for the *W. somnifera* extract. Standards chosen were either reported in *W. somnifera* or chosen to confirm commonly reported annotations (SI-01 Figures S36–S37). Comparison of retention time and fragmentation patterns with standards confirmed 44 features and 13 analyte annotations with a confidence level of 1 (SI-01 Tables S2–S3, SI-03 Table S2) and enabled the rejection of 51 annotations. Participants were not given retention time data for standards prior to conducting their annotations; therefore, it is expected that some of the proposed annotations would be rejected once this information is available.

As a result of these analysis steps, two tables were compiled, a “Consensus Feature Table” (SI-03 Table S2) that contained 264 high-quality features and a “Consensus Analyte Table” (SI-03 Table S3) that contained 142 *W. somnifera* analytes associated with these features.

### Reassignment of Feature Descriptions

Meticulous analysis of standard features at the MS level demonstrated the importance of in-source phenomena, whether adducts or fragments (see below). Therefore, we paid close attention to these phenomena when compiling the Consensus Annotation Tables and reassigned 28.2% of the reported features. Of the 610 initial features in the Merged Annotation Table, the ion species was confirmed based on mass differences in 415 cases (68.0%), while 172 reassignments were proposed, and 19 features were declared unresolved (Figure 2 and SI-01 Figures S25–S26). A number of features were incorrectly assigned by the individual teams as [M + H]^+^, and a shift toward other adducts was observed in the new assignments reported in the Consensus Annotation Table (Figure 2).

**Figure 2:**
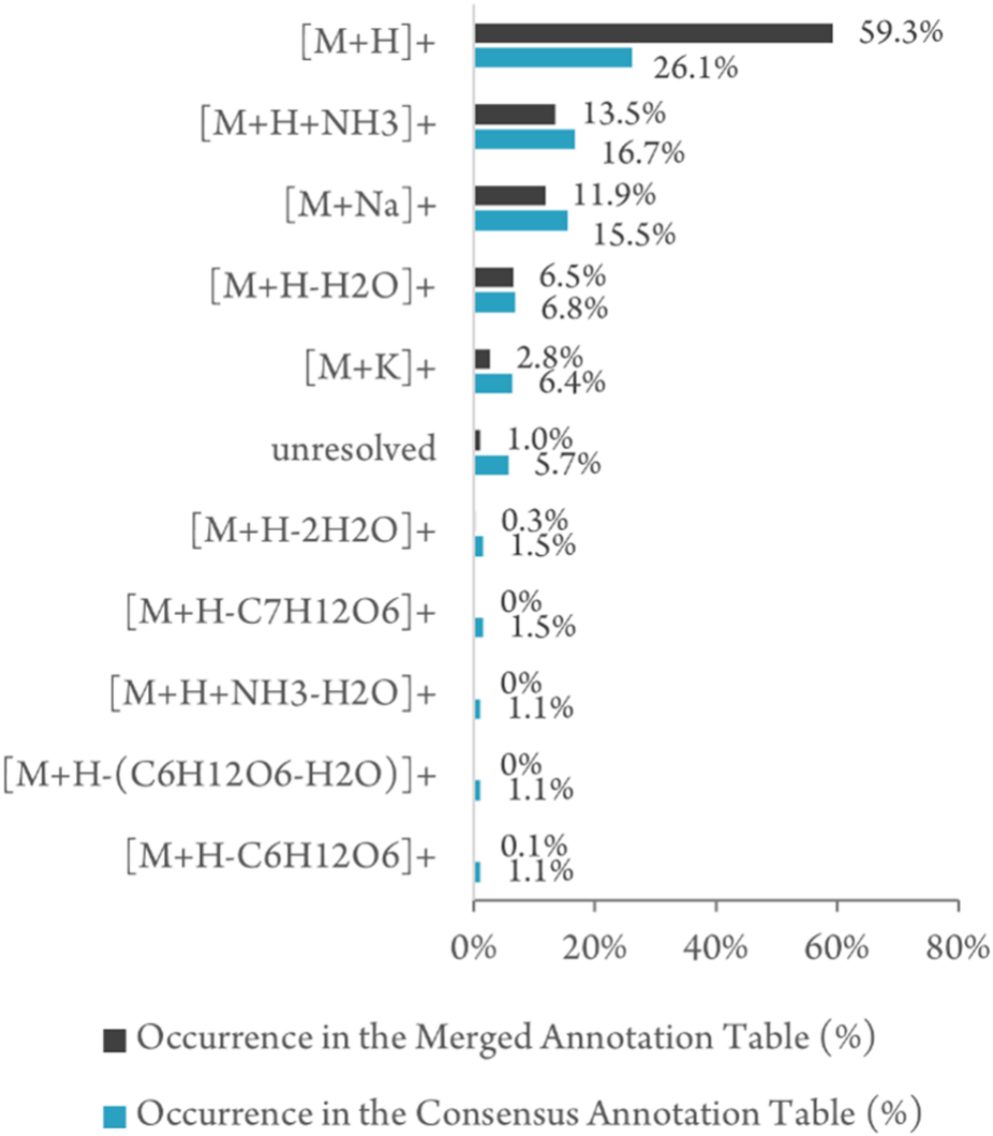
Histogram of the ion species description proposed by the 8 teams for the features reported in the Merged Annotation Table compared to the assignments in the final Consensus Annotation Table. The results indicate that teams were biased toward [M + H]^+^ ions in their original assignments (black bars) and that reassignment (blue bars) caused a shift toward other ion species (see SI-01 Figures S25–S26).

A second important category of reassignments was based on taxonomic information. In the Merged Annotation Table, 24.8% of annotations were taxonomically relevant (SI-01 Figure S29). The percentage was increased to 66.3% in the Consensus Annotation Table in the feature analysis (SI-01 Figure S30) and to 62.2% in the analyte analyses, as many level 5 features were adducts or in-source fragments linked to taxonomic annotations (SI-01 Figure S31). A confidence level of 1 (confirmation with standards) was achieved for 13 analytes, 2 (experimental MS/MS match) for 38 analytes, 3 (*in silico* MS/MS match) for 38 analytes, 4 (structural class) for 23 analytes and 5 (MS1 match) for 15 analytes (SI-01 Table S2 and Figure S31). Through the manual interpretation that led to the Consensus Annotation Table, the proposed chemical classes were confirmed for 387 features (63.4%), while 204 features (33.4%) were assigned to other classes (including the specification that the annotation was a glycoside as defined in NP classifier) (SI-01 Figure S27–S28). Of the reassignments, 63 were reassigned to ergostane steroids and 45 to ergostane steroid glycosides.

### LogP to Assess Consensus Annotation Improvement

Analytes with higher LogP values are more nonpolar and as such would be expected to have higher retention times using reversed-phase chromatography.^54^ To determine whether the accuracy of the annotations was improved in the consensus annotation compared to the original annotations proposed, calculated LogP values (cLogP) were plotted for the analytes associated with each feature as a function of chromatographic retention time (SI-01 Figure S24). For the commercial standards (Figure S24A), a linear relationship was observed between cLogP and retention time (*R* ^2^ 0.7728). For the reported features in the Merged Annotation Table, poor linearity was observed (*R* ^2^ 0.4892, Figure S24B). Linearity was much improved in the Consensus Feature Annotation, with fewer outliers and *R* ^2^ 0.6749 (Figure S24C). These results suggest that the data interpretation performed to arrive at the consensus annotations improved the annotation accuracy.

### Comparison of the Annotations from Individual Laboratories to the Consensus Annotations

“Consensus Annotation Scores” were assigned at both the feature level (SI-01 Figure S22) and at the analyte level (Figure 3). We cross-checked the results for each individual team against the Consensus Annotation Table (SI-03 Tables S1–S3) in two different ways: by comparing against all analytes in the consensus list (Figure 3A) and by comparing only against the analytes that had been confirmed by standards (Figure 3B). Importantly, we note that teams did not have access to the data from the standards when making their annotations, so their data analysis was truly untargeted – the use of standards was employed only after the individual teams had submitted their results to check the accuracy of the proposed annotations. The selection of standards included only some chemical compound classes and was not exhaustive.

**Figure 3:**
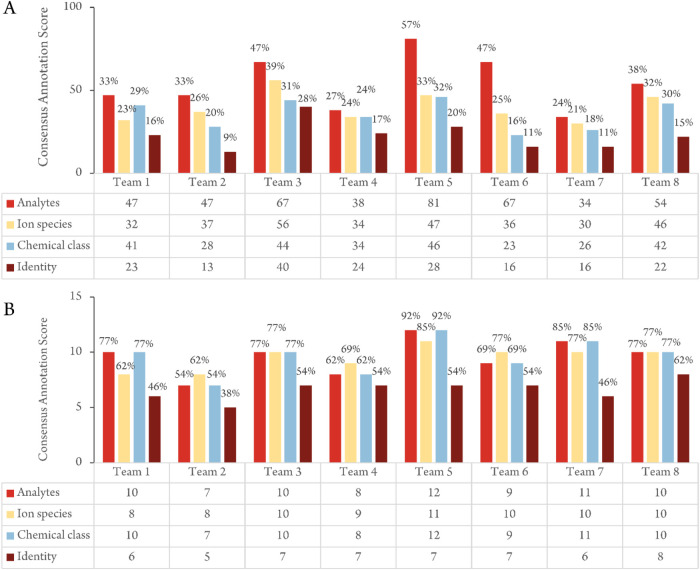
Consensus Annotation Score analysis (SI-01 Figure S21) assesses the overlap between the lists provided by the 8 teams and the Consensus Annotation Table. A total of 142 analytes (unique chemical entities) were annotated in the Consensus Annotation Table after grouping all of the features. “Analyte” refers to the number of analytes reported by a given team that overlaps with those included in the Consensus Annotation Table, while “Ion species,” “Chemical class,” and “Identity” refer to the number of analytes with these elements that match the elements in the Consensus Annotation Table. For example, in Panel A, Team 1 reported 47 (33%) of the 142 analytes included in the Consensus Annotation Table, and 32, 41, and 23 of these analytes, respectively, were reported to have the same ion species, chemical class, and identity assigned in the Consensus Annotation Table. Panel A shows the analyses for all analytes, and panel B for the 13 confirmed by comparison with standards.

The percentages of reported analytes by individual laboratories for all analytes (24–57%. Figure 3A) were lower than those reported for analytes confirmed by standards (Figure 3B). This improvement could be explained by the analytes for which standards are available having a greater peak area in the analysis and therefore being easier to pick out. Additionally, commercially available analytes are better documented in existing databases. The percentage of overlap between individual teams and the consensus table generally decreased in going from feature to ion species to chemical class to identity (Figure 3). This is to be expected; there should be a higher level of agreement on the presence of a given feature in the dataset than on its identity.

### Misassignment of Ion Species as a Source of Annotation Errors

MS data are often used to determine the molecular formula of an analyte and subsequently consult chemical structure databases.^27^ This practice assumes that the electrospray ionization (ESI) source mainly produces the protonated or deprotonated molecule (depending on the instrument polarity). Although ESI is a softer ionization technique than electron impact, fragmentation and adduct formation still occurs.^55^ With this study, we observed that in-source fragmentation and clustering phenomena sometimes led to the absence of the protonated molecule and often generated many redundant features. When the data from the individual teams was compared to the Consensus Annotation Table, the most common error was incorrectly grouping features belonging to one metabolite and assigning different analyte identities to redundant features. Overall, while 59.3% of features proposed by participants were protonated species ([M + H]^+^), only 26.1% were assigned as protonated species in the consensus annotation, highlighting a bias in annotation strategies (Figure 2 and SI-01 Figures S25–S26). The most frequent cases of misassignment were for ammonium adducts ([M+H+NH_3_]^+^, 31 cases), water losses ([M+H–H_2_O]_,_^+^ 29 cases), and sodium adducts ([M + Na]^+^, 17 cases). In-source fragments (*e.g*. the loss of *O*-glycoside, quinic acid, or sulfate) were also commonly misassigned (69 cases). The following examples demonstrate a few cases in which teams disagreed on the assignment of features for several analyte classes.

### Withanolides

Six withanolide standards were used to confirm the identities of *W. somnifera* extract features (SI-01 Figure S36). Four withanolides were detected in the extract with high-quality features (withaferin A, withanone, and withanosides IV and V). Withanolides A and B were detected only in trace amounts, but the data for these standards did enable the rejection of some annotations proposed by the teams at incorrect retention times (13 cases). Annotation of withaferin A achieved the best agreement; it was reported by the 8 teams with the same chemical class. Seven out of 8 teams reported its protonated feature ([M + H]^+^ calc. *m*/*z* 471.2741), while one team reported an adduct ([M+K]^+^ calc. *m*/*z* 509.2306) (SI-01 Figures S33–S34). Four teams correctly reported the presence of withanone, but a lower agreement than for withaferin A was reached (Figure 4 and SI-01 Figure S35).

**Figure 4:**
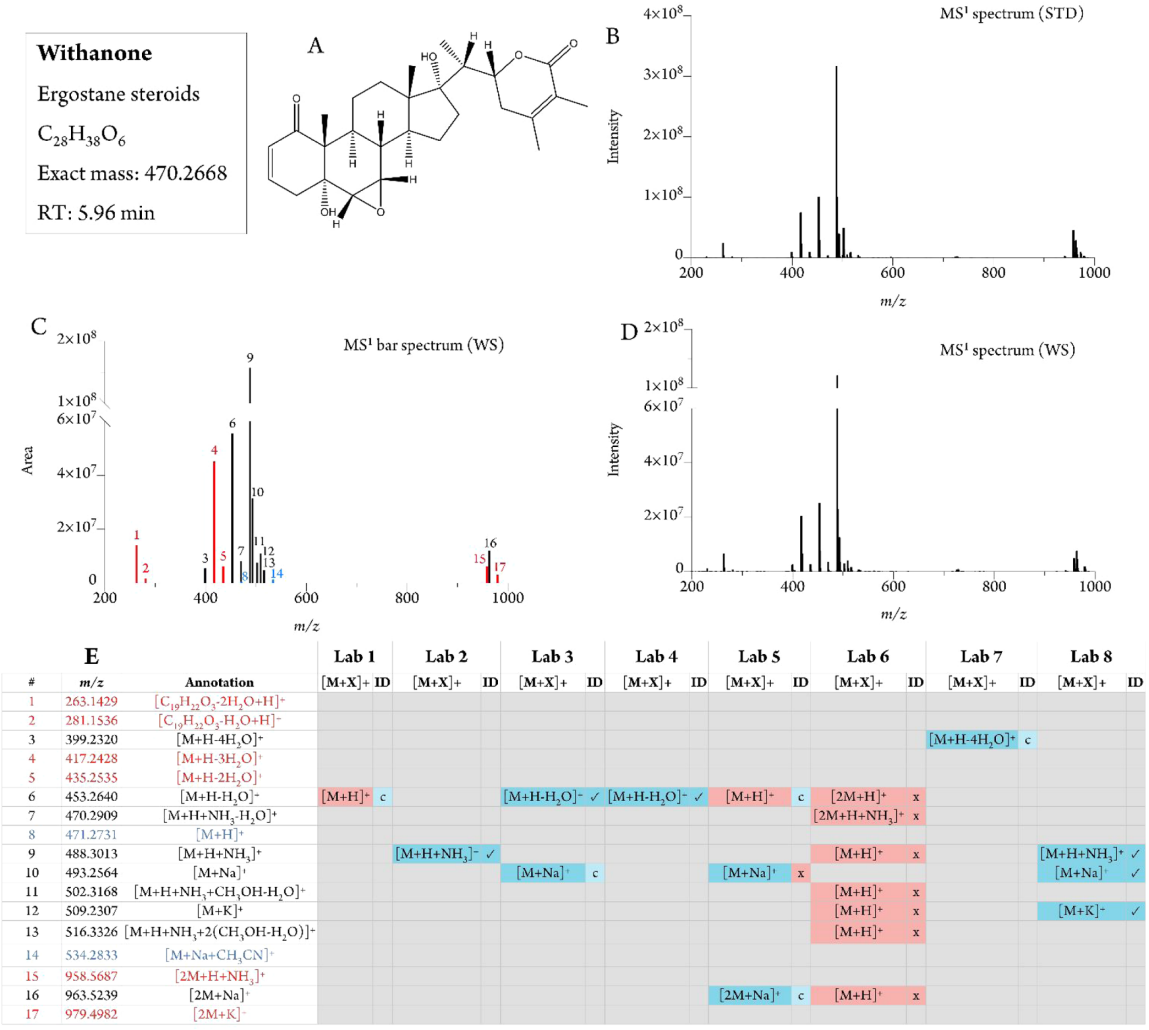
LC-MS data and annotation of withanone (confidence level 1A). (A) Structure of withanone, (B) MS spectrum of the pure standard, (C) Ion species description illustrated with a bar plot that represents the peak area of the features associated with one analyte as a function of mass-to-charge ratio (*m*/*z*), (D) MS spectrum of withanone in the *Withania somnifera* extract, and (E) annotation of its features and their detection by the 8 teams. [M+X]^+^ means the ion species, and ID means its identity (“x” false annotation, “√” correct annotation, “c” correct chemical class but false annotation; blue fill color means correspondence with the consensus annotation, red fill color means no correspondence). The features in red font were not reported by any teams but were detected in our manual interpretation and by comparison with standards, and the ones in blue font did not have any MS/MS spectra. See SI-01 Figure S35 for the fragmentation spectra.

The most intense feature for withanone was the ammonium adduct ([M+H+NH_3_]^+^ calc. *m*/*z* 488.3007), followed by the monodehydrated protonated molecule ([M+H–H_2_O]^+^ calc. *m*/*z* 453.2636) (Figure 3). No team reported the protonated molecule, which could be detected *a posteriori* in a trace amount by comparison with the commercial standard. The feature *m*/*z* 488.3013 was reported by three teams, and two teams agreed on the ion species description, chemical class, and annotation. The feature with *m*/*z* 453.2640 was reported by five teams, of which two agreed on ion species description and identity, and four agreed on chemical class (SI-01 Figure S35).

Withanolides are subject to in-source losses of water and of the lactone moiety.^43^ The features representing these fragments are informative for structural assignment at the MS1 level and not useless, redundant features. For withaferin A, we observed a didehydrated protonated feature (*m*/*z* 435.2534), and the features resulting from the loss of the lactone moiety (*m*/*z* 299.1638), further losing one water (*m*/*z* 281.1534) (SI-01 Figure S33). No participants reported these 3 features. For withanone, we observed the successive losses of water up to 4 water losses (*m*/*z* 435.2535, 417.2428, and 399.2320), and also the loss of the lactone moiety and one water (*m*/*z* 281.1536) and two waters (*m*/*z* 263.1429). The tetradehydrated protonated feature was reported by one team, which correctly assigned the ion species and chemical class. Two teams did not include the one and two water losses in their annotation strategies, and only one team included more than two water losses.

Withanosides IV and V are diglycosyl withanolides whose most intense features were the ammonium adducts ([M+H+NH_3_]^+^, calc. *m*/*z* 800.44270 and 784.4478, respectively). The withanoside IV ammonium adduct feature was reported by six teams; four agreed on the ion species description and the chemical class, and two agreed on the identity (SI-01 Table S4). The withanoside V ammonium adduct feature was reported by six teams, all of which agreed on the chemical class (SI-01 Table S4), and five of which agreed on the adduct identity. Consistent with published literature,^43^ for three withanolide standards out of four, the most intense feature was the ammonium adduct. Three of the eight teams did not include ammonium adducts in their annotation strategy, resulting in missed annotations or incorrect assignments of the ammonium adduct as an [M + H]^+^ ion species (Figure 4).

The presence of isomers in *W. somnifera* complicates the task of data annotation. With the same molecular formula as withaferin A and withanone (C_28_H_38_O_6_, calc. [M + H]^+^ *m*/*z* 471.27412), the LOTUS database describes 17 2-dimensional structures for the same species (28 3-dimensional). In the Merged Annotation Table, 40 features were reported with this molecular formula in a retention time window of just under 2 min. Similarly, the molecular formula C_28_H_36_O_5_ was reported 13 times. It could be a protonated molecule (C_28_H_36_O_5_, calc. [M + H]^+^ *m*/*z* 453.2635) (4 2-dimensional structures described in LOTUS). However, this molecular formula could also result from water loss of the previous molecular formula. Furthermore, several features were annotated as sulfated withanolides with a confidence level from 2A to 5. The sulfate loss, combined with water loss, was also putatively assigned, thus increasing the apparent number of features with a molecular formula of C_28_H_36_O_5_. A total of 19 features were reported by the participants with *m*/*z* 453.264, at 7 different retention times. Among them, 10 were reassigned during manual interpretation: we found 3 to be associated with withanone (level 1), and 7 that were putative assignments (levels 3 to 5). Among these 10 reassigned features, 4 were reassigned as [M+H–H_2_O]^+^ instead of the proposed [M + H]^+^ (including 2 for withanone). Two were reassigned as [M + H]^+^ instead of the proposed [M+H–H_2_O]^+^, and 4 were assigned as in-source fragments of sulfated withanolides. Among the 9 features that were not reassigned, 3 were described as [M + H]^+^ and 6 as [M+H–H_2_O]^+^. We are aware that the reassignments in the Consensus Annotation Table are putative. Our description aims to illustrate the complexity of the annotation task and explain why it still represents a bottleneck.

### Flavonol Glycosides

Three flavonol glycosides were confirmed by external standards (kaempferol-3-*O*-rutinoside, rutin, and quercetin-3-rutinoside-7-glucoside) (SI-01 Table S4 and Figure S37). They illustrated an in-source fragmentation pattern, *O*-glycoside fragmentation, that is known but not effectively handled in existing annotation strategies. Five annotation cases included a glycoside and its corresponding aglycone at the same retention time, for example, rutin (diglycoside) and quercetin (its aglycone). Two teams reported the loss of one glycoside as an independent analyte at the same retention time as a diglycoside. Similar errors were observed for cinnamic acid derivatives (SI-01 Table S4). These errors demonstrate a limitation in current MS/MS annotation tools such as GNPS,^9^ SIRIUS,^11^ and MS-Finder:^14^ they do not consider retention time or polarity and, therefore, overlook the fact that it is not possible to have two distinct annotations for compounds of different polarity at the same retention time.

### Summary of Annotation Successes, Challenges, and Recommendations

In recent years, tremendous effort has been devoted to the construction of open-access databases containing thousands of MS/MS reference spectra.^4^ These efforts, in combination with various software tools for data interpretation, enabled the successes reported herein. Each team, using their own methods mainly based on MS/MS annotations, was able to successfully report in the range of 40% of the analytes in the Consensus Annotation Table (Figure 3B), which illustrates the capacities of the annotation pipelines. Nevertheless, this study also suggests room for improvement in the annotation of mass spectrometry metabolomics data. One problem that persists is that, for many small molecules, including plant secondary metabolites, spectra have not been submitted to searchable databases. At the time of this study, we observed that MS/MS spectra were missing from online databases for many compounds present in *W. somnifera*. As a result, annotation efforts based mainly on automatic MS/MS spectral comparison were inefficient. Manually incorporating information from published manuscripts was necessary to increase the number of annotations, particularly for withanolides.^42,43^ The continued submission of MS/MS data to open-access databases^9^ is a critical step toward enabling more effective and rapid annotation of mass spectrometry metabolomics datasets. Toward this goal, MS/MS spectra of all of the analytes detected in this collaborative project with a level 1 confidence were uploaded to GNPS.

Additionally, it is important to check annotations based on annotation databases against the published literature. We note that only one of the participating groups in this collaboration included a literature search as part of their data analysis pipeline (SI-01 Section 4), and that our own analysis of the literature, while time-consuming, considerably improved the quality of the Consensus Annotation.

Another point illustrated by this study is the complexity of the mass spectral data for plant secondary metabolites. The existing annotation strategies do not always effectively address in-source fragmentation and cluster formation, and tend to annotate redundant in-source features as independent analytes. Our study is not the first to point to the assignment of ion species as a challenge for mass spectrometry data interpretation.^3^ Complex ionization mechanisms can occur, making it difficult to link the analyte structure with the ions detected.^56^ The problem of assigning ion species arose in the 2014 version of the critical assessment of small molecule identification (CASMI) contest, which has been organized for the last ten years.^36^ In 2016 and 2022, CASMI artificially addressed this difficulty by providing only spectra from specific, defined precursors.^8,57^

This study illustrates a need to improve methods for grouping features and assigning ion species in metabolomics datasets. Developers, end-users, and reviewers should view mass spectrometry data annotations cautiously, especially when they are not checked with data from authentic standards.^3^ Existing tools, (e.g., CAMERA^28^, MS-CleanR,^30^ BINNER,^26^ MolNotator,^52^ and ion identity networking (IIN)^31^) could be better disseminated and implemented. These efforts should be prioritized to improve annotation at the MS level, providing additional information to supplement that used by widely used MS/MS fragmentation interpretation tools. One possible strategy toward this goal is the inclusion of predicted in-source fragments (which can be formulated based on MS/MS spectra) during the adduct searches; however, a downside of this approach is the possibility of a higher false discovery rate. Steps such as automatic prediction of an annotation’s LogP could be added to annotation pipelines, and alerts could be implemented when two annotations share a retention time but have distinct cLogPs. It may also be necessary to consider strengthening existing tools by making them more user-friendly. The literature is replete with new scripts,^4^ all adding value. Encouraging projects that prioritize the development of tools for community use seems to us to be a recommendable step.

A final challenge encountered in this study was assigning the annotation confidence levels. Extensive efforts have previously been devoted to devising confidence levels for annotations of mass spectrometry data,^5,20–22^ however, we encountered several limitations in applying the established frameworks. These scales consider the type of analytical information used to assign structure but tend to put less emphasis on the taxonomic information, i.e., whether the compound reported has previously been observed in the same genus and species, a consideration that is particularly important when working with nonhuman organisms. Another limitation of existing confidence levels is that the data captured at the MS level tends not to be included. For example, it is likely that an ion species assigned based on the observation of multiple adducts and in-source fragments is of higher confidence than one observed based on a single feature. Such a strategy was employed by Team 7, but this information could not be captured in the existing annotation scales. Additional revisions may be required to improve the methods by which annotation scores are calculated to capture these nuances. However, even given these limitations, it is very important to include the confidence level when reporting annotations for mass spectrometry data.^3^

### Lessons About Study Design

This study is the first, to our knowledge, to compare annotations of mass spectrometry datasets across laboratories using an untargeted design. The findings provided valuable insights about the current challenges and opportunities in the annotation of mass spectrometry datasets and also pointed to several lessons that might improve the design of future interlaboratory annotation comparisons. The first of these lessons is that asking participants to report the annotations at the analyte level without including a full list of observed redundant features was a limitation. Comparison of feature lists would have enabled us to better understand how limitations with peak picking and filtering might impact the annotation accuracy. Second, we acquired several distinct datasets on both Orbitrap and Q-ToF mass spectrometers, hoping to recruit participants who work with both platforms. We required a minimum of one positive ionization dataset to be annotated by volunteer participants. The disadvantage of asking participants to choose just one of many datasets was that only one dataset was annotated by enough teams to enable a comparison. Furthermore, making annotation of the negative ion mode datasets optional meant that these negative ionization mode datasets were not annotated by enough teams for inclusion. A future study might focus on a single set of matched positive and negative ion data, demonstrating how the inclusion of both improves the annotation accuracy. Future studies comparing the results on the Orbitrap and Q-ToF platforms would also be possible.

Overall, while the study design employed here enabled a big-picture comparison of how different teams annotate untargeted metabolomics data, the complexity and number of steps involved in the annotation process made it difficult to compare individual aspects of the various pipelines. For example, the participating teams could use any publicly available, purchased, or in-house database of MS/MS spectra. Future studies aimed to assess the strengths and weaknesses of different data interpretation pipelines might benefit by restricting the participants to a single database. Such an approach would decouple problems that are associated with the pipeline itself from those that arise from errors or limitations in the available databases. Future collaborative studies might also benefit from isolating individual aspects of the annotation process for comparison. For example, multiple teams could peak-pick and filter the same dataset, annotate the same feature list, or group redundant features from a single feature list. Comparison of the results from these limited tasks could help identify the strengths and pitfalls of different pipelines. The application of the *Annotation Agreement Score* approach employed here to compare datasets across laboratories could facilitate such future studies, and these could be carried out using the datasets acquired during this study, which are all available for download through the MassIVE repository.

## Conclusions

An untargeted strategy was developed to compare the annotation results of eight teams and generate consensus annotations. In interpreting the results of this study, we sought not to compare or rank the methods used by the individual teams but instead to take advantage of the strategy deployed by each team to create a comprehensive description of the chemistry of a single extract. Relying on the different approaches and strengths of the teams did indeed result in the identification of many more analytes than would have been possible by any team alone. The dataset we have produced – a carefully cross-checked list of analytes detected in an extract of the plant *W. somnifera* – is expected to be valuable as a benchmarking tool for future metabolomics studies. The raw data employed in this collaborative study are available (See Data Availability Statement section), and the annotation tables are in the SI-03 file. Our identifications are only putative and should be interpreted in accordance with the indicated confidence levels. Even annotations for which there is a high level of agreement across teams could still be incorrect or only partially correct, and we do not wish to imply that agreement across teams (precision) is the same as agreement with the true structure (accuracy). However, the data provided showing improved correlation between retention time and cLogP (SI-01 Figure S24) for the consensus annotations implies that the accuracy of structural assignment was improved by cross-comparing results across teams.

Comparison of the results reported by different groups has also enabled us to highlight some of the major challenges in metabolomics data interpretation and recommend future strategies to address them. Our findings suggest that it is easy in untargeted mass spectrometry studies to overestimate the complexity of a given sample. Much of the analysis in our field continues to occur at the feature level, and it is easy to assume that a dataset composed of many features represents a very high number of analytes. Mass spectrometry metabolomics enabled the detection of a large number (142) of analytes in a single sample. However, there were many cases in this study where incorrect assignment of feature identities or inclusion of features from interferences (i.e., components of the blank) caused an inflated peak list that did not reflect the number of analytes present. The nature of untargeted mass spectrometry metabolomics, where the analyst seeks to capture all metabolites detectable in a single sample rather than focusing on a few known ones, makes it very easy to make errors of this type.

Mass spectrometry is a powerful tool for querying samples with very low limits of detection. This study suggests that progress has been made toward translating the information obtained by these types of analyses to accurately describe samples in terms of their chemical constitution, and that the quality of data annotation can be improved by relying on multiple teams with complementary approaches. Nonetheless, there is still considerable room for improvement in untargeted mass spectrometry metabolomics data annotation.

## Supplementary Material











## Data Availability

The datasets employed in this study can be found online in the MassIVE repository, and their accession number are as follows: Orbitrap dataset: MSV000089047 (temporary password during the submission process: **orbi**). Q-TOF dataset: MSV000089033. Standards dataset: MSV000097002.

## Notes

Table of terminology and definitions; section with supplements to the experimental section; tables with annotation confidence level definitions; narrative descriptions of the strategies employed by the participants and schematic representations of these strategies; figures about the data preprocessing applied by the participants; figures which describe the annotations made by the participants; figures to detail the workflow to generate the Consensus Annotation Table (including the Annotation Agreement and Consensus Scores); figures to describe the Merged Annotation Table and the Consensus Annotation Table; a table grouping the annotations obtained for the commercial standards; and figures with the features in the metabolite profiling of *W*. *somnifera* (PDF)
The instructions sent to all invited laboratories (PDF)
Annotation tables (XLSX)
List of adducts, neutral losses, and gains compiled through this study, and the lists from two teams (XLSX)
Inclusion list used for the DDA data collection (XLSX)
