# Supplementary material for: Multilaboratory Untargeted Mass Spectrometry Metabolomics Collaboration to Identify Bottlenecks and Comprehensively Annotate A Single Dataset
Source: Anal Chem. 2025 Jul 22;97(30):16110–22. doi: 10.1021/acs.analchem.4c05577 (PMC12332825; doi:10.1021/acs.analchem.4c05577)
Supplement: Supplementary file 1 [file ac4c05577_si_001.pdf]

Supporting information

A multi-laboratory untargeted mass spectrometry metabolomics collaboration to identify bottlenecks and comprehensively annotate a single dataset

Joelle Houriet[1‡], Preston K. Manwill[1‡], Armando Alcázar Magaña[2], Victoria M. Anderson[1], Mehdi A. Beniddir[3], Samuel Bertrand[4, 5], Jaewoo Choi[6], Trevor N. Clark[7], Leonard Foster[2], Maria Halabalaki[8], Alan K. Jarmusch[9], Niek F. de Jonge[10], Aswad Khadilkar[11], John B. MacMillan[11], Claudia S. Maier[6], Luke C. Marney[6], , Guillaume Marti[12, 13], Eleni V. Mikropoulou[8], Damien Olivier-Jimenez[14], Amélie Perez[12, 13], Justin J. J. van der Hooft[10, 15], Mitja M. Zdouc[10], Roger G. Linington[7], Nadja B. Cech[1]*

‡ co-first authors

[1] Department of Chemistry & Biochemistry, University of North Carolina at Greensboro, Greensboro, North Carolina 27402, USA. [2] Life Sciences Institute, Department of Biochemistry and Molecular Biology, University of British Columbia, Vancouver BC, Canada, V6T 1Z4

[3] Équipe "Chimie des substances naturelles" BioCIS, CNRS, Université Paris-Saclay, 17, avenue des Sciences, 91400, Orsay, France

[4] Nantes Université, Institut des Substances et Organismes de la Mer, ISOMER, UR 2160, 44000 Nantes, France,

[5] Nantes Université, École Centrale Nantes, CNRS, LS2N, UMR 6004, 44000 Nantes, France

[6] Department of Chemistry, Oregon State University, Oregon 97331, USA

[7] Department of Chemistry, Simon Fraser University, Burnaby, BC V5A 4Y8, Canada

[8] Division of Pharmacognosy and Natural Products Chemistry, Department of Pharmacy, National and Kapodistrian University of Athens, 157 71 Zographou, Greece

[9] Immunity, Inflammation, and Disease Laboratory, Division of Intramural Research, National Institute of Environmental Health Sciences, National Institutes of Health, Research Triangle Park, NC 27709, USA

[10] Bioinformatics Group, Wageningen University & Research, Wageningen, 6708 PB, the Netherlands

[11] Department of Chemistry & Biochemistry, University of California Santa Cruz, Santa Cruz, California 95064, USA

[12] Laboratoire de Recherche en Sciences Végétales, Metatoul-AgromiX Platform, Université de Toulouse, CNRS, INP, , 31320, Auzeville-Tolosane, France

[13] MetaboHUB-MetaToul, National Infrastructure of Metabolomics and Fluxomics, Toulouse 31400, France

[14] Center for Proteomics and Metabolomics, Leiden University Medical Center, 2333 ZA Leiden, the Netherlands

[15] Department of Biochemistry, University of Johannesburg, Johannesburg 2006, South Africa

*Email: nadja_cech@uncg.edu

KEYWORDS: metabolite annotation, MS^1 in-source features, mass spectrometry metabolomics, preprocessing, Withania somnifera, comparative study

Contents

Section 1. Terminology and definitions 4
Section 2. Supplements to the experimental section 5
Section 3. Confidence levels 7
Section 4. Narrative descriptions of the strategies of the participants 8
Section 5. Strategy overview 16
Section 6. Survey: data preprocessing 20
Section 7. Description of annotations for each individual team 23
Section 8. From annotations of individual teams to consensus annotations 27
Section 9. Description of annotations 34
Section 10. Standard annotations 41
Section 11. Interpretation of the fragmentation spectra of the withanolides 43
Section 12. Viewing features in the metabolite profiling 51
Section 13. References 52

Table S1: terminology and definition of keys terms (alphabetical order) ... 4
Table S2: definition of the confidence levels related to mass spectrometry data ... 7
Table S3: definition of the confidence sublevel related to orthogonal Information ... 7
Table S4: annotation of the features related to the analytes confirmed by external standards and their detection by the 8 teams. ... 47

Figure S 1. ... 16
Figure S 2. ... 16
Figure S 3. ... 17
Figure S 4. ... 17
Figure S 5. ... 18
Figure S 6. ... 18
Figure S 7. ... 19
Figure S 8. ... 19
Figure S 9. ... 20
Figure S 10. ... 20
Figure S 11. ... 21
Figure S 12. ... 22
Figure S 13. ... 23
Figure S 14. ... 24
Figure S 15. ... 25
Figure S 16. ... 26
Figure S 17. ... 27
Figure S 18. ... 28
Figure S 19. ... 30
Figure S 20. ... 31
Figure S 21. ... 32
Figure S 22. ... 33
Figure S 23. ... 34
Figure S 24. ... 35
Figure S 25. ... 36
Figure S 26. ... 37
Figure S 27. ... 38
Figure S 28. ... 39
Figure S 29. ... 40
Figure S 30. ... 40
Figure S 31. ... 40
Figure S 32. ... 41
Figure S 33. ... 42
Figure S 34. ... 42
Figure S 35. ... 42
Figure S 36. ... 45
Figure S 37. ... 46
Figure S 38. ... 51

Section 1. Terminology and definitions

Table S1: terminology and definition of keys terms (alphabetical order)

Terms	Definition
Alignment	Alignment algorithms group features with similar *m/z* and retention time (within defined tolerance ranges) among different samples by classifying them into a consensus *m/z* and retention time in all samples in the dataset. The resulting feature list provides an overview of the presence or absence of each feature in all samples in the data set [1].
Analyte	The specific molecule (chemical entity) present in the sample that can be detected in the form of multiple in-source redundant features.
Annotation	"Annotation" referred to the putative or partial metabolite identification of features or analytes in metabolite profiling or fingerprints of complex mixture. [2]
Feature height	Signal intensity, which becomes the peak height after deconvolution.
Feature (peak)	At a minimum, a feature is defined by its mass to charge ratio (*m/z*) and its retention time. In a more extensive way, it must contain ≥4 datapoints (or scans)[3] and must present a Gaussian shape. Some authors require the presence of an isotope pattern [4].
Filtering	Strategies in place to eliminate features, mainly to reduce noise (see the entry "noise").
Gap-filling	Gap-filling is an optional process performed in some data preprocessing workflow to compensate for deficient feature detections and/or misalignment. During gap-filling, the raw data of the aligned samples is re-examined to see if the features detected in one sample are present in the others within defined *m/z* and retention time tolerances [1].
High-quality feature	Defined as a feature (see entry "feature") detected in a series of at least three replicates with a relative standard deviation (RSD) smaller than 30% and absent in blank analyses.
Identity	The two-dimensional structure(s) assigned to an analyte in the process of annotation.
In-source redundant features	By-product ions of ESI (neutral losses (fragments), neutral gains, attachment of other ions (adducts), dimers, heterodimers (complexes). Often referred to as "in-source degradation products", "redundant features" or "degenerate features" [5,6].
Low-quality feature	Defined as a peak detected in triplicates with an RSD higher than 30% or detected in blank analyses.
MS bar spectrum	A bar plot reconstituted from peak descriptors obtained after LC-HRMS data processing. The peak area of features associated to one analyte are represented in bar form, as a function of mass-to-charge ratio (*m/z*).
MS spectrum	MS spectrum is obtained through peak-picking and is the result of averaging the full scan spectra over the chromatographic peak [7].
Noise	False positive detected features caused by 1) random and 2) chemical noise [8]:<br>1) Random noise is attributed to the detector, for example residuals of the Fourier transformation function with an Orbitrap mass analyzer.<br>2) Chemical noise is caused by background signals present in eluants, materials, and laboratory air that are not due to components of the sample.
Peak-picking	Peak-picking is used to break up the continuous signal of mass spectrometer response versus time into discrete "features" (see below) defined by their mass-to-charge ratio (m/z) and retention time [9]. Also called "feature finding".

Section 2. Supplements to the experimental section

Chemicals. The pure substance (>98%) used as reference compounds were purchased from the following suppliers: withanolide A (32911-62-9), withanone (27570-38-3), withaferin A (5119-48-2), withanolide B (56973-41-2), withanoside V (256520-90-8), and withanoside IV (362472-81-9) from Chromadex (Longmont, CO), benzoic acid (65-85-0) from Sigma (St. Louis, MO), ferulic acid (1135-24-6) and tropine (120-29-6) from Cayman Chemical (Ann Arbor, MI), and *trans*-caffeic acid (331-39-5) from Tokyo Chemical Industry (Portland, OR).

LC-MS data acquisition. Two different platforms were used: 1) an Acquity UPLC I-Class coupled to a Waters Synapt G2-Si (Q-TOF), and 2) an Acquity UPLC I-Class coupled to a Thermo Q-Exactive Plus Orbitrap mass spectrometer (Orbitrap).

Q-TOF: Data were acquired on a Synapt G2-Si hybrid quadrupole-traveling wave ion mobility (TWIM)-time-of-flight (TOF) mass spectrometer equipped with an electrospray ionization (ESI) source. Acquisitions were made in either ESI+ for positive ionization mode or ESI – for negative ionization mode; voltage 3.0 kV; cone voltage 35 V; source offset 50 V; source temperature 150°C; desolvation temperature 300°C; cone gas flow 30 L/h; desolvation gas flow 600 L/h. Mass measurements were recorded using either Full Scan (FS), FS – Data Dependent Acquisition (DDA), or FS – Data Independent Acquisition (DIA) experiments. Detection was performed in the *m/z* range 50-1500 with a scan rate of 0.1 Hz in both MS1 and MS/MS experiments. Leucine enkephalin was employed as the lockspray solution at a concentration of 200 pg/µL at 0.10 Hz with scans to average set to 3. In DDA mode precursor selection was set to 3 with an intensity threshold of 5.0×10^3 for selection and a real-time exclusion set to 3.0 seconds. Collision energy for DDA and DIA was set to 30 eV. Argon was used as the collision gasEach DDA dataset for the *W. somnifera* extract consisted of an average of 1,000±4 MS1 and 2941±5 MS/MS scans (reported as average ± standard deviation, with the average calculated across triplicate analyses).

Orbitrap: Data were acquired on a Q Exactive Plus Hybrid Quadrupole-Orbitrap mass spectrometer (Thermo Fisher Scientific) equipped with a heated electrospray ionization (HESI) source. The ion source was operated in either positive or negative ionization mode using the following parameters: spray voltage of 3.5 kV, heater temperature of 450 °C, capillary temperature of 275 °C, S-Lens RF level of 50, sheath gas, auxiliary gas, and spare gas of 55, 15, and 3 (arbitrary units), respectively. Nitrogen was used as the source gas and as the collision gas. Mass measurements were recorded using either Full Scan (FS), FS – Data Dependent Acquisition (DDA), or FS – Data Independent Acquisition (DIA) experiments. Detection was performed in the *m/z* range 100-1500 with a resolving power of 35,000, an AGC target of 1.0 x 106, and a maximum injection time (IT) of 100 milliseconds. DDA MS/MS events were performed on the three most intense precursor ions detected in the full scan MS^1 event (Top3 experiment) with an inclusion list of masses of known compounds of interest (SI-05) and the instrument set to pick other ions if idle. Following MS/MS acquisition, precursor ions were placed in a dynamic exclusion list of 3.0 s. The DDA occurred with a resolving power of 17,500, an AGC target of 1.0 x 105, a maximum IT of 50 milliseconds, an isolation window of 1.0 Da, a collision energy of 30 eV, and an intensity threshold of 1.6 x 105. The DIA occurred with a resolving power of 35,000, an AGC target of 1.0 x 106, a maximum IT of 100 milliseconds, and a collision energy of 30 eV. Each DDA dataset for the *W. somnifera* extract consisted of an average of 2,113±4 MS1 and 5,683±26 MS/MS scans (reported as average ± standard deviation, with the average calculated across triplicate analyses).

Data conversion: All .raw files were converted to .mzML using ProteoWizard MSConvert [10] using default settings with the following exceptions: binary encoding precision set to 32-bit, did not use zlib compression, the filter Peak Picking for vendor msLevel 1-2 added as first step of processing.

Data preprocessing. The mzML files of the Orbitrap DDA in positive ionization were loaded to MZmine 2.53 [11]. The ADAP workflow [12] was employed with the following parameters: the mass detection step kept the ions that were above a noise level of 5×10^3 (and 0 for MS/MS) (centroid detector). Chromatogram builder was employed with minimum group size in numbers of scan of 5, an *m/z* tolerance of 0.004 Da, a group intensity threshold of 5×10^3, and a minimum highest intensity of 1×10^4. The chromatogram deconvolution was performed with a single noise threshold of 10, the coefficient over area threshold was set at 100, the RT wavelet scales from 0.00 to 0.04 min, the peak duration range from 0.0 to 1.00 min, and a minimum peak height of 1×10^4. The MS/MS scan pairing parameters were set at 0.025 Da and 0.08 min. The feature lists were deisotoped using the isotope peak grouper with an *m/z* tolerance of 0.003 Da, a RT tolerance of 0.01 min, a maximum charge of 3, and the representative isotope used was the most intense.

After deconvolution, the feature lists were aligned. *Withania somnifera* extract and the two series of blanks were aligned for comparison with participants. These analyses were then aligned with the standard analyses for identification purposes. Standard analyses were performed in two batches. Feature alignment was obtained using the "join aligner" method with an *m/z* tolerance of 0.003 Da, an absolute RT tolerance of 0.05 min, a weight for *m/z* of 2, a weight for RT of 1, and an isotope pattern comparison of a minimum of 50 %. Gap-filling was applied with the "same RT and *m/z* range gap filler" module with an *m/z* tolerance set at 0.003 Da. Gap-filling was followed by the "peak filter" module to keep features with at least 5 datapoints and with a peak height higher than 1×10^4. Then, the "duplicate peak filter" module was applied with an *m/z* tolerance of 0.001 Da and an RT of 0.01 min to eliminate duplicates. The "features list rows filter" module was first employed to reduce the feature list to features detected from 0.8 to 9 min. Then to reduce the feature list to features with an MS/MS spectrum, and finally to keep feature with at least two isotopes in their isotope pattern (note that these steps were done one by one to document the number of features removed at each stage, but they could be done in one step).

The "identification" module was employed to annotate the peak list, including the "custom database search" which compiled the $[M+H]^+$ and some common adducts of compounds previously described for the same botanical species and genus, and the "adduct search" which compiled adducts referenced in several sources [5, 13]. This compilation contains common charge carriers (H^+, Na^+, K^+, Li^+), neutral gains and losses, and a combination of them, presented according to [5].

Next, the feature lists were exported and filtered in Excel 16. Mean, standard deviation, and relative standard deviation (RSD) were calculated for each set of replicates (*Withania somnifera*, the extraction blanks, the solvent blank (named waste blank), and the standards) and for all aligned samples. Blank filtering consisted in 1) eliminating features not detected in *Withania somnifera* samples but only in blanks (mean in *Withania somnifera* samples equal to 0), 2) eliminating features detected in all samples with an RSD below 30% (real features detected in all samples, i.e. solvent contaminants), and 3) eliminate feature with a blank ratio equal to or higher than 80% (average feature area ratio between each series of blanks and samples of interest (*Withania somnifera*, and standards) based on [14]). Then, the RSD filtering eliminated *Withania somnifera* features with an RSD above 30%.

To assess participant's annotations, we proceeded first by checking if we had also detected the feature in our final feature list but also in the feature list before filtering, which allowed us to classify the features not retained for the comparison, and therefore the consensus annotation table.

Section 3. Confidence levels

Below are the tables with the confidence levels proposed to the participants to estimate their annotations.

Table S2: definition of the confidence levels related to mass spectrometry data

MS Level	Definition
0	Unambiguous 3D Structure: Isolated Compound including full stereochemistry, following natural products guidelines, determination of 3D structure.
1	Confirmed structure by reference standard. MS, MS/MS, RT, reference standard
2	Probable structure (literature, library): Unambiguous matching literature or library MS/MS spectrum.
3	Probable structure (experimental, in silico): Structure determined based on MS, MS/MS fragments sub-structure matching with experimental data or in silico fragmentation.
4	Tentative candidate: Class of structures based on MS, MS/MS, experimental data, but positional isomers cannot be distinguished.
5	Unequivocal molecular formula based on MS, isotopes, adducts, ring double bound equivalents (RDBE)
6	Exact mass of interest, no proposed structure or formula.

Level 0 to 1 were not possible with the data provided. Participants were asked to not include level 6 analytes in their annotation table.

Table S3: definition of the confidence sublevel related to orthogonal Information

Orthogonal sublevel	Definition
a	Previously described for the same Species
b	Previously described for the same Genus
c	Previously described for the same botanical Family
d	Match with UV spectrum in literature/library
e	Retention time prediction
f	Ion mobility
etc.	

Sub-level “f” and “d” were not possible with the data provided.

Section 4. Narrative descriptions of the strategies of the participants

Ten teams participated in the project, including the two teams that initiated the project. Five teams were from Europe: Prof. Mehdi A. Beniddir (BioCIS, University of Paris-Saclay, France), Dr. Samuel Bertrand (ISOMer, Nantes Université, France), Prof. Maria Halabalaki and Dr. Eleni V. Mikropoulou (Division of Pharmacognosy and Natural Products Chemistry, Department of Pharmacy, National and Kapodistrian University of Athens, Greece), Dr. Guillaume Marti (Metatoul-AgromiX platform, LRSV, University of Toulouse 3, France), and Dr. Justin J. J. van der Hooft, Niek F. de Jonge, and Dr. Mitja M. Zdouc (Bioinformatics Group, Wageningen University & Research, the Netherlands) together with Dr. Damien Oliver-Jimenez (Leiden University Medical Center, Leiden, the Netherlands). And five from North America: Prof. Nadja B. Cech, Dr. Joelle Houriet, Dr. Preston K. Manwill, and Dr. Victoria Anderson (Department of Chemistry and Biochemistry, University of North Carolina at Greensboro, USA), Prof. Leonard Foster and Dr. Armando Alcazar Magana (Life Sciences Institute, Department of Biochemistry and Molecular Biology, University of British Columbia, Canada), Dr. Alan K. Jarmusch (National Institute of Environmental Health Sciences, USA), Prof. Roger Linington and Dr. Trevor Clark (High-Throughput Screening and Natural Products Discovery, Department of Chemistry, Simon Fraser University, Canada) and Prof Claudia S. Maier and Dr. Luke C. Marney and Dr. Jaewoo Choi (Department of Chemistry, Oregon State University, USA).

Team 1. The orbitrap positive DDA mode RAW MS data were converted to .mzML using ProteoWizzard [15] and loaded to MZmine 2.53 [11]. The ADAP workflow [12] was employed with the following parameters: the mass detection step kept the ions that were above a noise level of 1.7^{E}4 (and 1.7^{E}2 for MS/MS). Chromatogram builder was employed with minimum group size in numbers of scan of 5, an *m/z* tolerance of 0.008 Da or 8 ppm, and group intensity thresholds of 1.7^{E}4, and a minimum highest intensity of 1.7^{E}4. The chromatogram deconvolution was performed with a single noise threshold of 50, the coefficient over area threshold was set at 110, the RT wavelet scales from 0.02 to 0.2 min, the peak duration range from 0.02 to 1.0 min, and a minimum peak height of 1.7^{E}4. The MS/MS scan pairing parameters were set at 0.02 Da and 0.1 min. The feature lists were deisotoped using the isotope peak grouper with an *m/z* tolerance of 0.008 Da or 8 ppm, a RT tolerance of 0.08 min, a maximum charge of 2, and the representative isotope used was the lowest *m/z*. Peak alignment was obtained using the "join aligner" method with an *m/z* tolerance of 0.008 Da or 8 ppm, an absolute RT tolerance of 0.08 min, a weight for *m/z* of 20, a weight for RT of 20. Gap-filling was applied with the "same RT and *m/z* range gap filler" module with an *m/z* tolerance set at 0.008 Da or 8 ppm. Then, the "duplicate peak filter" module was applied with an *m/z* tolerance of 0.008 Da or 8 ppm and an RT of 0.08 min to eliminate duplicates. The "identification" module was employed to annotate the peak list, including the "adduct search" which compiled common charge carriers (H^+, Na^+, K^+, ACN^+, CH_3OH^+) using the following parameters: RT tolerance = 0.05 min, *m/z* tolerance of 0.008 Da or 8 ppm, Max relative adduct peak height = 0.05, and the "complex search" using the following parameters: ionization method : $[M+H]^+$, RT tolerance = 0.05 min, *m/z* tolerance of 0.008 Da or 8 ppm, Max relative complex peak height = 0.05. A first peak list was generated by using the "features list rows filter" module to reduce the feature list to features that have been annotated as a complex and/or an adduct, with RT (0.00) and 8.0 min. Then, the "custom database search" which compiled the $[M+H]^+$of compounds previously described for the same botanical species was used to annotated the MS1 data using *m/z* tolerance of 0.001 Da or 5 ppm and 500 min as RT tolerance. Eventually, the rows aligned with the blanks were removed manually. A first .csv file was generated to compile the MS1 annotation to be appended on the molecular network.

To perform a FBMN [16], a second peak list (with no complex, adducts and MS1 annotated features) was generated using the "features list rows filter" to reduce the feature list to features with an MS/M spectrum. A molecular network was created using the online FBMN workflow (version release_28.2) at GNPS [17] (http://gnps.ucsd.edu) with a parent mass tolerance of 0.02 Da and an MS/MS fragment ion tolerance of 0.02 Da. A network was then created where edges were filtered to have a cosine score above 0.65 and more than 6 matched peaks. Further edges between two nodes were kept in the network if and only if each of the nodes appeared in each other's respective top 10 most similar nodes. The spectra in the network were then searched against GNPS spectral libraries. All matches kept between network spectra and library spectra were required to have a score above 0.7 and at least 6 matched peaks. No filtering options were applied. The quantification data were normalized per file using the « Row Sum Normlization (Per File Sum to 1,000,000) and the peak abundances per group were aggregated using Mean. Dereplicator were turned off. The molecular networking data were analyzed and visualized using Cytoscape (ver. 3.6.0) [18]. The GNPS job is accessible here: https://gnps.ucsd.edu/ProteoSAFe/status.jsp?task=9064f4bf35724cd58e708396f06fe593.

The mgf data generated by MZMine were further annotated using MolDiscovery [19] workflow (https://gnps.ucsd.edu/ProteoSAFe/index.jsp?params=%7B%22workflow%22:%22MOLDISCOVERY%22%7D), the job is accessible here : https://gnps.ucsd.edu/ProteoSAFe/status.jsp?task=4a33d646c4cd4fcea22d075fd23a0947. At last, the molecular network was multi-annotated using GNPS, Moldiscovery and MS1 (using taxinomy as a filter). The nodes were pie-tagged according to the sample type (blank, waste blanck, withania extract) and specific shape (triangle MS1 dereplication), color (GNPS), and a border color (Moldiscovery) were given. When two annotation tools (for eg. In silico + experimental) were coincidental, the resulting data were disclosed in the data csv file using different levels of annotation confidence.

Team 2. For the untargeted metabolomics analysis, raw LC-Orbitrap-HRMS DDA data in positive mode were directly imported to the MZMine 2.53 software [11]. For mass detection, noise level was set at 5.0E5 and Rt range was set to 0-11 min. The ADAP chromatogram builder [12] was employed to connect the generated mass list. Minimum group size was set at 5, group intensity threshold and minimum highest intensity at 5.0E5, while *m/z* tolerance was set at 0.001 Da or 5 ppm. Chromatogram deconvolution was applied employing the baseline cut-off algorithm with the minimum peak height set at 8.0E5,

and baseline level was set at 5.0E5, with the peak duration set to maximum 0.5 min. Deconvoluted chromatograms were then deisotoped with an Rt tolerance of 0.2 min and a maximum charge of 2, followed by alignment using the Join aligner module. For alignment, Rt tolerance was set at 0.05 min, with the weight being 20 and 10 for m/z and Rt respectively. Filtering was performed with the "Feature list rows filter", to include only features present in at least 3 samples. Finally, the peak list was manually processed so as to eliminate features appearing also in procedural and waste blank samples and peaks with extensive tailing. The final peak list comprised of 225 features, and it was transferred to an excel sheet. For metabolite annotations, prior to any hands-on analysis, and since this was our first contact with the plant *Withania somnifera*, an examination of the available literature was deemed necessary. At first, our search was directed to reviews and phytochemical analysis articles dealing with this species. A quick overview of the literature revealed that extracts of this plant (and particularly the roots), are rich in withanolides (steroidal lactones comprising of 28 carbon atoms) and their glycosylated forms, withanosides. The latter information would prove quite valuable later in limiting the putative chemical formulas assigned to each *m/z* feature. The features of the generated peak list were manually examined one by one against the .raw spectra using the Xcalibur software (version 2.2, Thermo Scientific) which is available in our lab. By making use of the "Elemental composition" tab the formula, RDBeq. and delta (ppm) were recorded for each feature. HRMS/MS data were also assigned to each feature. We hereby have to note that despite the fact that for this exercise the datafile Orbi_P_E2_WS03A was examined, for a real metabolomics dataset, this process would normally be performed for the QC pooled sample(s).

Even though an effort was made to match detected features with entries of available online mass spectrometry databases (eg. Metlin [20], Massbank [21], HMDB [22]), contrary to other, more widespread compound groups such as flavonoids, withanolides were severely underrepresented. Therefore, compound annotation was largely based on information collected from published articles. This time our search included the more specific terms "withanolides", "LC-MS", "fragmentation" and "mass spectrometry". The papers published by Musharraf et al. [23], Tetali et al. [24], Trivedi et al. [25], Girme et al. [26] and Khajuria et al. [27], were amongst the ones that stood out as the most helpful for this particular dataset. In fact, the extensive and comprehensive withanolide fragmentation patterns proposed by Musharraf et al. [23]were of particular importance in our annotation effort (Fig. SI**). Additionally and in accordance with the aforementioned literature, it appears that for most withanolides, the most intense ion corresponds to the $[M+NH_4]^+$ adduct, while other adducts and dehydration products were also present in the HRMS spectrum. Finally, features that corresponded to adducts or fragments of other compounds were removed from the original peak list.

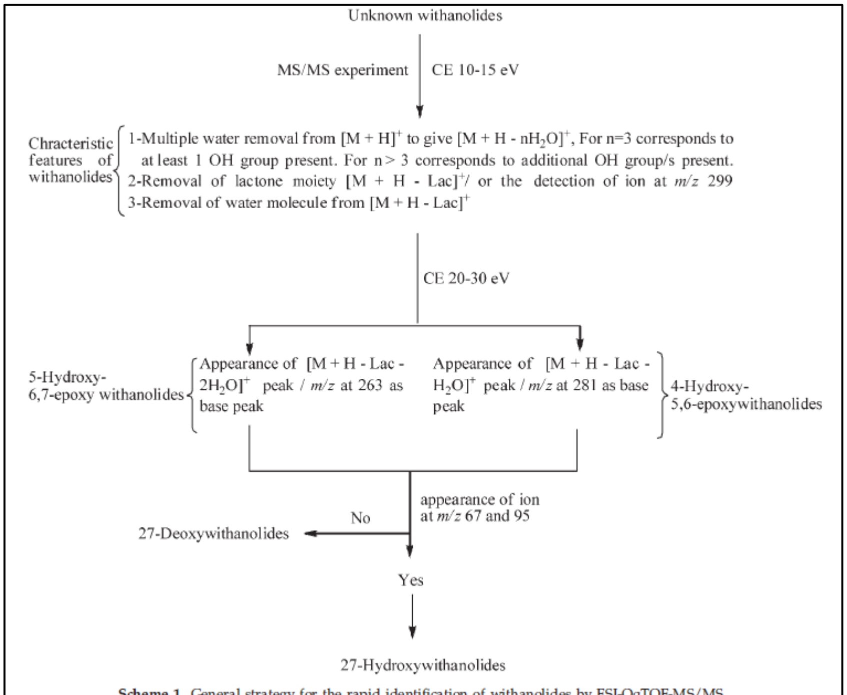

Scheme 1. General strategy for the rapid identification of withanolides by ESI-QqTOF-MS/MS.

Figure A. Withanolide fragmentation patterns suggested by Musharraf et al. [23]

Team 3. Feature Finding: The *W. somnifera* Orbi_POS_DDA (positive mode, data dependent analysis – MS2) RAW MS data were downloaded and used as inputs. A workflow in Compound Discoverer 3.3.0.550 was created in which the following nodes and parameters were used to process the MS data.

1. Input Files

2. Select Spectra

Spectrum Properties Filter

Lower RT Limits = 0; Upper RT Limit = 0; First Scan= 0; Last Scan = 0; Ignore Specified Scans = null; Lowest Charge State = 0; Highest Charge State = 0; Minimum Precursor Mass = 100 Da; Maximum Precursor Mass = 1000 Da; Total Intensity Threshold = 0; Minimum Peak Count = 1

Scan Event Filters

Mass Analyzer = Any; MS Order = Any; Activation Type = Any; Minumum Collision Energy = 0; Maximum Collision Energy = 1000; Scan Type = Any; Polarity Mode = Any; MS1 Mass Range = null; FAIMS CV = null

Peak Filters

S/N Threshold (FT=only) = 1.5

Replacement for Unrecognized Properties

Unrecognized Charge Replacements = 1; Unrecognized Mass Analyzer Replacements = ITMS; Unrecognized MS Order Replacements = MS2; Unrecognized Activation Type Replacements = CID; Unrecognized Polarity Replacements = +; Unrecognized MS Resolution@ 200 Replacement = 60000; Unrecognized MSn Resolution@200 Replacement = 30000

General Settings

Precursor Selection = Use MS(n-1) Precursor; Use Isotope Pattern in Precursor Reevaluation = True; Provide Profile Spectra = Automatic; Store Chromatograms = False

3a. Align Retention Times

General Settings

Alignment Model = Adaptive curve; Alignment Fallback = Use Linear Model; Maximum Shift [min] = 0.5; Shift Reference File = True; Mass Tolerance = 5 ppm; Remove Outlier = True

3b. Export Spectra

Output Data

Export Format = Mascot Generic Format (*.mgf)

4. Detect Compounds

General Settings

Mass Tolerance [ppm] = 5; Minimum Peak Intensity = 10000; Minimum # Scans per Peak = 5; Use Most Intense Isotope Only = True

Trace Detection

Maximum Number of Gaps to Correct = 2; Minimum Number of Adjacent Non-Zeros = 2

Peak Detection

Chromatographic S/N Threshold = 1.5; Remove Baseline = False; Gap Ratio Threshold = 0.35; Maximum Peak Width [min] = 1.0; Minimum Relative Valley Depth = 0.1

Isotope Pattern Detection

Group Isotopes for = Br and Cl; Use Peak Quality for Isotope Grouping = True; Filter out Features with Bad Peaks Only = True; Zig-Zag Index Threshold= 0.2; Jaggedness Threshold = 0.4; Modality Threshold = 0.9; Remove Potentially False Positive Isotopes = True

Compound Detection

Ions = [M+H]+1; [M+H-H2O]+1; [M+H-NH3]+1; [M+Na]+1; [M+NH4]+1; [M-H]-1; [M-H+HAc]-1; [M-H-H2O]-1; Base Ions = [M+H]+1; [M-H]-1; Remove Singlets = True

AcquireX Settings

Detect Persistent Background Ions = False

5. Group Compounds

General Settings

Mass Tolerance = 5 ppm; RT Tolerance [min] =0.25; Align Peaks = False; Preferred Ions = [M+H]+1; [M-H]-1; Area Integration = Most Common Ion

Peak Rating Contributions

Area Contribution = 3; CV Contribution = 10; FWHM to Base Contribution = 5; Jaggedness Contribution = 5; Modality Contribution = 5; Zig-Zag Index Contribution = 5

Peak Rating Filter

Peak Rating Threshold = 0; Number of Files = 0

6a. Fill Gaps

General Settings

Mass Tolerance = 5 ppm; S/N Threshold = 1.5; Use Real Peak Detection = True

6b. Search mzVault

Search Settings

mzVault Library = in-house database, NIST2020, GNPS (access 03-07-2022), mzCloud Offline for Endogenous 2020B; Compound Classes = All; Match Ion Activation Type = False; Match Ion Activation Energy = Any; Ion Activation Energy Tolerance = 20; Match Ionization Method = False; Apply Intensity Threshold = True; Remove Precursor Ion = True; Precursor Mass Tolerance = 5 ppm; FT fragment Mass Tolerance = 10 ppm; IT Fragment Mass Tolerance = 0.4 Da; Match Analyzer Type = False; Search Algorithm = HighChem HighRes; Match Factor Threshold = 50; Maximum Number Results = 10; RT Tolerance [min] = 0.5; User Retention Time = False

6c. Search Mass List

Search Settings

Mass Lists = in-house mass list; Use Retention Time = True; RT Tolerance [min] = 0.15; Mass Tolerance = 5ppm

6d. Map to Metabolika Pathways

Search Settings

Metabolika Pathways = all included in CD 3.3.0.550; Search Mode = By Formula or Mass

By Mass Search Settings

Mass Tolerance = 5 ppm

By Formula Search Settings

Maximum Number of Predicted Compositions to be searched = 3

Display Settings

Maximum Number of Pathways in 'Pathways' column = 20

6e. Predict Compositions

Prediction Settings

Mass Tolerance = 5 ppm; Minimum Element Counts = C,H; Maximum Element Counts = C90 H190 Br3 Cl4 K2 N10 Na2 O18 P3 S5; Minimum RDBE = 0; Maximum RDBE = 40, Minimum H/C = 0.1, Maximum H/C = 4; Maximum Number of Candidates = 10; Maximum Number of Internal Candidates = 200

Pattern Matching

Intensity Tolerance [%] = 30; Intensity Threshold [%] = 0.1; S/N Threshold = 3; Minimum Spectral Fit [%] =30; Minimum Pattern Coverage [%] = 90; Use Dynamic recalibration = True

Fragments Matching

Use Fragments Matching = True; Mass Tolerance = 5 ppm; S/N Threshold = 3

6f. Assign Compound Annotations

General Settings

Mass Tolerance = 5ppm

Data Sources

Data Source #1 = MassList Search; Data Source #2 = mzVault Search; Data Source #3 = Predicted Compositions

Scoring Rules

Use mzLogic = True; Use Spectral Distance = True; SFit Threshold = 20; SFit Range =20

Reprocessing

Clear Names = False

7. Mark Background Compounds

General Settings

Maximum Sample/Blank = 3; Maximum Blank/Sample = 0; Hide Background = True

Post-processing of Annotations: The "compounds" table was exported as an Excel file, sorted by annotation, and all unannotated features were removed. Further, features were removed that had relative standard deviation values greater than 20% (calculated from n = 3 files). InChI and InChI Key were generated using the PubChem Identifier Exchange Service using a synonym search and subsequent review of returned values. InChI values were used to search the LOTUS resource [28] (https://lotus.naturalproducts.net/) to report the chemical family at the Superclass level of taxonomy. Molecular formulae were obtained from LOTUS or PubChem via InChI. Exact masses were computed using the molecular formulae via Molecular Mass Calculator (https://www.lfd.uci.edu/~gohlke/molmass/). Annotation levels were based on MS/MS spectral library matching to in-house and public databases, a match is indicated as a level 2 annotation; however, in many cases, the matches are likely isomers of the annotated chemical. Without the ability to distinguish which annotations are proper and which ones are isomeric, we report all MS/MS matches as level 2. Annotations based only on accurate mass (matching a non-comprehensive, in-house mass list) are indicated as level 5.

Team 4. The positive ion mode DDA Orbitrap raw dataset was processed using Progenesis QI™ software with METLIN™ MS/MS Library 2019 plugin V1.0.7642.33805 (NonLinear Dynamics, Newcastle Upon Tyne, UK) and entailed peak picking, deconvolution, alignment and searching three databases to assist in compound annotations as previously described [29]. A minimum abundance threshold of 5000 was established. The retention time window from 0.3 to 8.5 min was considered. The automatic sensitivity was set at 3 (intermediate), and the peaks were deconvoluted for M+H, M+H-H2O, M+Na, and M+ACN+H. All molecular features consistently detected in the blanks were removed from further processing. Consistently with L2 annotations, molecular features were considered only if their corresponding MS/MS fragments were present. Only compounds consistently directed (CV<25) in the technical triplicates were considered for annotation (this is usually performed on a pool of all the samples "QC"). For the current test, we searched the mass spectral data against METLIN [20], MassBank of North America (http://massbank.us/; experimental LC-MS/MS positive mode) and an *in-house* compound library [29]. Progenesis QI™ provides a score for the quality of the compound annotation, using a range from 0 to 100, with 100 being a perfect match based on the mean of multiple similarity metrics (i.e., exact mass, isotopic pattern, MS/MS data, CCS values, and retention time of authentic standards analyzed under the same conditions) [29]. The current dataset was annotated based on the accurate mass, isotope similarity, and experimental fragmentation score (ranging from 0 to 60, representing how well the observed data matches the spectral library entries). A Progenesis QI score ≥ 50 is typically reached when isotopic pattern similarity is above 90%, experimental MS/MS spectral data similarity is > 50%, and the deviation of the accurate mass from the exact mass is lower than 10 ppm. For the DDA Orbitrap raw data, a Progenesis QI score ≥ 50 was considered as adequate for being considered as a candidate for putative annotation (L2 annotations according to Sumner *et al.* [30]). Only level 2 annotations were considered. The reported feature list was exported in csv format and processed in excel 2016 to harmonize the required format of this partnership.

Team 5. The Orbitrap positive DDA RAW MS data were imported in MS-DIAL 4.80 [31]. The deconvolution parameters were set for mass signal extraction between 100 and 1600 Da from 0.5 to 11 min and 50 to 1500 Da for MS2. Tolerance was set to 0.01 and 0.05 Da in centroid mode for MS1 and MS2 respectively. Elements such as "Br" and "Cl" were retained for the analysis. The detection threshold was set to 1.0×10^5 concerning MS1 and 10 for MS2. Peaks were aligned on an extract sample file with a retention time tolerance of 0.15 min and a mass tolerance of 0.025 Da. The adduct detection was based on $[M+H]^+$, $[M+Na]^+$, $[M+K]^+$, $[M+H-H2O]^+$, $[M+H-2H2O]^+$, $[M+2Na-H]^+$, $[M+2K-H]^+$, $[2M+H]^+$, $[2M+Na]^+$, $[2M+K]^+$, $[M-C6H10O4-H]^+$, $[M-C6H10O5-H]^+$, $[M-C6H8O6-H]^+$. The identification tab was used by importing .MSP files gathered from GNPS, MoNa, MassBank, RICKEN and internal standard library from Metatoul-AgromiX platform for annotation level 2.

Deconvoluted data were then cleaned with the MS-CleanR workflow [14] for feature clustering and selection. Only features with MS/MS fragments were retained. The parameters were as follow: by selecting a minimum blank ratio set to 0.8, a maximum relative standard deviation (RSD) set to 45, and a relative mass defect (RMD) ranging from 50 to 3 000. The maximum mass difference for feature relationships detection was set to 0.005 Da, and the maximum RT difference to 0.025 min. One peak was kept in each cluster based on most intense and most connected nodes.

The kept features were annotated with MS-FINDER version 3.52 [32] using in silico fragmenter for annotation level 3. The MS1 and MS2 tolerances were respectively set to 5 and 15 ppm. Formula finder were exclusively processed with C, H, O, N, S, Cl atoms. Databases (DBs) based on *Withania* (genus), Solanaceae (family), were constituted with the dictionary of natural products (DNP, CRC press, DNP on DVD v. 28.2) and COCONUT. The internal generic DBs from MS-FINDER used were KNApSAcK, PlantCyc, HMDB, LipidMaps, NANPDB, and UNPD. Annotation prioritization was done by ranking *Withania* DB, followed by Solanaceae DB, and finally generic DBs using the final MS-CleanR step.

Next, the feature list generated by MS-CleanR workflow was processed in Knime v4.4.2. for NPClassifier and Classyfire ontology requesting and final data column formating, according to the provided specification.

Team 6. The Orbitrap POS and NEG DDA mzML files were downloaded and processed through MZmine 2.39. [11] MZmine parameters for the negative mode data were the following: Mass detection: MS1 = 1.0E4, MS2 = 0.0 ; ADAP chromatogram builder: Min group size = 5, Group intensity threshold = 1.0E4, min highest intensity = 1.0E4, m/z tolerance = 15 ppm; Chromatogram deconvolution: m/z range for MS2 scan pairing = 0.002 Da, RT range for MS2 scan pairing = 0.3 min, Algorithm = Wavelets (ADAP), S/N threshold = 50, S/N estimator = Intensity window SN, Min feature height = 10000, coefficient/area threshold = 150, Peak duration range = 0.01-10.00, RT wavelet range = 0.01-0.30; Isotopic peaks grouper: m/z tolerance = 0.002 m/z or 15 ppm, retention time tolerance = 0.3 min, Maximum charge = 4, representative isotope = Lowest m/z; Join aligner: m/z tolerance = 0.002 m/z or 15 ppm, Weight for m/z = 1, retention time tolerance = 0.4 min, weight for RT = 1; Peak finder: Intensity tolerance = 0.002 m/z or 15 ppm, retention time tolerance = 0.2 min, RT correction : checked; Peak filter: Height = 1.0E4-1.0E20; Feature list row filter: Keep only peaks with MS2 scan = checked; Duplicate peak filter: Filter mode = NEW AVERAGE, m/z tolerance = 0.002 m/z or 15 ppm, RT tolerance = 0.3 min; Feature list rows filter: Keep inly peaks with MS2 scan = checked, Reset the peak number ID = checked; Export/Submit to GNPS-FBMN: Merge MS/MS : Select spectra to merge = across samples, m/z merge mode = weighted average, intensity merge mode = sum intensities, Expected mass deviation = 0.002 m/z or 15 ppm, Cosine threshold = 60%, peak count threshold = 40%, isolation windows offset = 0.0 m/z, Isolation window width) 3 m/z. The parameters were the same for positive mode, except for the following: Mass detection MS2 = 5.0E5; ADAP chromatogram builder: Group intensity threshold and Min highest intensity = 5.0E5; Chromatogram deconvolution: min feature height = 500000; Peak filter: Height = 5.0E5-1.0E20.

Output files were then processed using MolNotator [33] with the following parameters : df_rt_error = True, fn_matched_peaks = 3, fn_score_threshold = 0.1, fn_mass_error = 0.001, fn_rt_error= 10, an_mass_error= 0.002, an_prec_mass_error= 0.1, an_rt_error= 10, an_cos_threshold= 0.2, an_hardcos_threshold= 0.6, an_run_bnr= True, an_bnr_neg= ["[M-H]-", "[2M-H]-"],

an_bnr_pos= ["[M+H]+", "[M+Na]+", "[M+NH4]+", "[2M+H]+", "[2M+Na]+", "[2M+NH4]+"], mm_mass_error= 0.003, mm_prec_mass_error= 0.1, mm_rt_error= 15, mm_bnr_neg= ["[M-H]-", "[2M-H]-"], mm_bnr_pos= ["[M+H]+", "[M+Na]+", "[M+NH4]+", "[2M+H]+", "[2M+Na]+", "[2M+NH4]+"], mu_skip= True, c_mass_error= 0.002, c_lowcos_threshold= 0.2, c_hardcos_threshold= 0.6, c_matched_peaks= 2, c_purge_empty_spectra= True, c_export_samples= True, mn_mass_error= 0.002, mn_cosine_threshold= 0.98, mn_matched_peaks= 8.

Further, the features were annotated using MS2Query [34]. MS2Query is not yet published, but the code can be found on Github: iomega/ms2query: MS2Query - machine learning assisted library querying of MS/MS spectra (github.com). MS2Query uses a random forest model to combine the results of MS2Deepscore [35], Spec2Vec [36], and mass difference to rank candidate molecules, by creating an ensemble score. The library used was the GNPS [17] library downloaded on 15-12-2021, using the positive ionization mode. MS2Query searches for both exact matches and analogues (not identical mass) in the library. The highest scoring library spectra was selected for each feature if the predicted ensemble score was above 0.6. Found exact matches were marked as identification level 2.

Team 7. The proposed automated procedure consisted of a complete script based on Bertrand, Guitton and Roullier [37] and evaluated during CASMI 2017 [38]. It was written in R 4.1 [39] with the XCMS [40], MSnbase [41], CAMERA [42], IPO [43], MeHaloCoA [44], RMassBank [45], Taxize [46], Rdisop [47], WikidataR [48], wikitaxa [49] packages. The complete workflow is accessible at https://chromannot.univ-nantes.fr/.

Automatic peak detection: The peak detection from each LC-HRMS raw files were achieved using XCMS [40] with appropriate parameters (no MS/MS data were used during the peak picking process). In most cases optimum parameters were not defined manually but selected using the Isotopologue Parameter Optimisation (IPO) [43] separately on each raw data file of interest. The two parameters *ppm* and *mzdiff* used by XCMS were systematically optimized. After peak detection, MS/MS data were automatically retrieved from the raw data using MSnbase [41].

Spectral interpretation: The ions detected by XCMS [40] were grouped using CAMERA [42] based on retention time similarities yielding MS pseudospectra (pcgroups). Each pseudospectrum was interpreted using CAMERA (with an in-house extended adduct list) yielding to the detection of isotopes, adducts and neutral losses.

Molecular formula determination: For each ion (except isotopes), a list of possible molecular formulae (MFs) was deduced by SIRIUS3 [47] based on MS and MS/MS spectra, using $C_xH_yN_{10}O_{28}P_2$ as maximum possible atoms based on existing MFs in the Dictionary of Natural Products (DNP) [50] as reported by Kind and Fiehn [51]. The maximum number of carbon (x) and hydrogen (y) was estimated based on the detected *m/z*; x was set to (*m/z*)/12 and y to (*m/z*)/2. The potassium and sodium atoms were also added only in the case of $[M+K]^+$ and $[M+Na]^+$ adducts occurrence (predicted by CAMERA), respectively. When no adduct information were detected during the spectral interpretation step, only $[M+H]^+$, $[M+Na]^+$, $[M+K]^+$, $[M]^+$ were considered in positive ionisation and only $[M-H]^-$, $[M+HCOOH-H]^-$ in negative ionisation. In addition, S, Cl and Br were automatically detected from the isotopic patterns using a script adapted from MeHaloCoA [44] to reduce calculation time when those atoms may be present [52]. Finally, the SIRIUS3 score (S_{SIRIUS}) was used to discriminate between possible MF. Compound MF was then deduced by adduct correction.

When multiple adducts were detected in an MS spectrum, yielding some MF to be detected for multiple times in one spectrum (after adduct correction), the S_{SIRIUS} was corrected based on MF redundancy score (S_{red}) between all adducts [53]. This correction was based on the addition to the S_{SIRIUS} score of the S_{red} calculated as follows: 10 times the number of occurrences of the MF among all adducts over the maximum number of occurrences of a MF among all proposed MF of a given challenge.

Structure determination by database search: Each of the compound possible MFs was searched within various online and local DBs for matches. A large number of DBs were used, listed in Wolfender, Nuzillard, van der Hooft, Renault and Bertrand [54]. The search was performed in April 2022 in Wikidata (LOTUS - https://lotus.naturalproducts.net/) using WikidataR [48], wikitaxa [49] and in GNPS [55]. From all these DBs, as much information as possible was retrieved (such as InChI, SMILES, MOL file, metadata). During this process missing structural information were possibly obtained from conversion tools: OpenBabel [56].

Biological source similarity scoring: For all proposed structures, when biological sources were reported in the databases, similarity between phylogeny of the compounds, and the phylogeny of ashwagandha (*Withania somnifera*). Such comparison was performed using Taxize [46]. Scores S_{Phylo} were defined as follow: no similarity -10, same kingdom 0, same phylum 5, same class 6, same order 7, same family 8, same genus 9 and same species 10.

MS/MS similarity scoring: To further discriminate between all proposed structures for a given peak, a strategy based on reported and *in silico* fragmentation was performed. When no reported MS/MS spectra exists, simulation were performed by competitive fragmentation modelling using CFM-ID 4.0 [57]. Finally, all spectra were compared with the acquired one using TREMOLO [58], yielding “cosin scores”. Final scores, based on spectral DB (S_{MS^2DB}) and/or CFMID fragmentation (S_{MS^2CFMID}), were 50 times the “cosin scores”.

Data reporting: Finally, for all extracts, annotations with consistent molecular formula ($S_{SIRIUS} + S_{red} > 0$) were provided only if they possess consistent phylogeny ($S_{Phylo} > 7$ – same order of higher) or MS^2 compared to reported spectra ($S_{MS^2DB} \geq 30$ – “cosin scores” higher that 6) or MS^2 compared to *in silico* spectra ($S_{MS^2CFMID} \geq 20$) only if S_Phylo ≥ 0.

Team 8

MZmine Feature Detection and Reduction. An Intel® Core™ i9-10900 CPU (2.80 GHz) with 64.0 GB RAM workstation was employed for data processing. The MS data (as .mzML files) were loaded into MZmine 2.53. [11] The following modules

and parameters were employed for the generation of feature lists from the MS data: The mass detection step kept the ions above a noise level set at 5×10^3 (and 0 for MS/MS) (Table 1). The Automated Data Analysis Pipeline (ADAP) chromatogram builder [12] was employed with minimum group size of 5 scans (according to [3]), an *m/z* tolerance of 0.003 Da, and group intensity thresholds **of** 5×10^3, and a minimum highest intensity of 1.5×10^4. The chromatogram deconvolution was performed using the Local Minimum Search algorithm with a chromatographic threshold of 80%. The search minimum in RT range was 0.06 min, the minimum relative height was 0%, the minimum absolute height was 1.5×10^4, the min ratio of peak top/edge was 2, and the peak duration range was from 0.00 to 2.00 min. The *m/z* center calculation was set to median. The MS/MS scan pairing parameters were 0.025 Da and 0.1 min. The feature lists were deisotoped using the isotope peak grouper with an *m/z* tolerance of 0.0015, a RT tolerance of 0.05 min, a maximum charge of 3, and the representative isotope used was the most intense. Peak alignment was obtained using the "join aligner" method with an *m/z* tolerance of 0.0015 Da, an absolute RT tolerance of 0.05 min, a weight for *m/z* of 2, a weight for RT of 1, and an isotope pattern comparison of a minimum of 50%. Gap-filling was applied with the "same RT and *m/z* range gap filler" module with an *m/z* tolerance set at 0.0015 Da. After gap-filling, the "peak filter" module was employed to keep features with at least 5 scans, and a peak height equal to or above the minimum peak height set during the deconvolution to eliminate non-compliant features collected during gap-filling. The "duplicate peak filter" module was applied with an *m/z* tolerance of 0.0015 Da and an RT of 0.05 min. The "features list rows filter" module was employed to reduce the feature list to features with at least two isotopes (two-isotope filter). The GNPS Export Module was used to generate the feature list as an MGF file and as a CSV file to be used for Feature-Based Molecular Networking (FBMN) on the GNPS website. [59, 60] The SIRIUS Module was used to generate an MGF file for feature annotation using the SIRIUS 4 software. [61]

Molecular Networking and Spectral Library Search. A molecular network was generated using the online Feature-Based Molecular Networking (FBMN) workflow [60] at GNPS [59] (http://gnps.ucsd.edu) with a parent mass tolerance of 0.02 Da and an MS/MS fragment ion tolerance of 0.02 Da. A molecular network was then created where edges were filtered to have a cosine score above 0.7 and more than 6 matched peaks. Edges between two nodes were kept in the network if and only if each of the nodes appeared in each other's respective top 10 most similar nodes. The maximum size of a molecular family was set to 100, and the lowest scoring edges were removed from molecular families until the molecular family size was below this threshold. The spectra in the network were then searched against GNPS spectral libraries. [59, 62] The library spectra were filtered in the same manner as the input data. All matches kept between network spectra and library spectra were required to have a score above 0.5 and at least 6 matched peaks. The DEREPLICATOR was used to annotate MS/MS spectra. [63] The molecular networking job can be publicly accessed at https://gnps.ucsd.edu/ProteoSAFe/status.jsp?task=2acf953135534e1eb3436090afa97e03.

SIRIUS Annotation. The MGF file exported from MZmine was imported into SIRIUS software (v5.5.7) [61] for SIRIUS [47, 64] molecular formula prediction, ZODIAC [65] molecular formula ranking, CSI:FingerID [66, 67] molecular fingerprint/structure predication, and CANOPUS [68-70] compound class annotation. The Orbitrap default parameters were used with a 5-ppm mass accuracy for MS2 spectra and the following possible adducts: ($[M + H]^+$, $[M + NH_4]^+$, $[M + Na]^+$, and $[M + K]^+$. Biological databases were used for structural matching. CANOPUS was run as default (no parameters needed). The SIRIUS adduct annotations were reviewed manually and any misannotated ions were rerun through SIRIUS, ZODIAC, CSI:FingerID, and CANOPUS with the proper adduct annotation (*e.g.*, some ions were annotated as $[M + H]^+$ when they should have been $[M + NH_4]^+$). Rerunning with proper adduct annotation improved molecular formula, structure, and class prediction results.

FBMN SIRIUS Integration using Jupyter notebook. SIRIUS annotations were mapped onto GNPS-produced molecular networks using a Jupyter Notebook available at https://github.com/mwang87/GNPS_Sirius_Integration_Notebooks. [60] The molecular networks were visualized using Cytoscape software. [71] The nodes were pie-tagged according to the sample type (blank, waste blank, *Withania*) and shape-tagged according to database structure annotations.

Feature Reduction - Blank Filtering. The feature table was exported from Cytoscape and processed in Excel 2016. Blank filtering consisted in eliminating features with a feature area ratio between *Withania* samples and blank samples (Extraction Blank and Solvent Blank each applied separately) equal to or higher than 80% (based on [14]).

Feature Annotation. The feature table was condensed–by removing extraneous details not needed for the challenge–and the features annotated using a combined manual and python script approach. *Table Generation.* The columns titled: id, inchi, family, mf, feature, rt, mz, area, and Smiles, were created and populated with the data from the following columns: Sirius:name, Sirius:InChI, Canopus:NPCsuperclass, Sirius:molecularFormula, Sirius:adduct, RTConsensus, precursor mass, GNPSGROUP:SAMPLE, and Sirius:smiles. *RDKit Annotations*. The RDKit 2022.03.3 framework (RDKit: Open-source cheminformatics; http://www.rdkit.org) was used to transform SIRIUS SMILES into molecules and obtain molecular descriptors, including molecular weight, molecular formula, InChIKey, and monoisotopic mass. The feature table was manually inspected to observe consistent values obtained from GNPS, SIRIUS, and RDKit annotations. *Putative Adduct Annotations*. To complement the SIRIUS and GNPS adduct annotations, a custom python script and manual annotation were used to annotate *m/z* and RT features with plausible adduct classifications (e.g., ($[M + H]^+$, $[M + NH_4]^+$, $[M + Na]^+$, etc.), based on mass differences between a proposed adduct and the *m/z*. A retention time tolerance of 0.02 min was used to identify multiple, putative clusters of an analyte. The GNPS, SIRIUS, and manual adduct annotations aided feature reduction during the "Analyte Generation" step 7 below.

Feature Reduction - Analyte Generation: *MS2Analyte* Analysis. The same MS data (as .mzML files) used for MZmine analysis were imported into MS2Analyte, an automated tool for MS data processing and analyte detection which is currently

under development in the Linington Lab. The program utilizes statistical correlations to group MS features (*i.e.*, ions detected by MS; *m/z* and RT pairs) into analytes (*i.e.*, a single chemical entity). One of the outputs of the program is a list of *mz* and RT features labeled with an extract analyte ID (EAID), wherein features with the same EAID make up the same analyte. The MS2Analyte output was filtered to remove analytes detected in the blanks and filtered using the relative standard deviation (RSD) among triplicates. [72] *MS2Analyte Annotation.* The features present in the combined MZmine, GNPS, and SIRIUS feature table (steps 1-6) were annotated with the EAID's from the MS2Analyte analysis by matching the *m/z* (±5.0E^{-6}) and RT (±0.02 min) values between the datasets.

Final Feature Reduction and Data Report. Any features sharing the same EAID were manually reduced to a single analyte, described by the feature with the greatest abundance (*i.e.*, area), and feature characteristics (e.g., adduct annotation and *m/z*) were combined into a notes columns (*e.g.*, 314.1393 [M+H]+|336.1223 [M+Na]+|352.0952 [M+K]+). Features sharing the same EAID represented different adducts of the molecular ion (e.g., $[M+H]^+$, $[M+NH_4]^+$, $[M+Na]^+$, etc.). Finally, the analyte list was processed in Excel 2016 to meet the required format of the collaboration, duplicate analytes (*e.g.*, same *m/z*, RT, and ID) were removed, annotation confidence levels (adapted from [73, 74]) were provided–based on complementary annotations from GNPS and SIRIUS–, and the final data table reported in .csv format.

Section 5. Strategy overview

Figure S1 to S10 summarized the strategies applied by each participant.

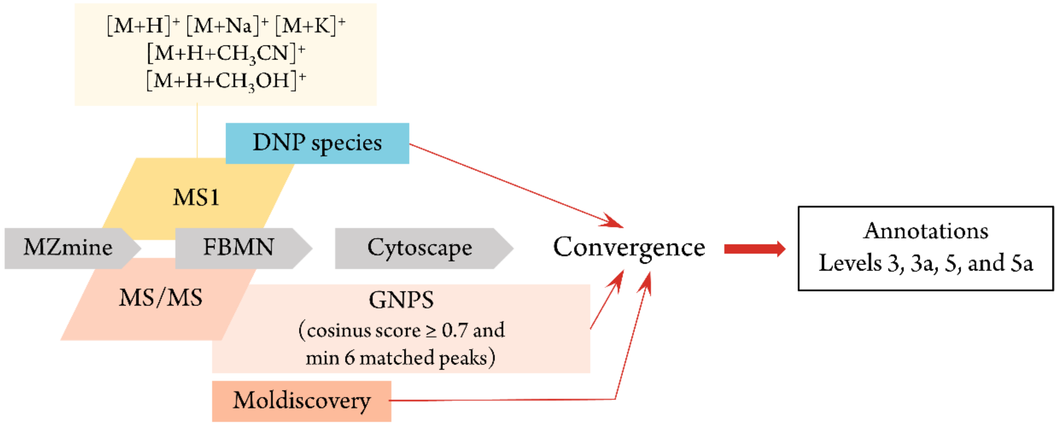

Key message

Convergence: high confidence is assigned when convergent annotations were obtained from the different tools.

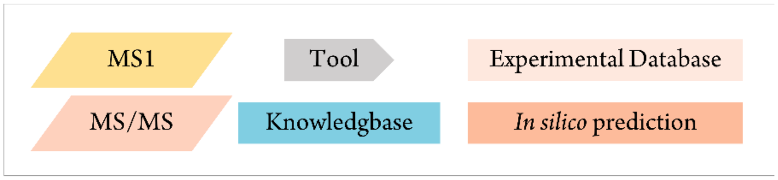

Figure S 1.
Strategy applied by team 1.

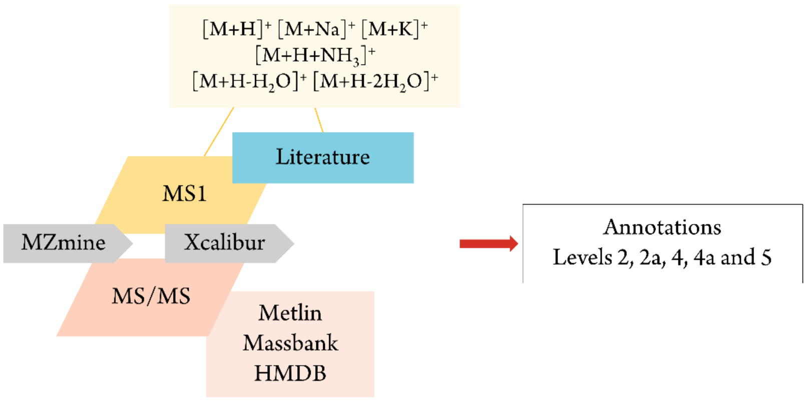

Key message

The spectral databases contain few entries of molecules described for *Withania somnifera*. Manual interpretation based on published articles is prioritized.

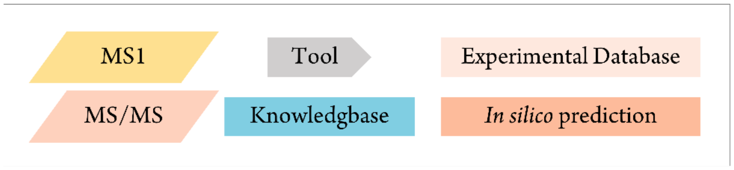

Figure S 2.
Strategy applied by team 2.

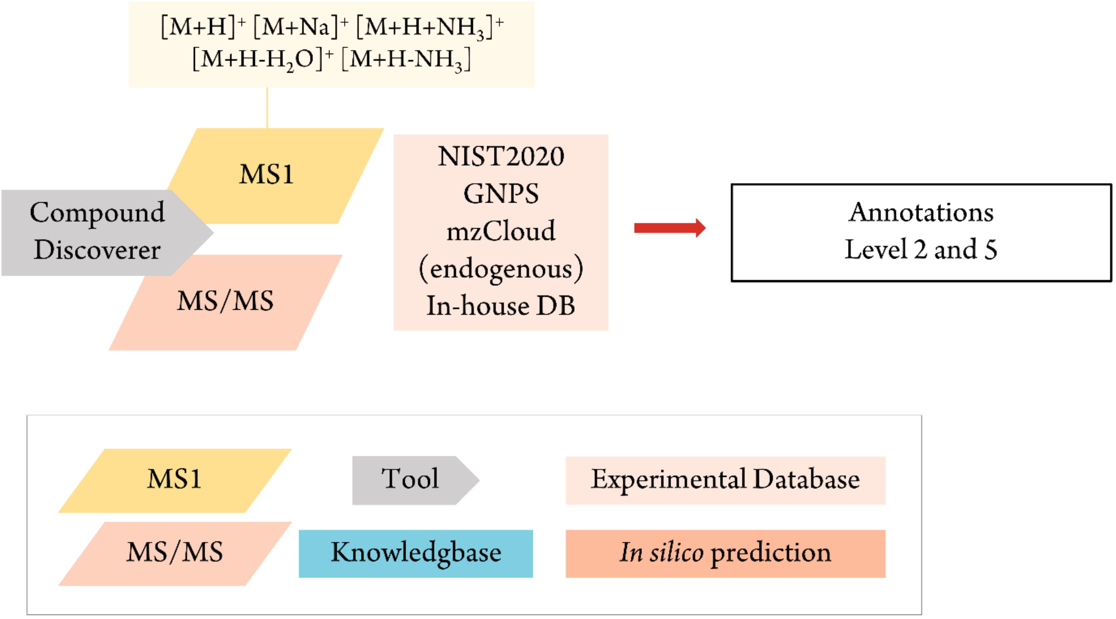

Figure S 3.
Strategy applied by team 3.

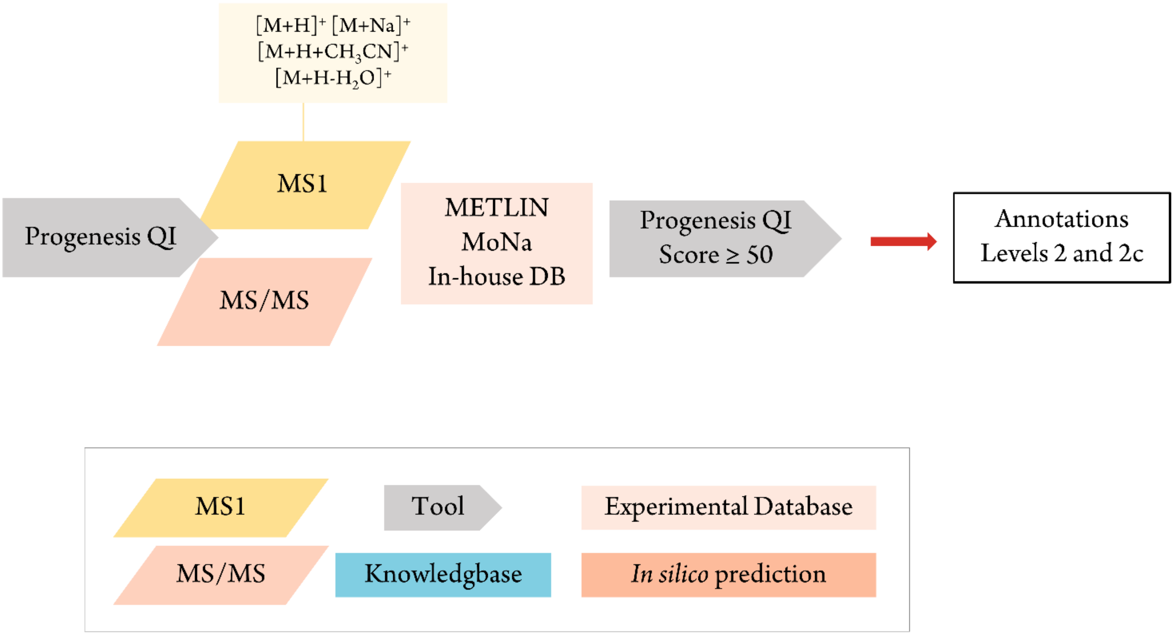

Figure S 4.
Strategy applied by team 4.

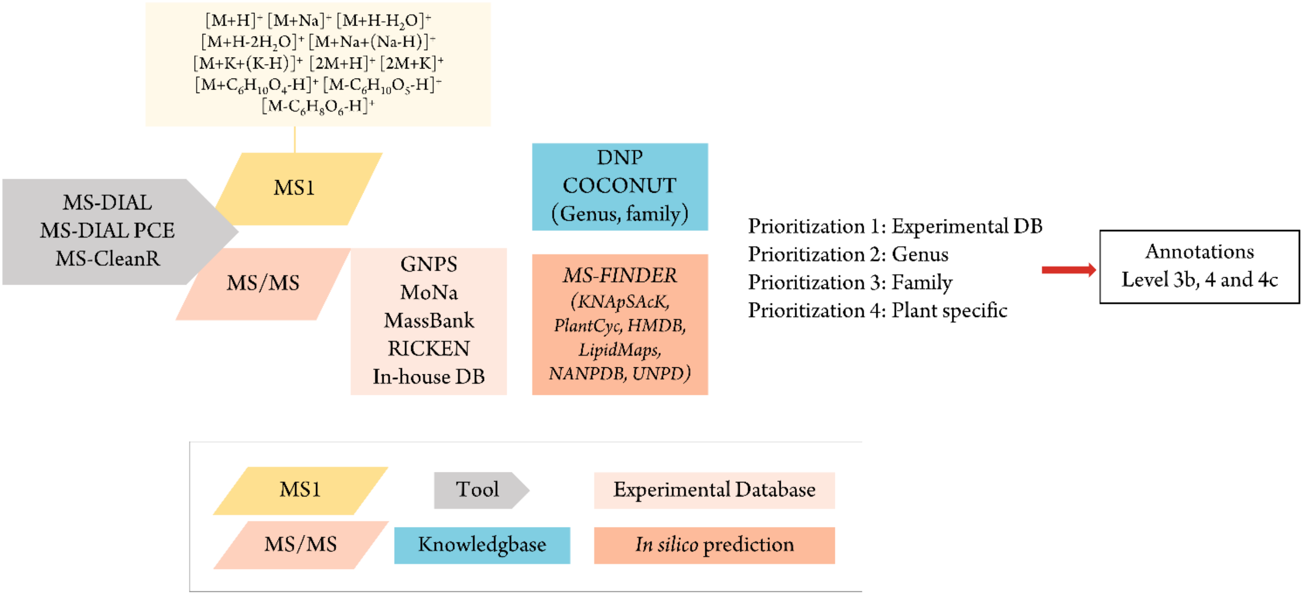

Figure S 5.
Strategy applied by team 5.

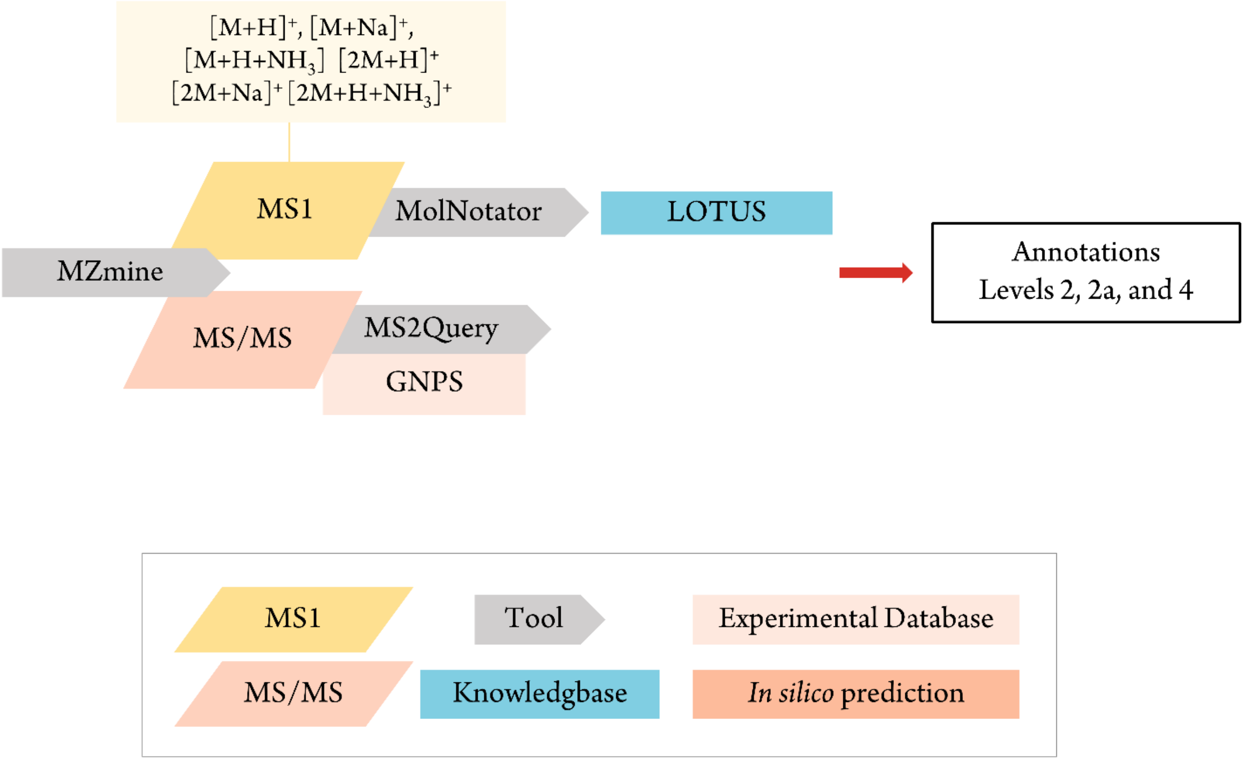

Figure S 6.
Strategy applied by team 6.

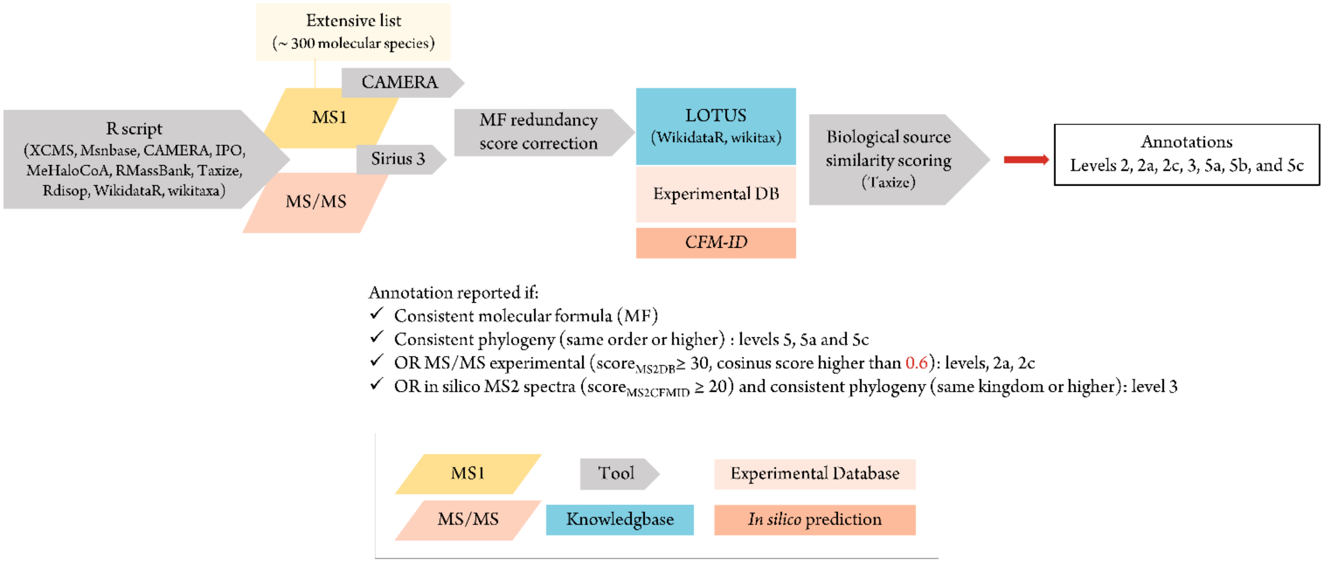

Figure S 7.
Strategy applied by team 7.

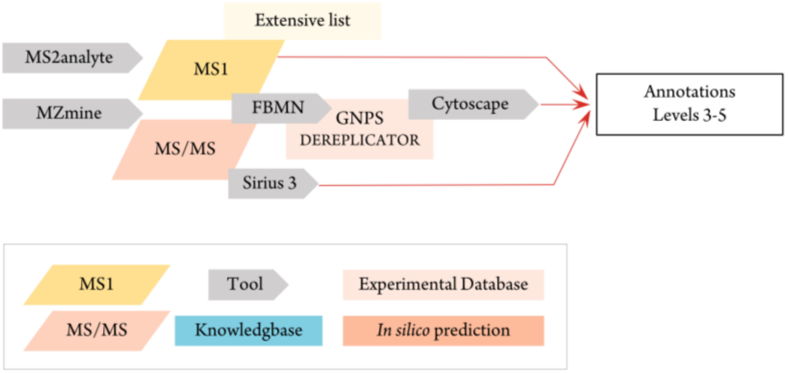

Figure S 8..
Strategy applied by team 8.

Section 6. Survey: data preprocessing

The following figures are related to the comparison of pre-processing approaches employed by the 8 teams.working with the dataset acquired on the Orbitrap in positive ionization DDA mode.

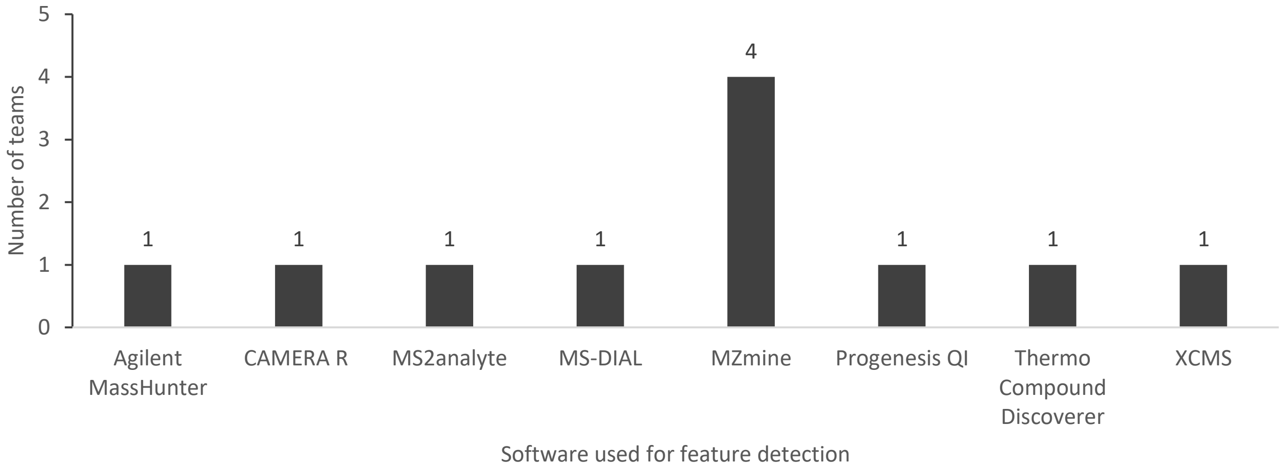

Figure S 9.
Software employed for feature detection.

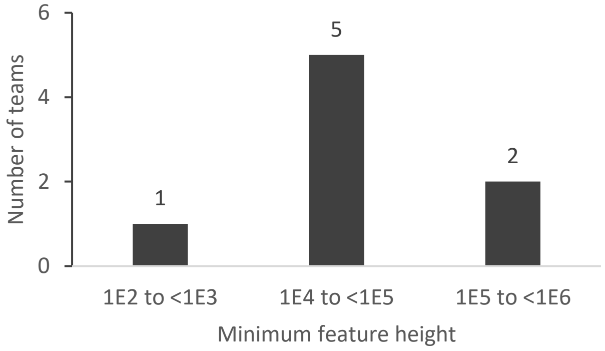

Figure S 10.
Minimum feature height intensity employed for feature detection.

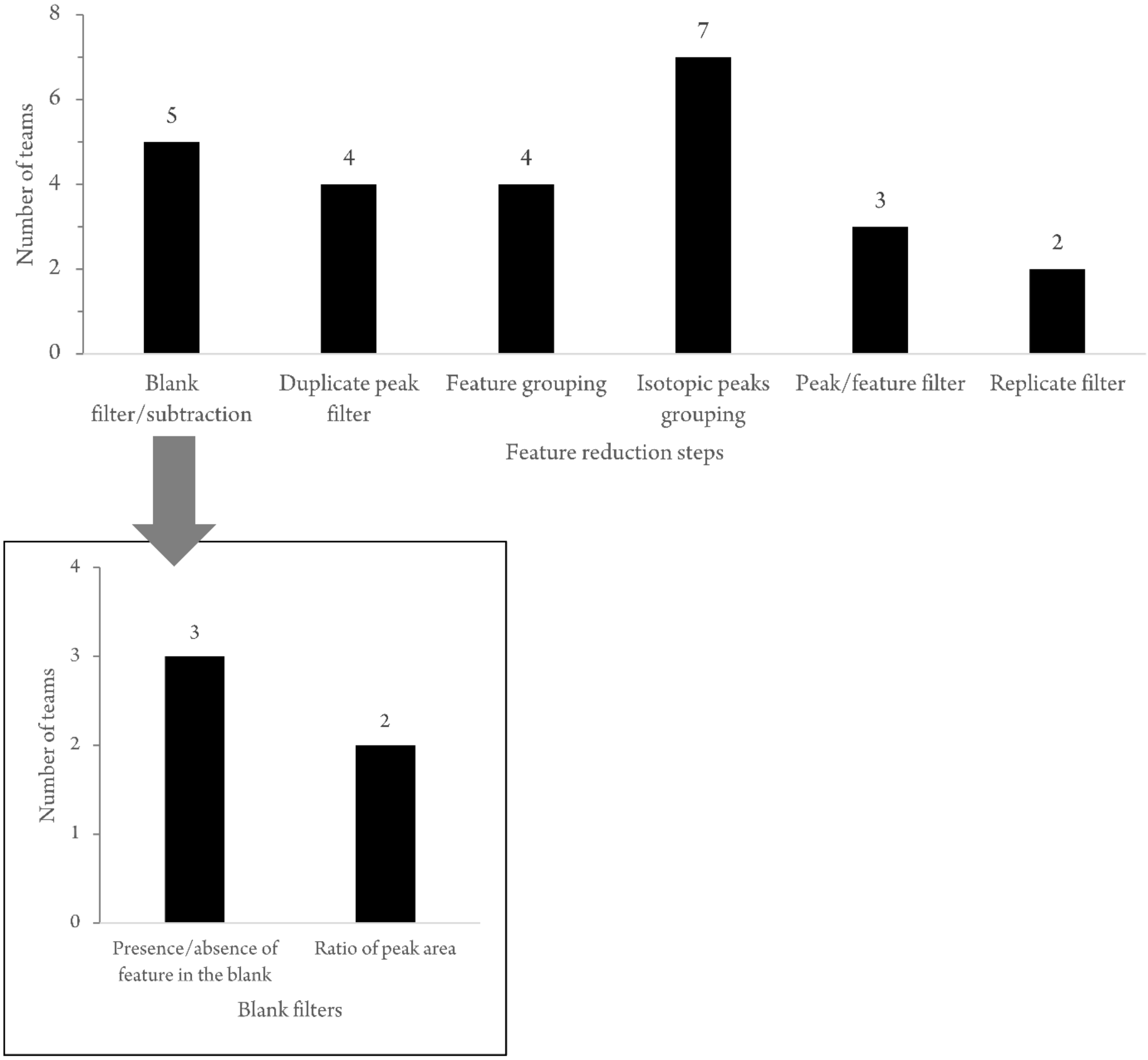

Figure S 11.
Type of feature reduction steps applied to the feature list. The different types of feature reduction steps were defined as followed: Blank filter/subtraction: removal of peaks based on presence/absence, ratio of peak area/height, etc. in blank samples. Duplicate peak filter: removal of peaks/features with the same m/z and retention time. Feature grouping: detect fragment and adduct peaks/features and group them together into a single analyte (note: This is not simply annotating the degenerate features, but actually reducing the list by grouping them). Isotopic peaks grouping: detect isotopic peaks/features and group them together into a single feature and isotopic pattern. Peak/feature filter: removal of peaks based on a height/intensity, shape, or number of data points requirement. Replicate filter: removal of peaks based on presence/absence, RSD, etc. between replicate samples

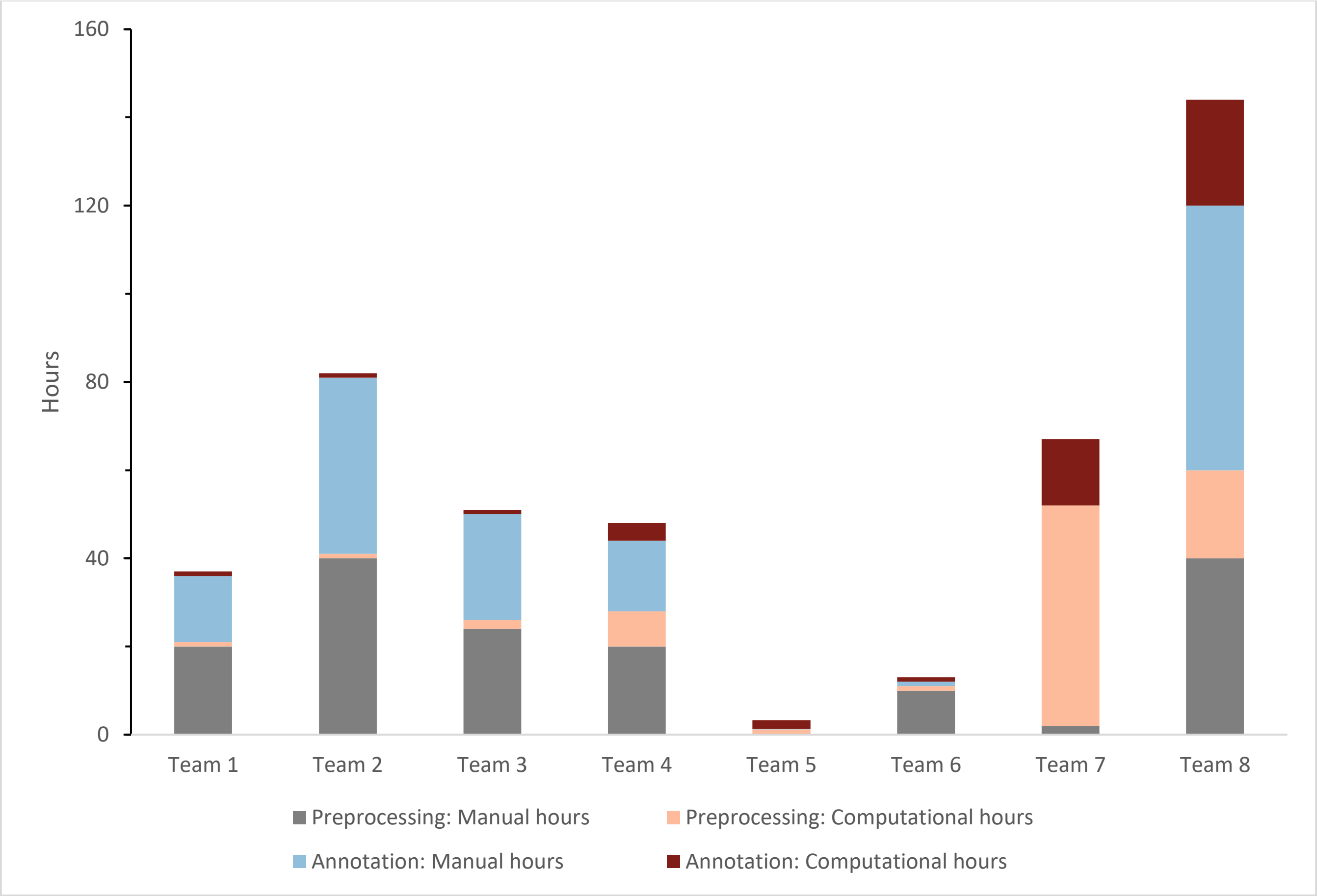

Figure S 12.
This histogram illustrates the time spent on the project by the 10 teams.

Section 7. Description of annotations for each individual team

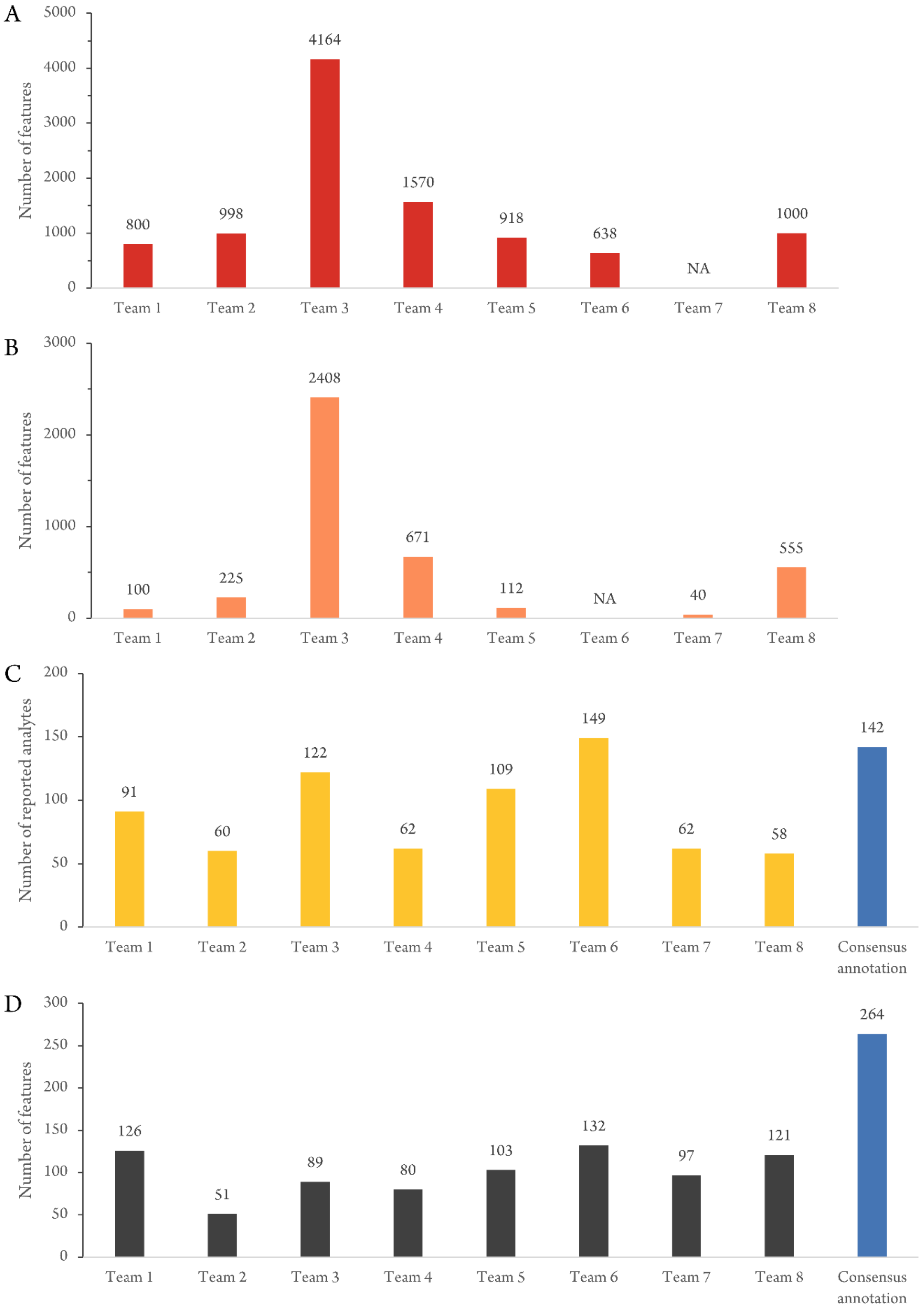

Figure S 13.
A) Number of features reported by the participants after feature detection, B) number of features reported by the participants after any filtering and reduction steps, C) number of analytes reported by the participants and final number of analytes in the consensus annotation table, D) number of features per team considered in the consensus annotation process after applying the exclusion criteria and final number of features in the consensus annotation table. "NA" indicates that no numbers were reported by the team due to the data processing strategy.

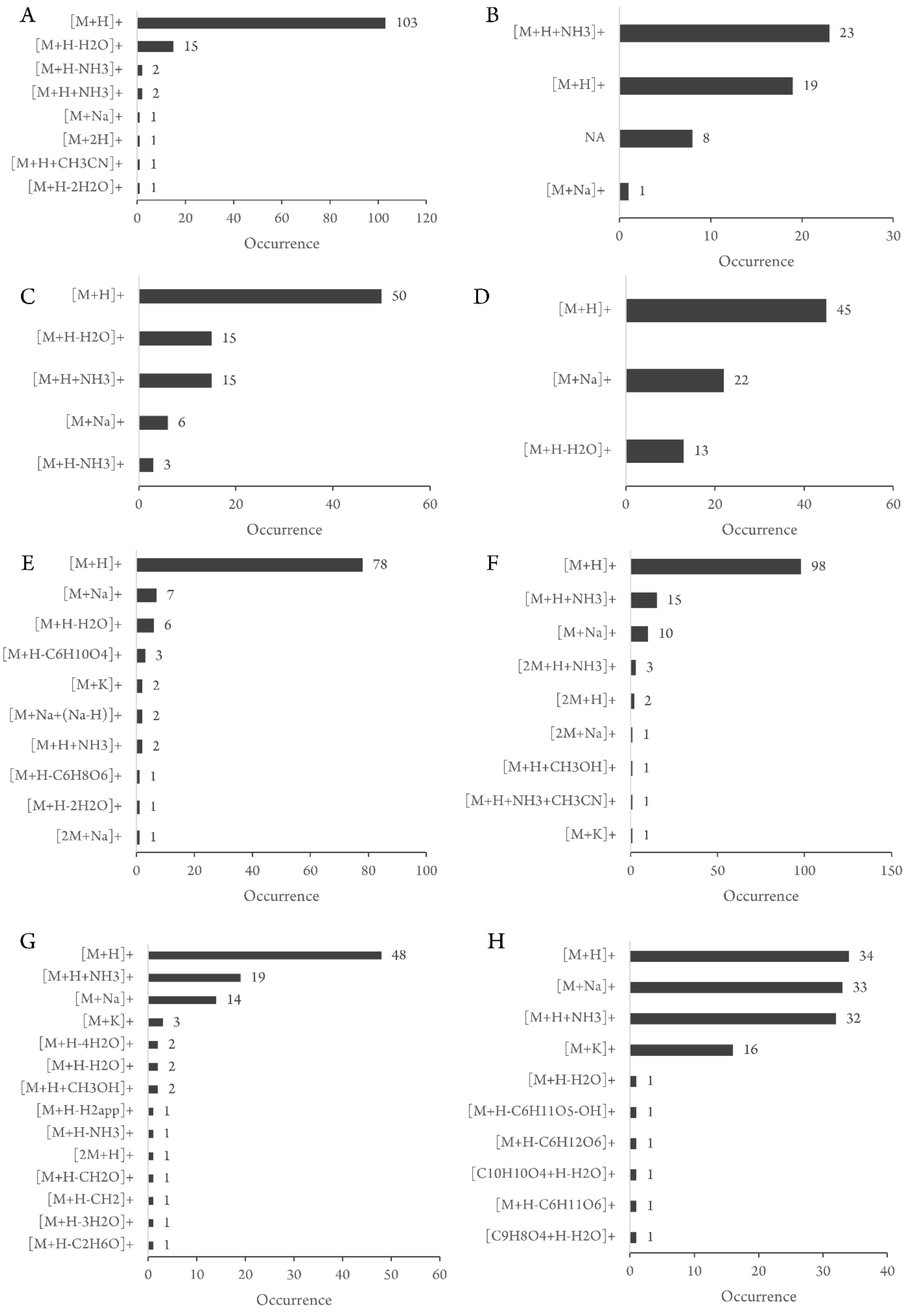

Figure S 14.
The ion species description varied from three to 14 types among participants who used the datasets acquired on the Orbitrap-DDA in positive ionization. A) to H) Team 1 to 8.

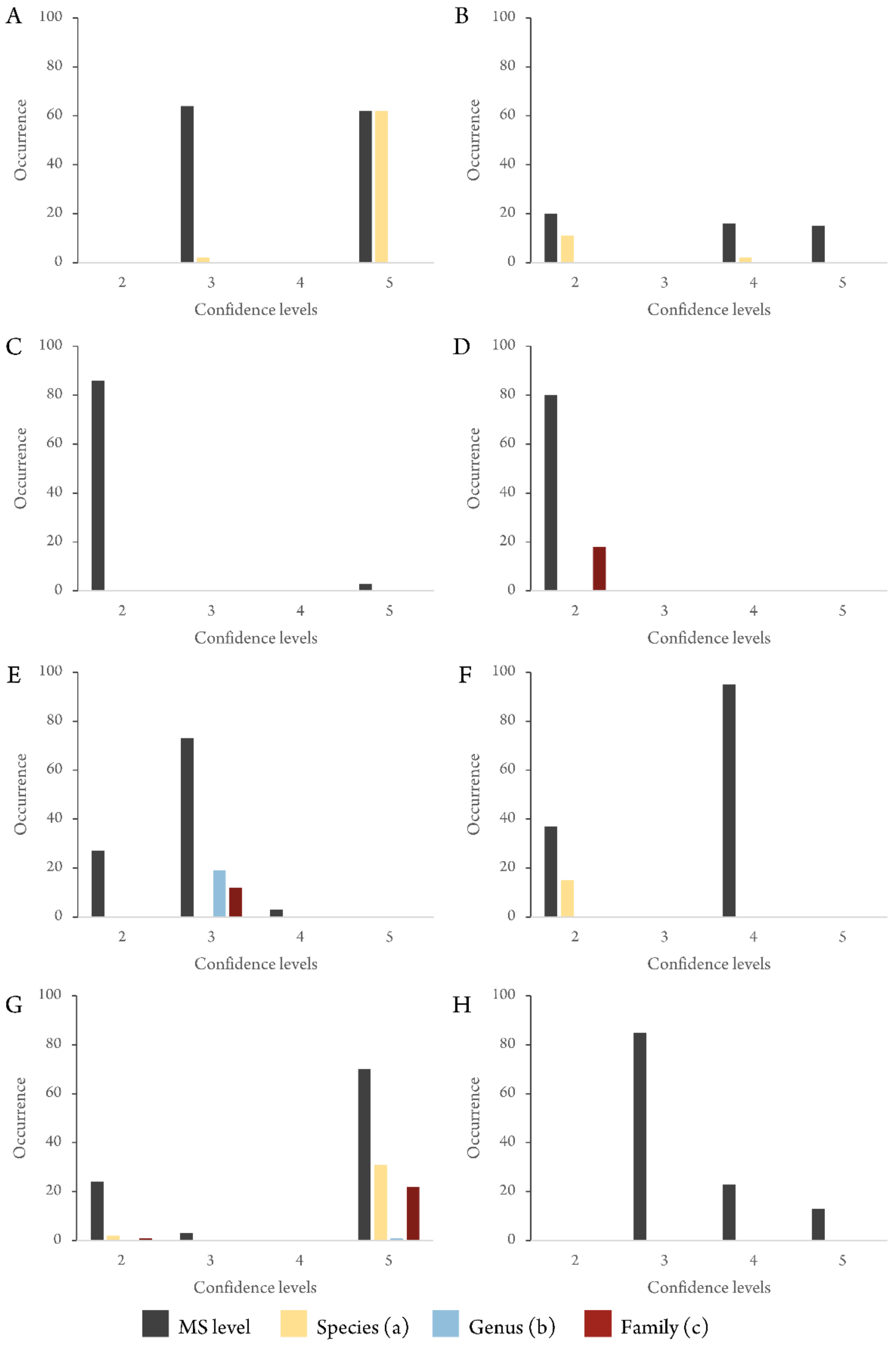

Figure S 15.
Reported confidence levels were disparate among participants. A) to H) Team 1 to 8, who used the datasets acquired on the Orbitrap-DDA in positive ionization. In black, the MS level (1 to 5), independently of the orthogonal levels. In yellow, blue, and dark red, the combination of MS levels with orthogonal taxonomic information.

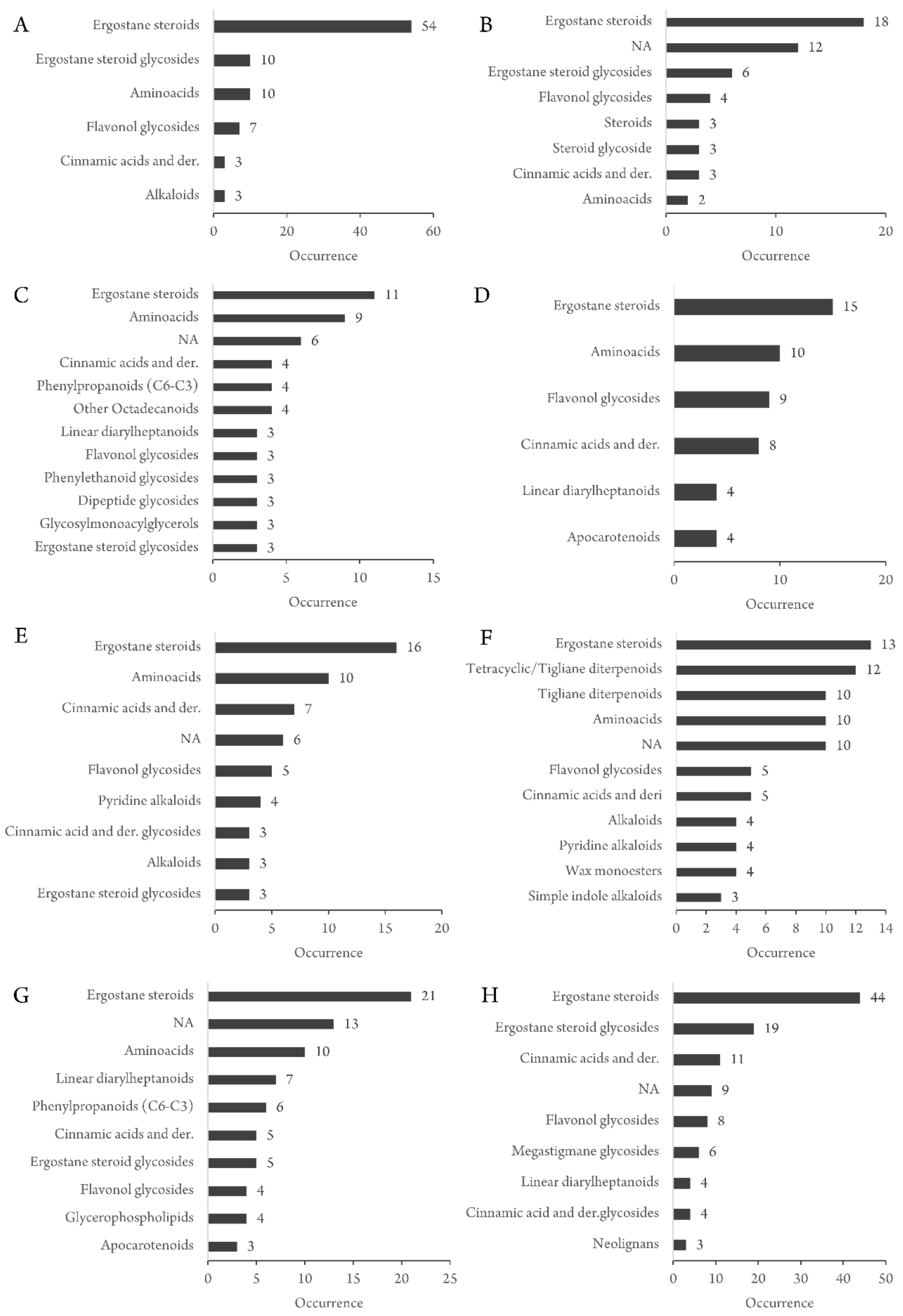

Figure S 16.

These histograms illustrate the chemical classes of the annotations that occurred at least three times among the participants who employed the datasets acquired on the Orbitrap-DDA in positive ionization. A) to H) Team 1 to 8. “Der.” stands for “derivatives”.

Section 8. From annotations of individual teams to consensus annotations

Preliminary analysis of the reported identities of analytes

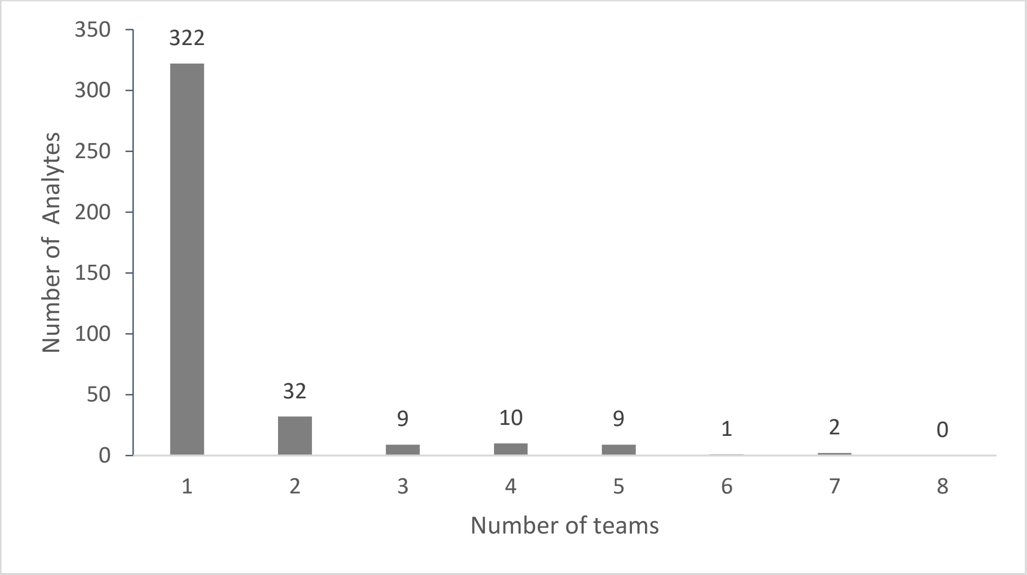

Figure S 17.
Number of analytes (defined as the same identity in a retention time window of ± 0.05 min) reported by 1 to 8 different teams in the Merged Annotation Table (SI-03, Table S1). To create this table, the identity elements (represented by the first moiety of the InchiKey) that were reported more than once in a retention time window of ± 0.05 min were highlighted and the number of different teams that reported the same identity was counted. Poor agreement was observed across the teams as to the identities assigned to the analytes.

Annotation Agreement Score

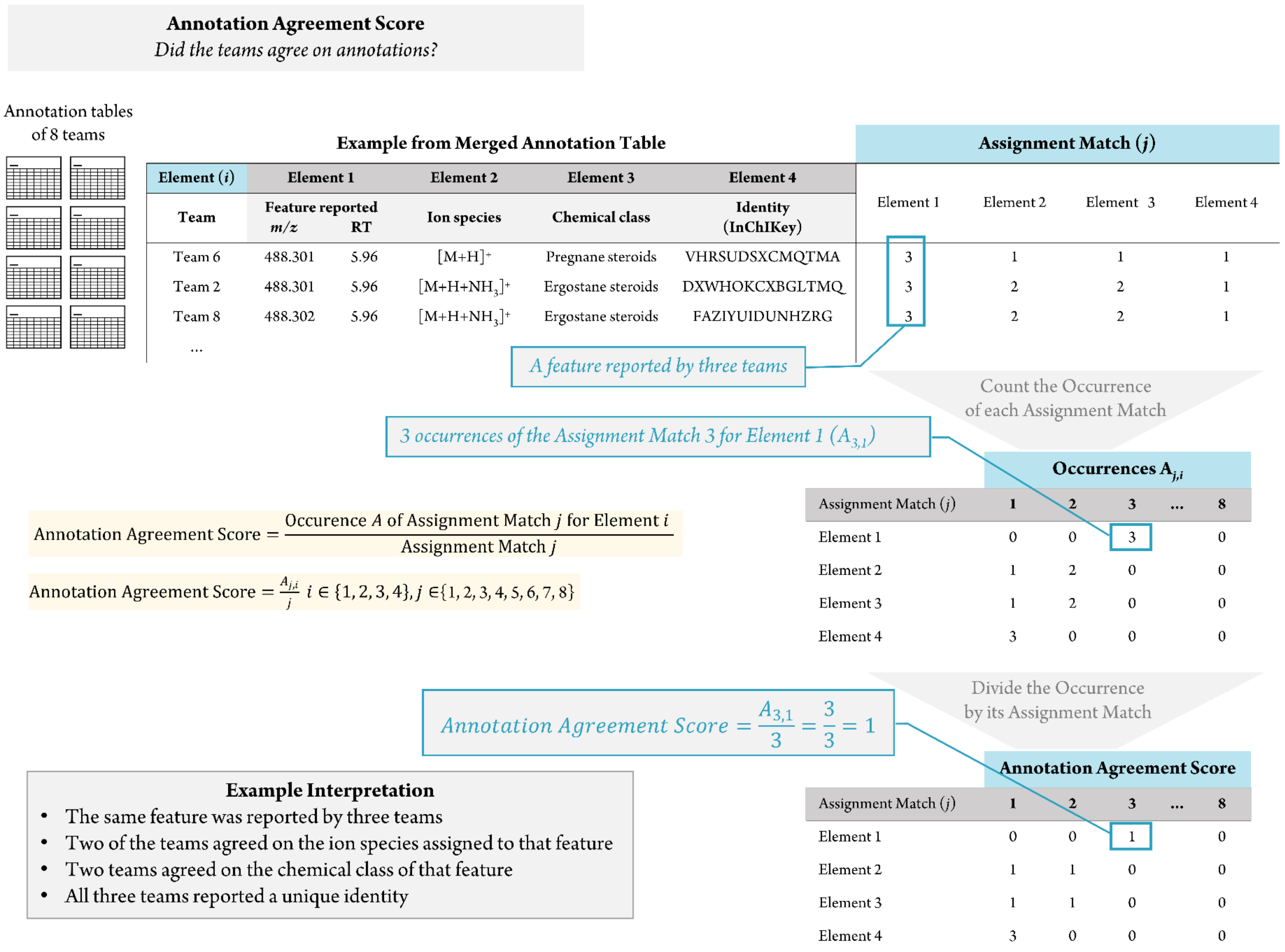

Figure S 18.
Description of the Annotation Agreement Score system that aimed at evaluating if the teams agreed with each other on annotations. It was applied on the Merged Annotation Table, which grouped the annotations of the 8 teams using the dataset acquired on the Orbitrap in positive ionization in the data dependent acquisition mode. See Fig.

1 and Fig. S19. In the example shown here, three teams reported the same feature and thus obtained an Assignment Match of 3 for Element 1. Two teams agreed on the ion species description (Element 2) and the chemical class (Element 3), so two teams obtained an Assignment Match of 2 for Element 2 and 3, while one team obtained an Assignment Match of 1. The teams did not agree on the identity, resulting in an Assignment Match of 1 for Element 4. Then we counted the Occurrence of each Assignment Match for each Element. Occurrence 3 was reached three times for Element 1 (reported feature), and Occurrence 2 was obtained twice for Elements 2 (ion species description) and Element 3 (chemical class). The Occurrences were next divided by the Assignment Match to adjust for features reported by more than one team and calculate the "Annotation Agreement Scores." This Annotation Agreement Score indicated that one feature was reported by three teams, that two teams agreed once on the ion species of one feature, that two teams agreed once on the chemical class of one feature, and that three teams reported a unique identity.

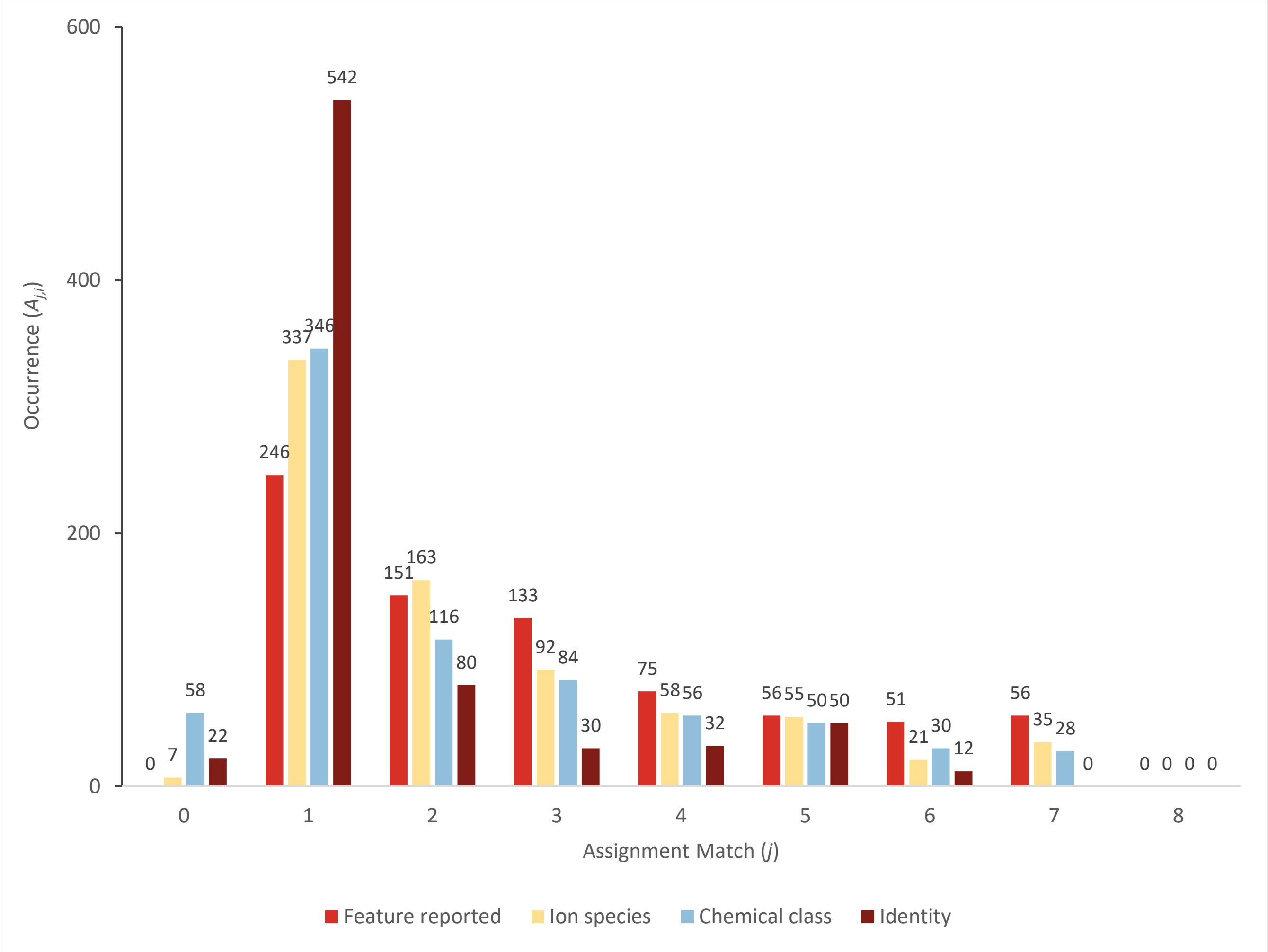

Figure S 19.

This histogram illustrates the Occurrence ($A_{j,i}$) for each Element in the Agreement Scores analysis, which assessed the agreement between the 8 teams who annotated the same dataset (Orbitrap data-dependent analysis (DDA) positive ionization) in the Merged Annotation Table. (see Fig. 1 and Fig. S18).

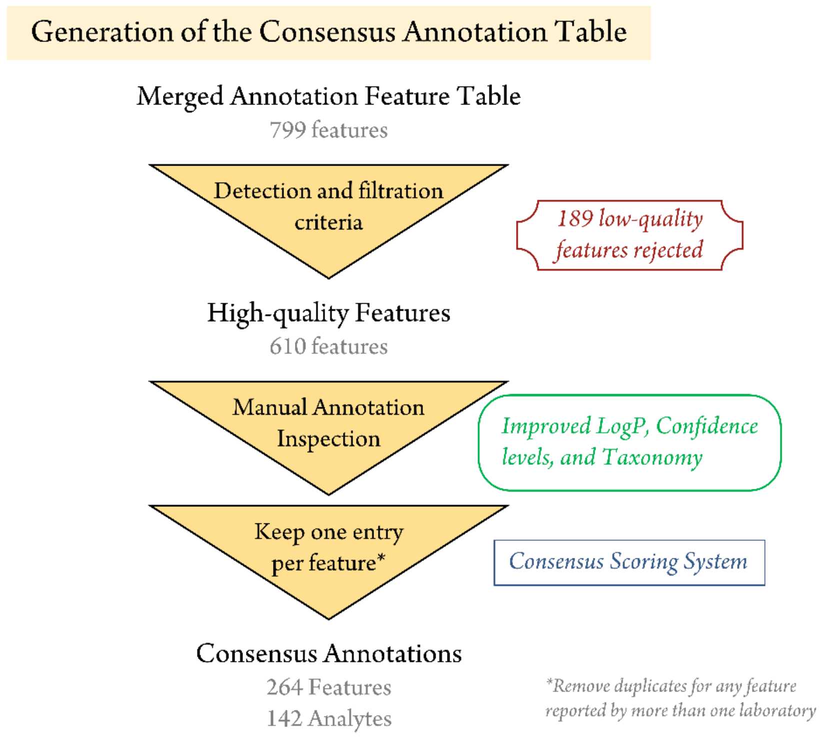

Figure S 20.
This figure presents the steps applied to generate the Consensus Annotation Table from the Merged Annotation Feature Table, which grouped the annotations of the 8 teams using the dataset acquired on the Orbitrap in positive ionization in the data dependent acquisition mode.

Consensus Annotation Scores

To what extent did the annotations reported by individual teams match the consensus annotations?

Example from Merged Annotation Table

	Element 1		Element 2	Element 3	Element 4
Lab	Feature reported *m/z*	RT	Ion species	Chemical class	Identity (InChIKey)
Lab 6	488.301	5.96	$[M+H]^+$	Pregnane steroids	VHRSUDSXCMQTMA
Lab 2	488.301	5.96	$[M+H+NH_3]^+$	Ergostane steroids	DXWHOKCXBGLTMQ
Lab 8	488.302	5.96	$[M+H+NH_3]^+$	Ergostane steroids	FAZIYUIDUNHZRG

A feature reported by three teams

Consensus Annotation Table

Element 1		Element 2	Element 3	Element 4
m/z	RT	Ion Species	Chemical class	Identity (InChIKey)
488.301	5.96	$[M+H+NH_3]^+$	Ergostane steroids	FAZIYUIDUNHZRG

Consensus Scores

		Lab 1	Lab 2	Lab 3	Lab 4	Lab 5	Lab 6	Lab 7	Lab 8
Element 1	Analyte reported	0	1	0	0	0	1	0	1
Element 2	Ion species	0	1	0	0	0	0	0	1
Element 3	Chemical class	0	1	0	0	0	0	0	1
Element 4	Identity	0	0	0	0	0	0	0	1

Calculating the sum of each score for each team

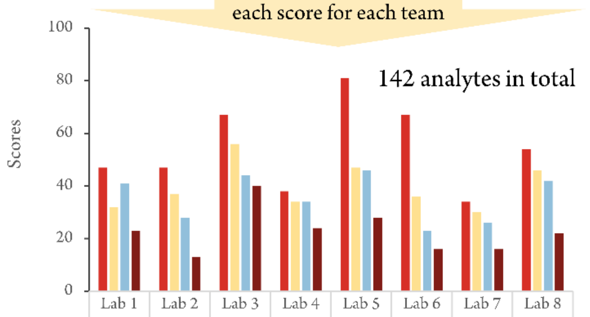

	Lab 1	Lab 2	Lab 3	Lab 4	Lab 5	Lab 6	Lab 7	Lab 8
Analytes	47	47	67	38	81	67	34	54
Ion species	32	37	56	34	47	36	30	46
Chemical class	41	28	44	34	46	23	26	42
Identity	23	13	40	24	28	16	16	22

Interpretation

- The teams reported between 34 (24%) and 81 (57%) analytes out of a a total of 142 analytes in the Consensus Analyte Table.
- Agreement by individual teams with the consensus annotation was reduced for annotation elements with greater complexity. For example, teams agreed more often with the ion species description than with the identity.

Figure S 21.
Description of the Consensus Annotation Score system, that aimed at evaluating to what extent the annotations reported by individual teams match the final consensus annotation. See Fig. 3 and S22.

A

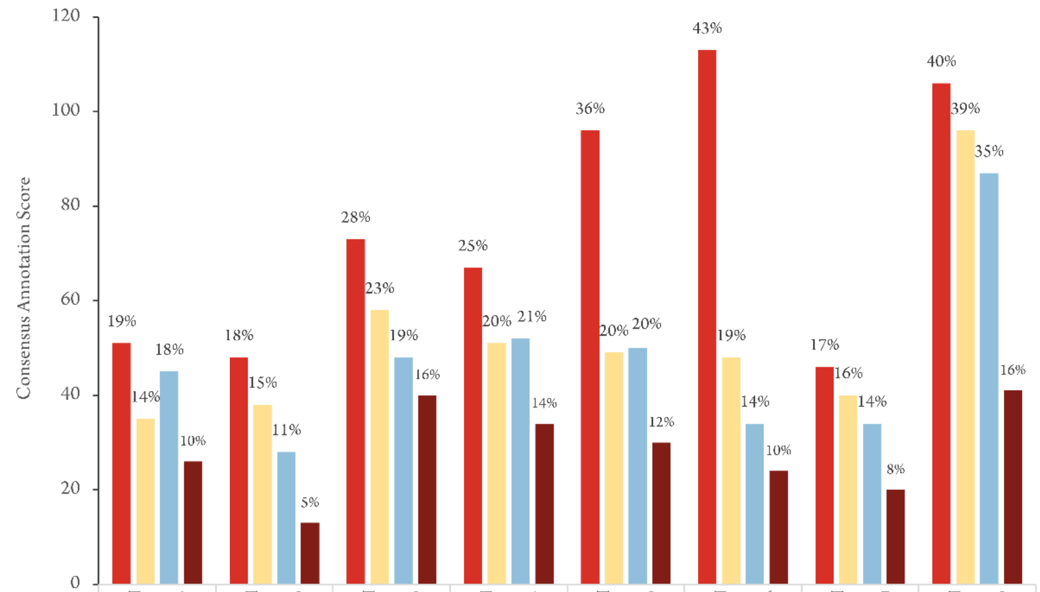

	Team 1	Team 2	Team 3	Team 4	Team 5	Team 6	Team 7	Team 8
Features	51	48	73	67	96	113	46	106
Ion species	35	38	58	51	49	48	40	96
Chemical class	45	28	48	52	50	34	34	87
Identity	26	13	40	34	30	24	20	41

B

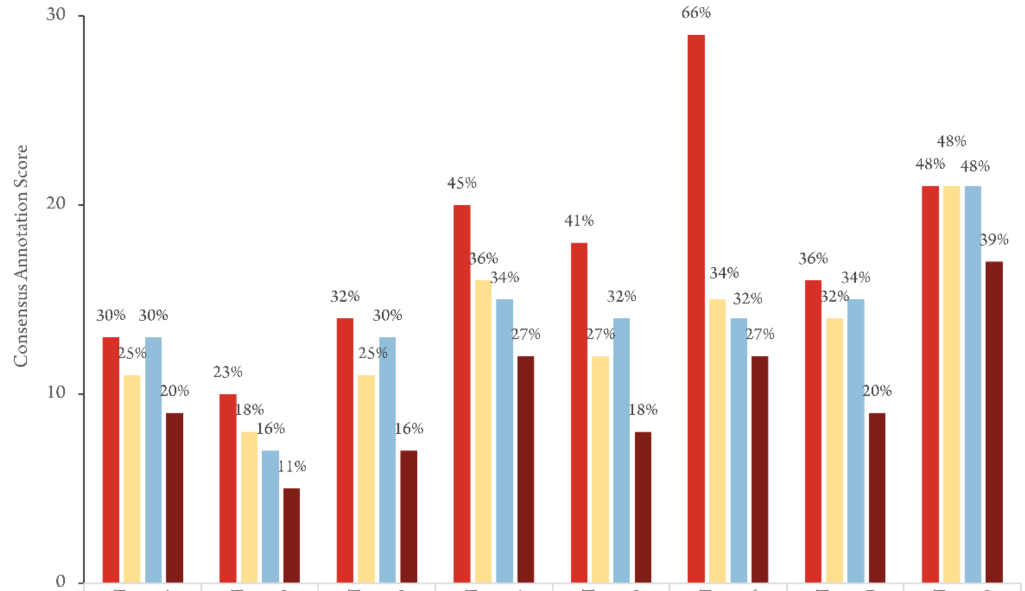

	Team 1	Team 2	Team 3	Team 4	Team 5	Team 6	Team 7	Team 8
Features	13	10	14	20	18	29	16	21
Ion species	11	8	11	16	12	15	14	21
Chemical class	13	7	13	15	14	14	15	21
Identity	9	5	7	12	8	12	9	17

Figure S 22.
These histograms illustrate the Consensus Annotation Score analysis (see experimental section and SI-01 Fig. 26), which aimed to assess the overlap between the individual lists provided by the 8 teams and the Consensus Annotation Table. A total of 264 features were annotated in the Consensus Annotation Table. The scores are provided in the table below the histograms, while the percentage of the total number of features s is shown on the histogram (*e.g.*, in panel A, Team 3 reported 73 features, which represents 28% of all reported features in the Consensus Annotation Table). Panel A shows the analyses for all features, and panel B) for the confidence level 1 annotations only, *i.e.*, the 13 confirmed by comparison with commercial standards.

Section 9. Description of annotations

This section grouped Figures obtained in the process of generating the Consensus Annotation Table. It also presents the differences between the annotation in the Merged Annotation Table and the Consensus Annotation Table in terms of ion species description, chemical classes and confidence level.

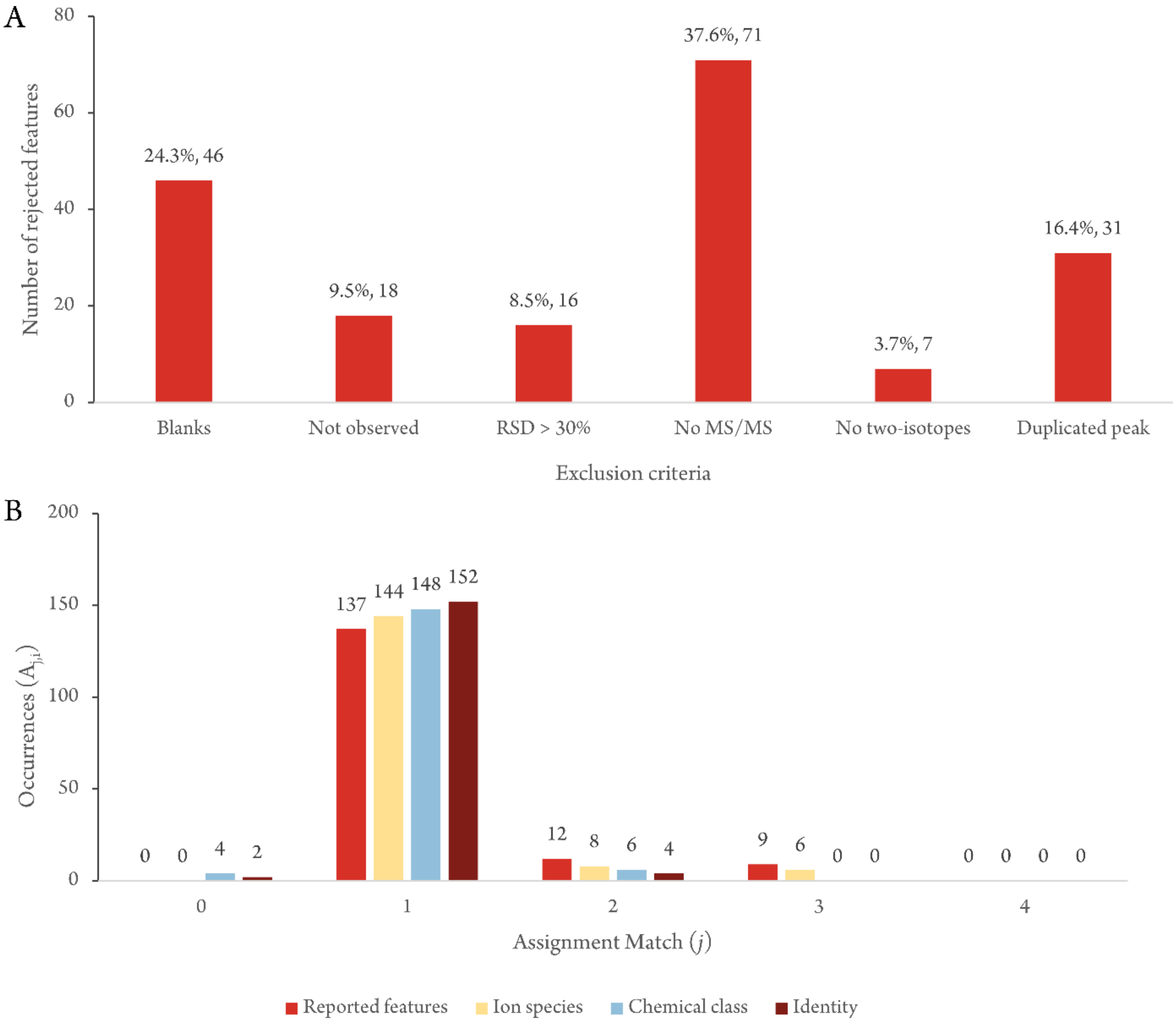

Figure S 23.
These histograms document the number of features rejected for the Consensus Annotation Table (189 features), and their Occurences in the Agreement score system (158 features, as the duplicate features were not considered for the Agreement Scores). A) Number of rejected features by type of exclusion criteria, and B) agreement scores for the 4 annotation elements for the 189 rejected features.

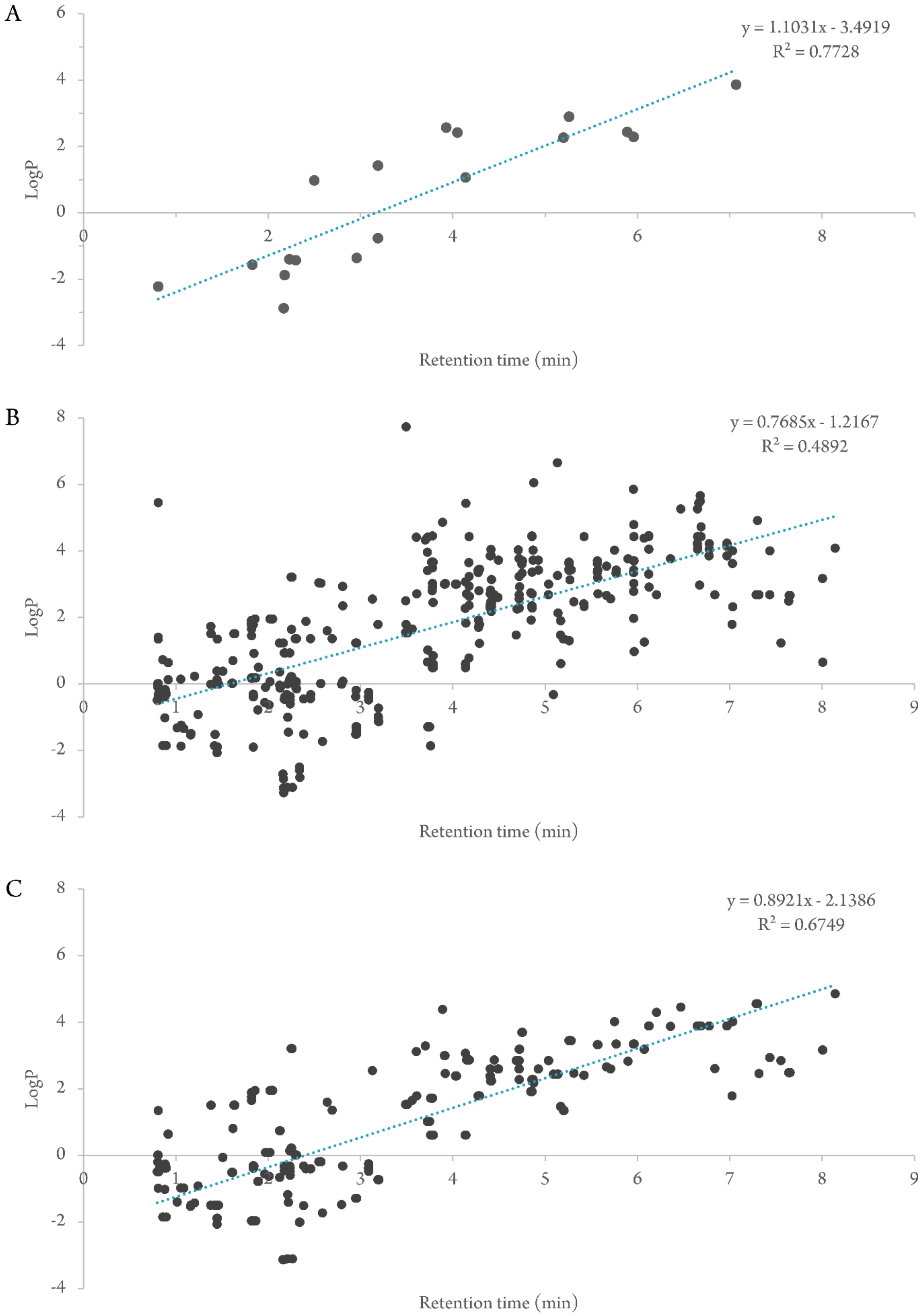

Figure S 24.
These scatter plots represent the LogPs of the identities of the annotation as a function of their retention time. A) for the pure commercial standards, for the features in B) the Merged Annotation Table, and C) the Consensus Annotation Table.

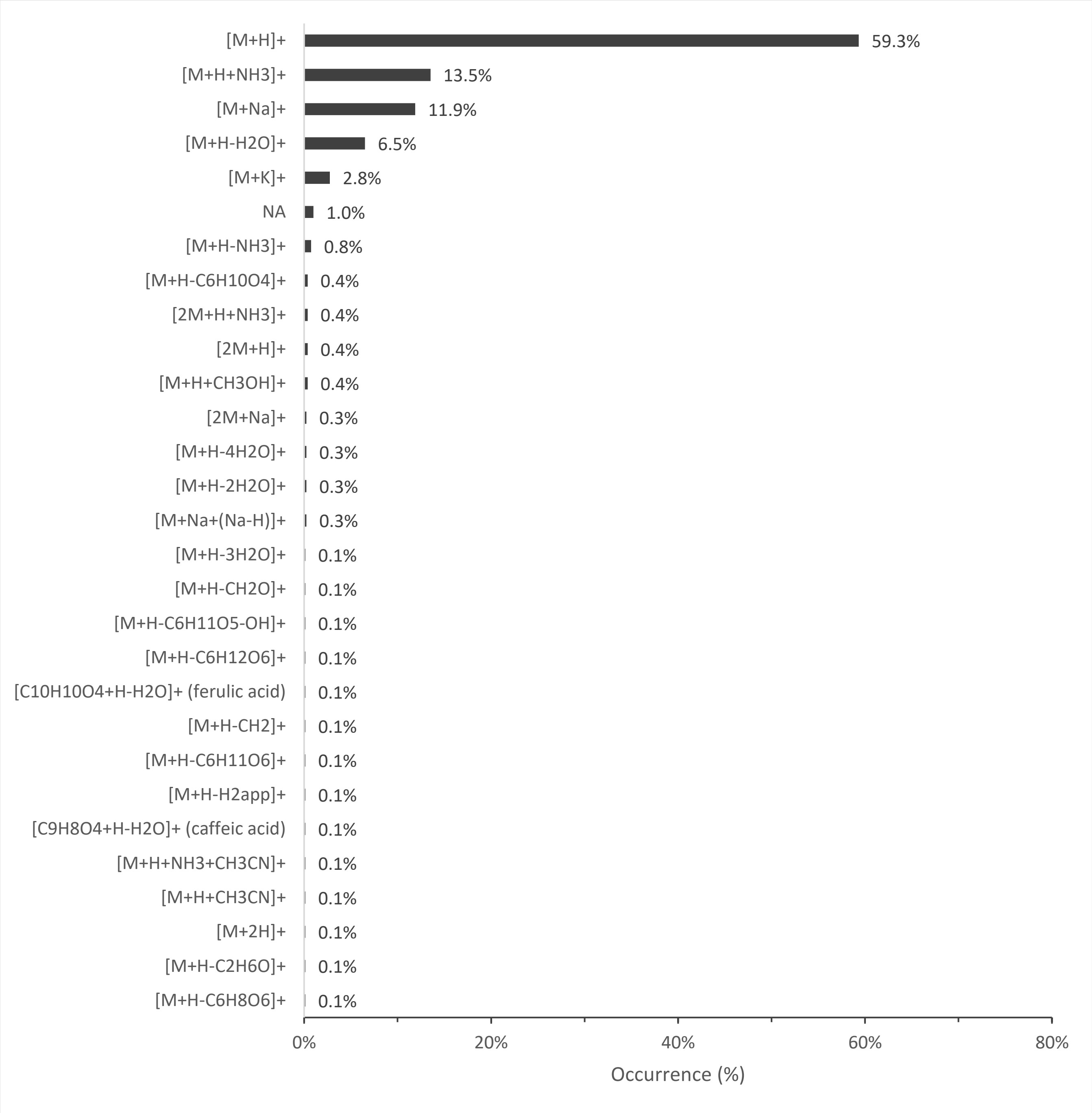

Figure S 25.
This histogram shows the type of ion species description proposed by the 8 teams for the features considered in the Merged Annotation Table (number of considered features = 799).

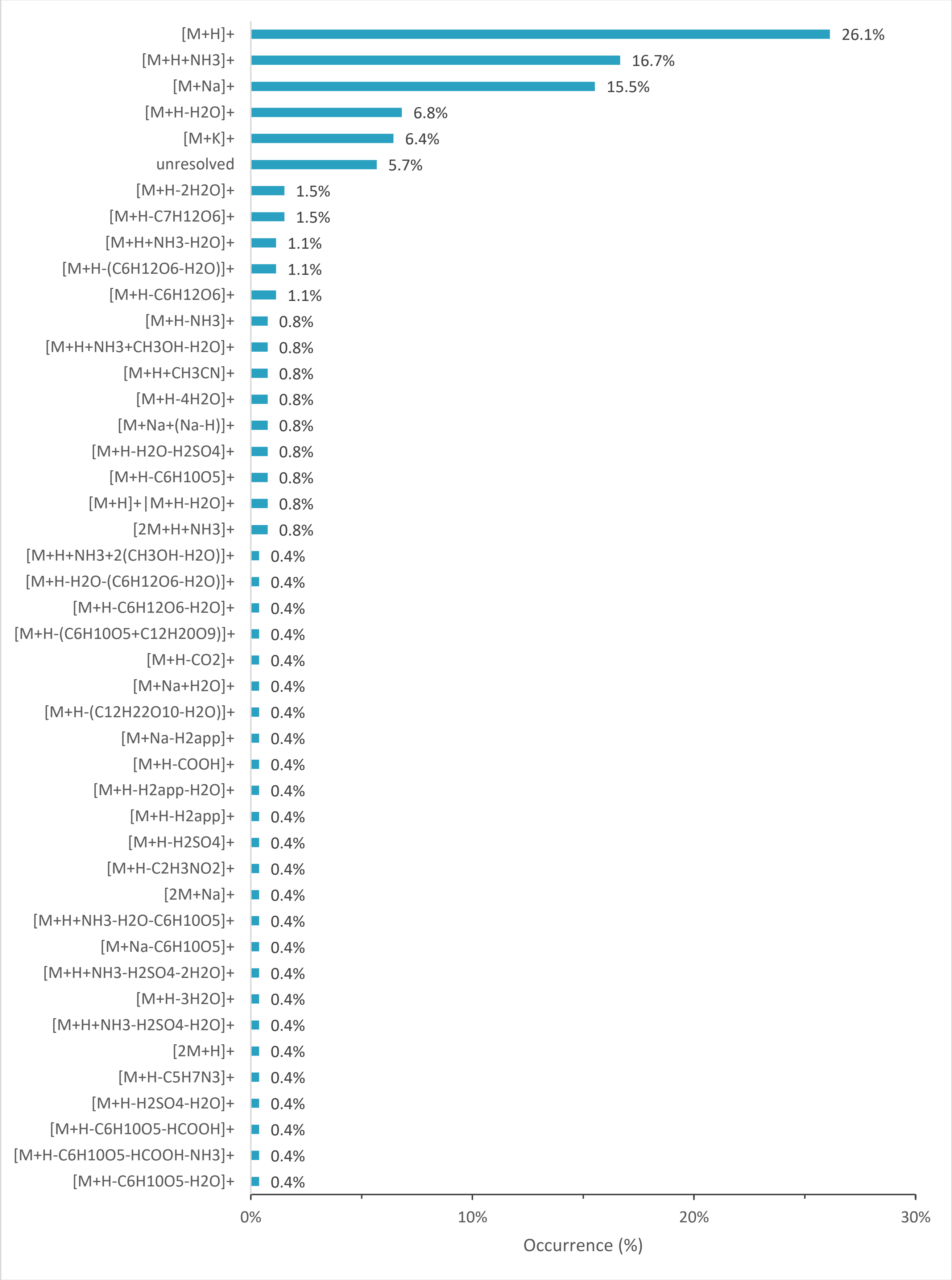

Figure S 26.
This histogram shows the type of ion species description proposed in the consensus annotation table (number of considered features = 264) after grouping the features reported by several teams.

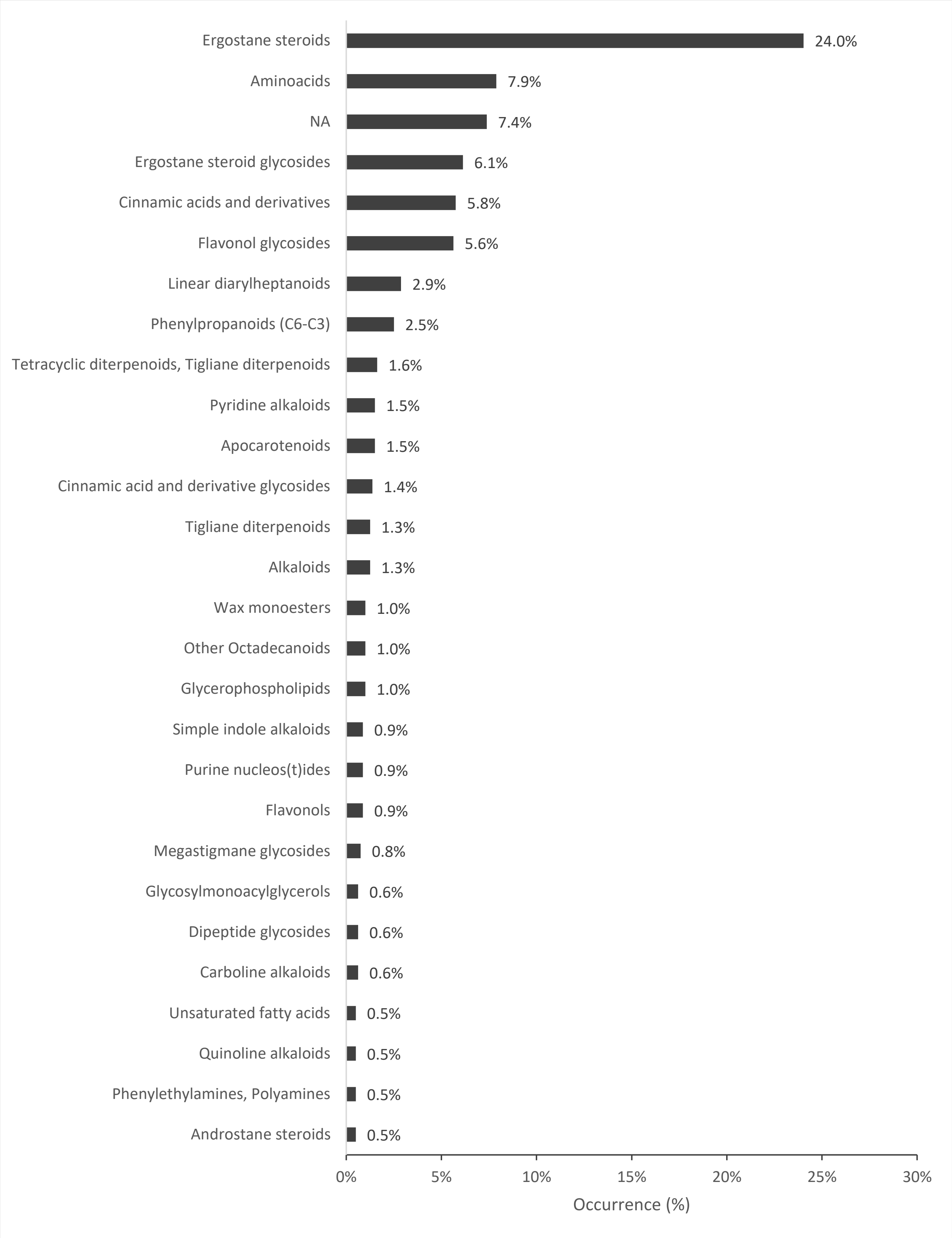

Figure S 27.
This histogram shows the type of NP classifier chemical classes proposed by the 8 teams for the features considered in the comparison analysis that occurred at least 4 times, which represents 81.0% of the 799 considered features.

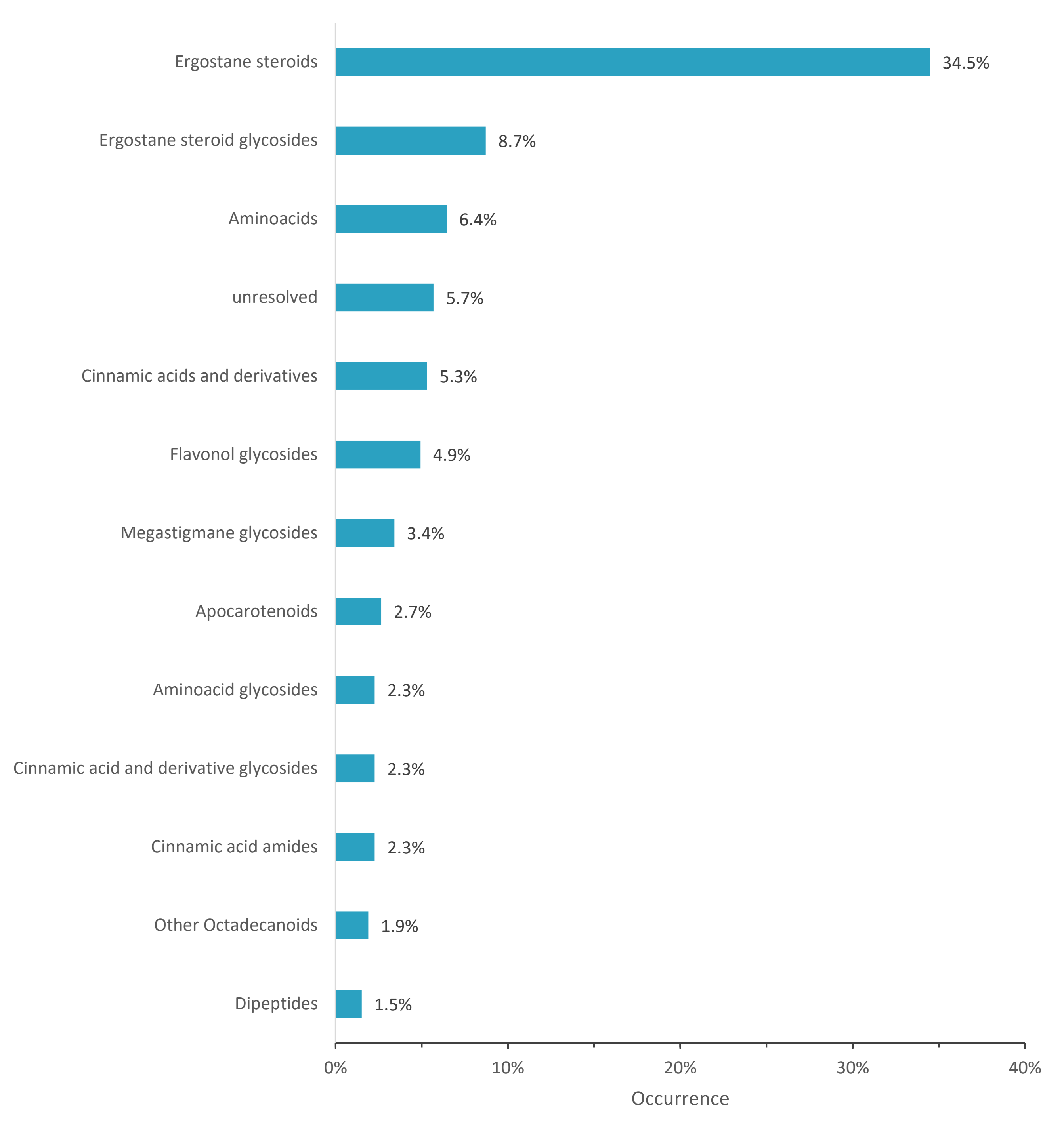

Figure S 28.
This histogram shows the type of NP classifier chemical classes proposed in the consensus annotation table that occurred at least 4 times, which represents 81.8% of the 264 considered features.

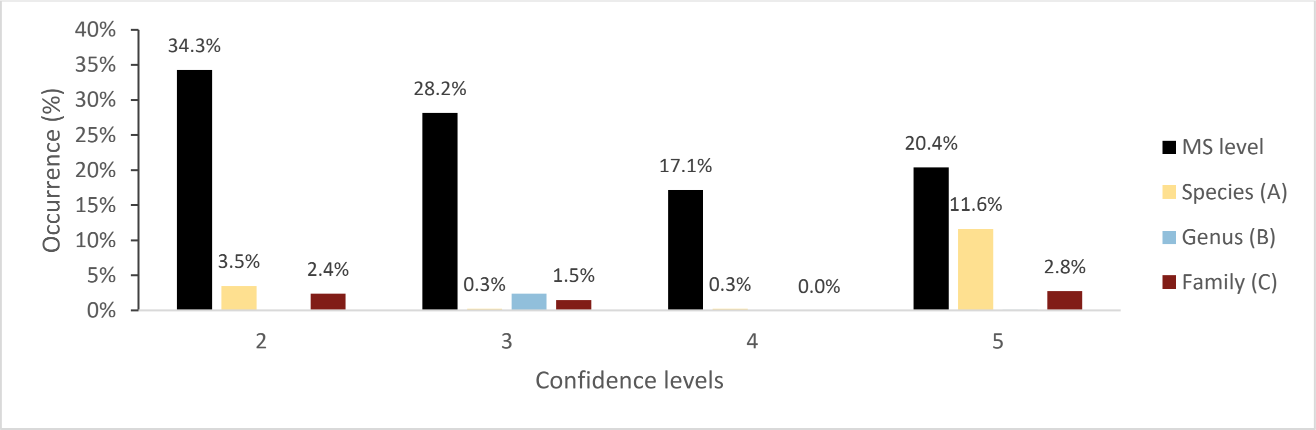

Figure S 29.
This histogram summarized the confidence levels proposed by the 8 teams for the features considered in the Merged Annotation Table (number of considered features = 799). In black, the MS level (2 to 5), independently of the orthogonal levels. In yellow, blue and dark red, the combination of MS levels with orthogonal taxonomic information.

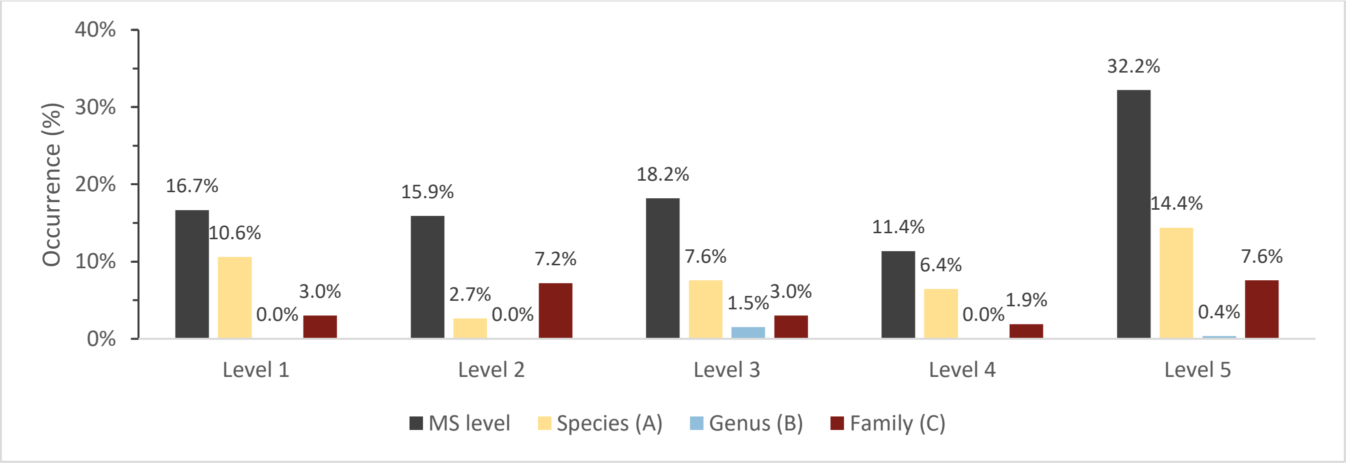

Figure S 30.
These histograms present the confidence levels obtained for the Consensus Annotation Table for the feature analysis (number of considered features = 264). In black, the MS level (2 to 5), independently of the orthogonal levels. In yellow, blue, and dark red, the combination of MS levels with orthogonal taxonomic information.

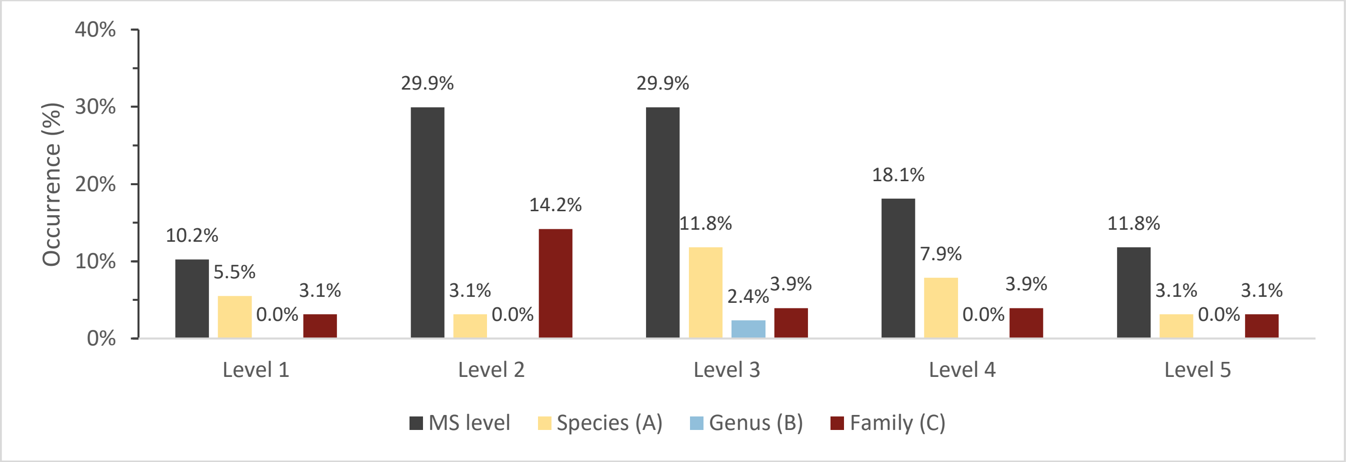

Figure S 31.
These histograms present the confidence levels obtained for the consensus annotation for the analyte analysis (number of considered analytes = 142). In black, the MS level (2 to 5), independently of the orthogonal levels. In yellow, blue, and dark red, the combination of MS levels with orthogonal taxonomic information.

Section 10. Standard annotations

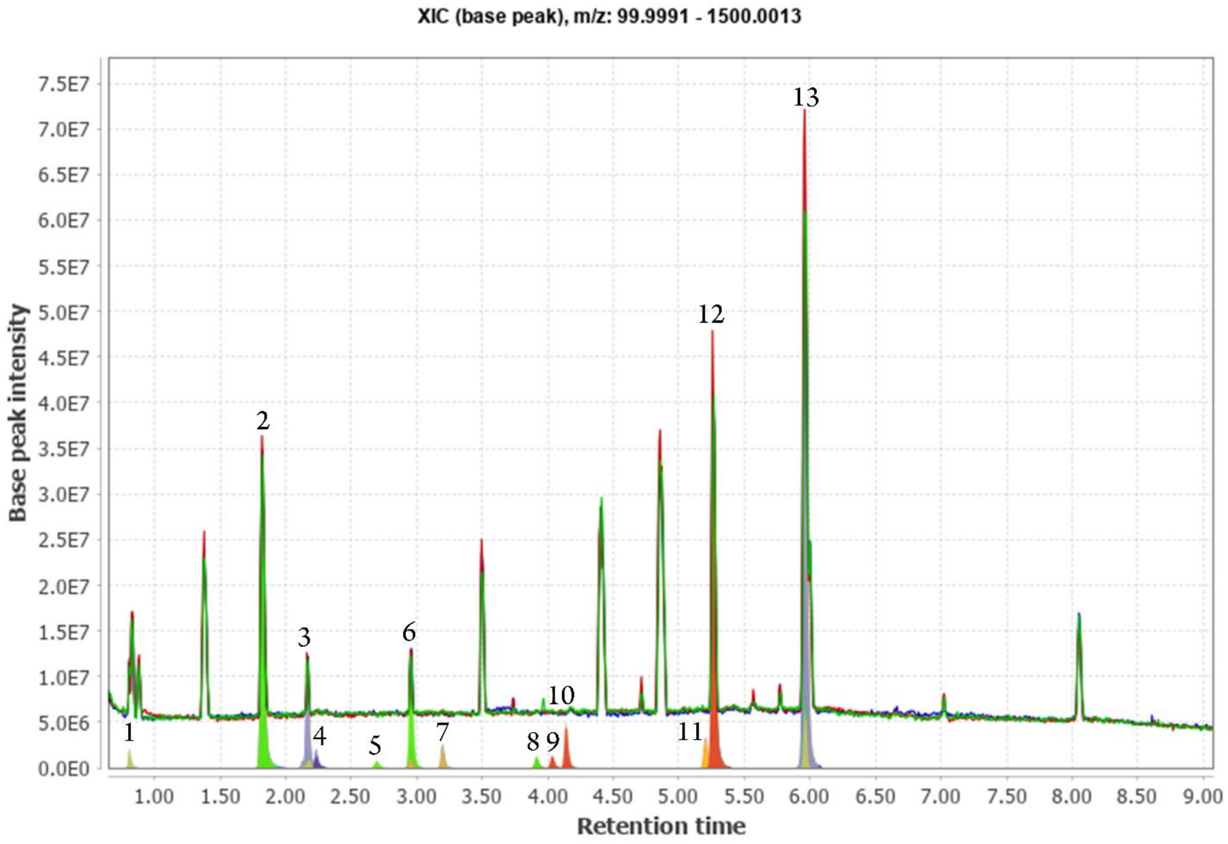

Figure S 32.
Analytes identified in *Withania somnifera* extract with a confidence level 1: 1) tyrosine, 2) *L*-tryptophan, 3) quercetin-3-*O*-rutinoside-7-*O*-glucoside, 4) cryptochlorogenic acid, 5) 3-feruloylquinic acid, 6) rutin, 7) kaempferol-3-*O*-rutinoside, 8) *N*-*p*-cumaroyltyramine, 9) *N*-feruloyltyramine, 10) withanoside IV, 11) withanoside V, 12) withaferin A, and 13) withanone.

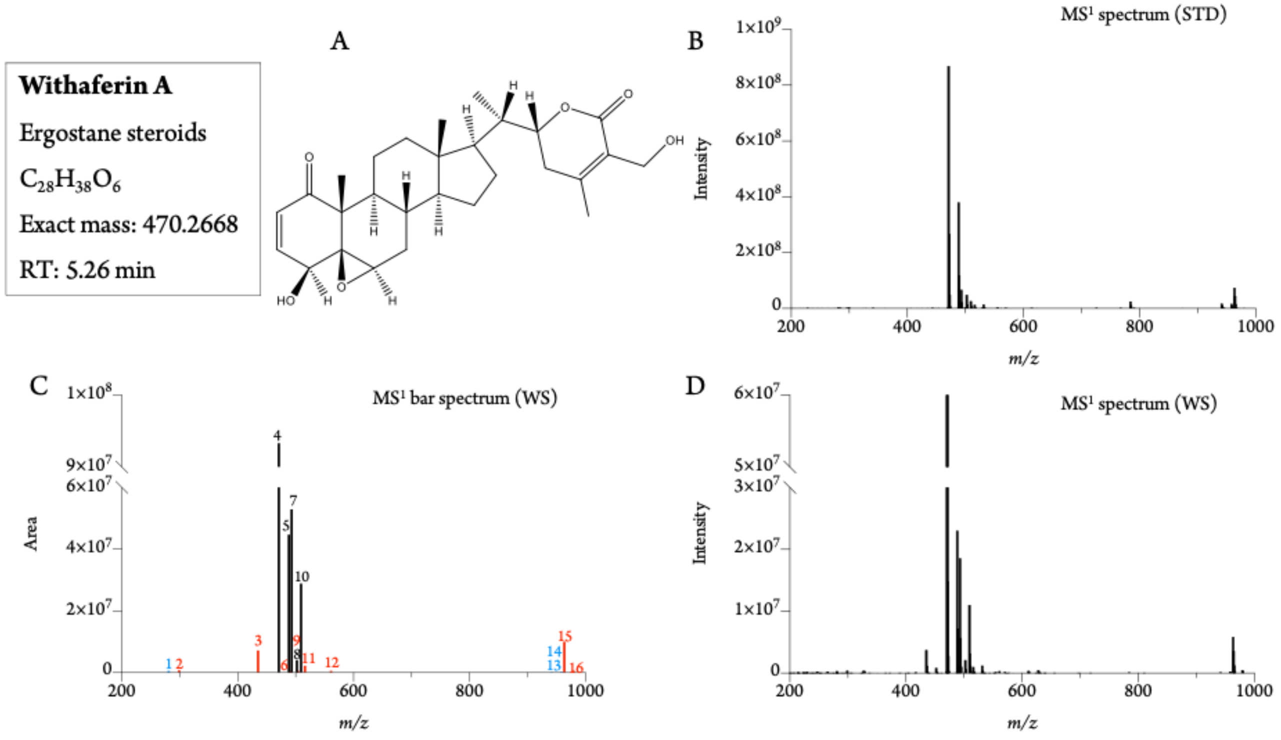

E

#	m/z	Annotation	Lab 1 [M+X]+	Lab 1 ID	Lab 2 [M+X]+	Lab 2 ID	Lab 3 [M+X]+	Lab 3 ID	Lab 4 [M+X]+	Lab 4 ID	Lab 5 [M+X]+	Lab 5 ID	Lab 6 [M+X]+	Lab 6 ID	Lab 7 [M+X]+	Lab 7 ID	Lab 8 [M+X]+	Lab 8 ID
1	281.1534	$[C_{19}H_{22}O_3\text{-}H_2O\text{+}H]^+$																
2	299.1638	$[C_{19}H_{22}O_3\text{+}H]^+$																
3	435.2534	$[M\text{+}H\text{-}2H_2O]^+$																
**4**	**471.2748**	$[M\text{+}H]^+$	$[M\text{+}H]^+$	c	$[M\text{+}H]^+$	✓	$[M\text{+}H]^+$	✓	$[M\text{+}H]^+$	✓	$[M\text{+}H]^+$	c	$[M\text{+}H]^+$	✓			$[M\text{+}H]^+$	✓
**5**	**488.3012**	$[M\text{+}H\text{+}NH_3]^+$											$[M\text{+}H\text{+}NH_3]^+$	✓			$[M\text{+}H\text{+}NH_3]^+$	✓
6	491.2409	$[M\text{+}Na\text{-}H_2{}^{app}]^+$																
**7**	**493.2566**	$[M\text{+}Na]^+$							$[M\text{+}Na]^+$	✓			$[M\text{+}Na]^+$	✓			$[M\text{+}Na]^+$	✓
**8**	**502.3168**	$[M\text{+}H\text{+}NH_3\text{+}CH_3OH\text{-}H_2O]^+$									$[M\text{+}H\text{-}(C_6H_{12}O_6\text{-}H_2O)]^+$	x	$[M\text{+}H]^+$	x				
9	504.2964	$[M\text{+}H\text{+}NH_3\text{+}H_2O\text{-}H_2{}^{app}]^+$																
**10**	**509.2301**	$[M\text{+}K]^+$											$[M\text{+}K]^+$	✓	$[M\text{+}K]^+$	c	$[M\text{+}K]^+$	✓
11	516.3326	$[M\text{+}H\text{+}2(CH_3OH\text{-}H_2O)]^+$																
12	561.2446	$[M\text{+}Na\text{+}NaCOOH]^+$																
13	941.5418	$[2M\text{+}H]^+$																
14	958.5680	$[2M\text{+}H\text{+}NH_3]^+$																
15	963.5238	$[2M\text{+}Na]^+$																
16	979.4975	$[2M\text{+}K]^+$																

Figure S 33.
LC-MS data and annotation of the analyte withaferin A (confidence level 1A). A) structure of withaferin A, B) MS^1 spectrum of the pure standard, C) MS^1 bar spectrum illustrating the ion species description, D) MS^1 spectrum of withaferin A in *Withania somnifera* extract, and F) annotation of its features and their detection by the 8 teams. $[M+X]^+$ means the ion species, ID means its identity ("x" false annotation, "✓" correct annotation, "c" correct chemical class but false annotation). The features in red were not reported by any teams, but were detected during our manual interpretation and by comparison with standards. The ones in blue did not have any MS/MS spectrum. Note that the features not reported by any participants (in red) are displayed here for documentation purposes but are not reported in the Consensus Table.

Section 11. Interpretation of the fragmentation spectra of the withanolides

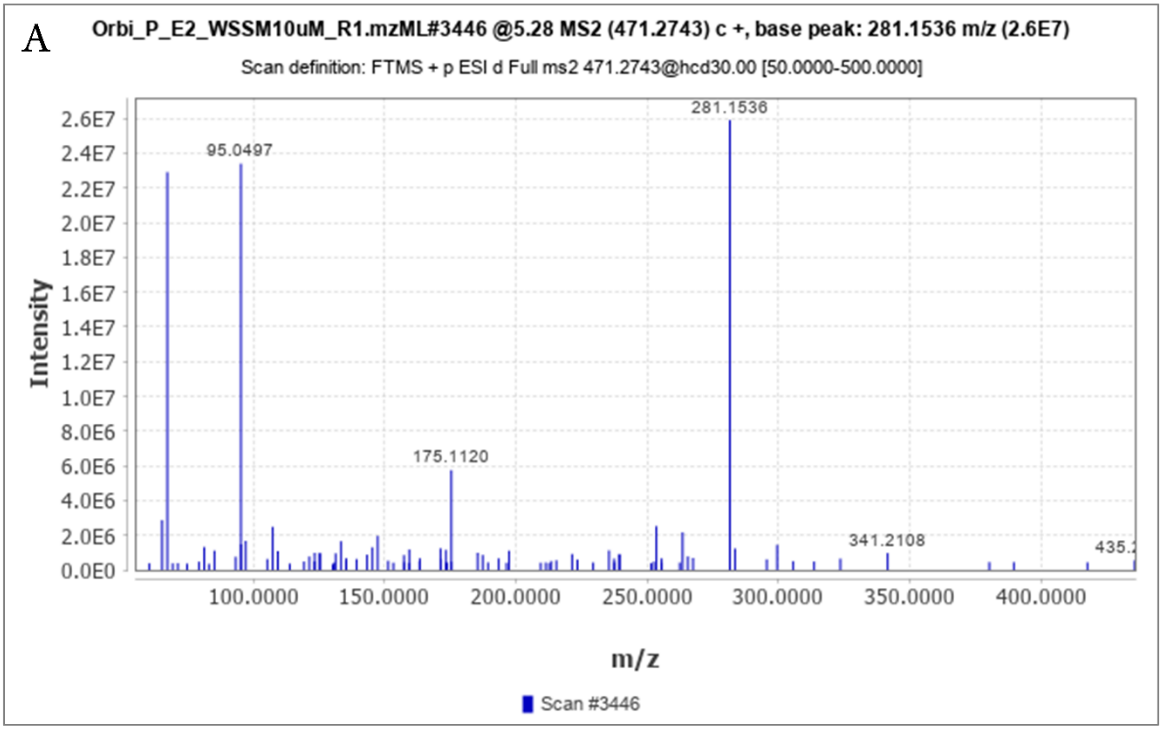

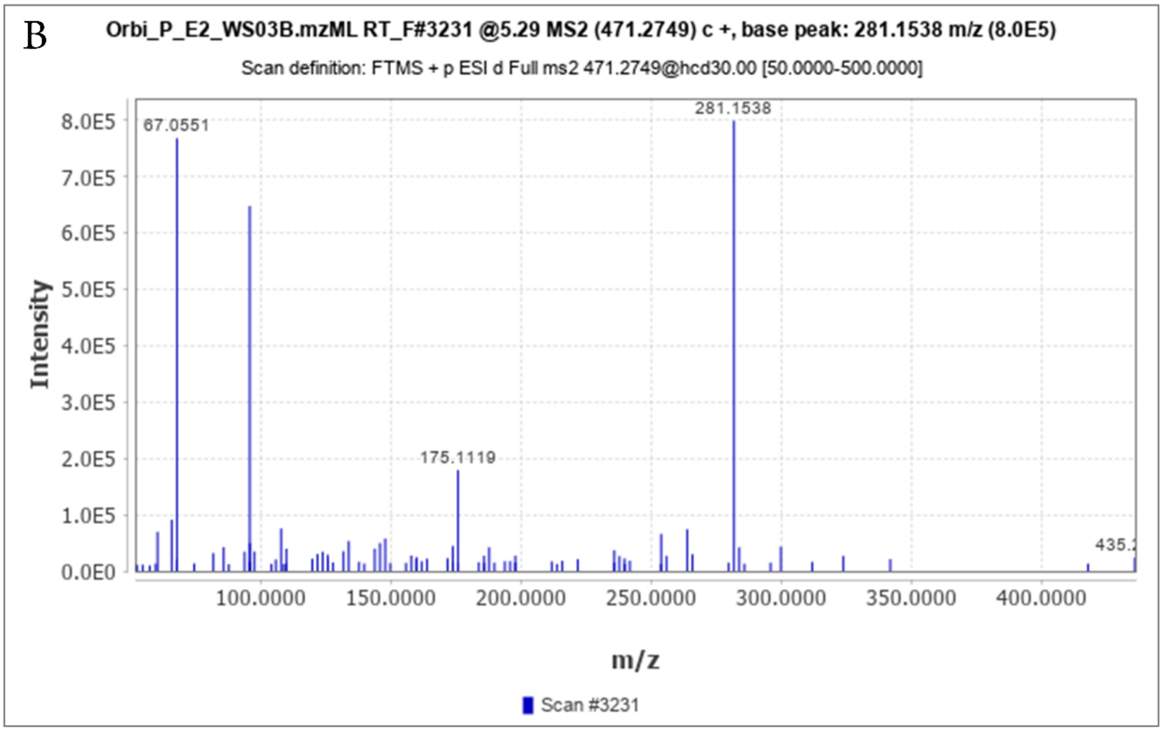

Figure S 34.

Fragmentation spectra from withaferin A ($[M+H]^+$): A) in the 10 uM standard mix (WSSM10uM) and B) in *Withania somnifera* methanolic extract (WS). The most intense fragment in the MS/MS spectrum of the protonated molecule of withaferin A was *m/z* 281, resulting from the losses of the lactone moiety and one water, which is a diagnostic ion for the 5,6-epoxywithanolides [23] (SI-01 Fig. S39). The following most intense fragments were *m/z* 95 and 67, which are the diagnostic ions for the 27-hydroxywithanolides [23].

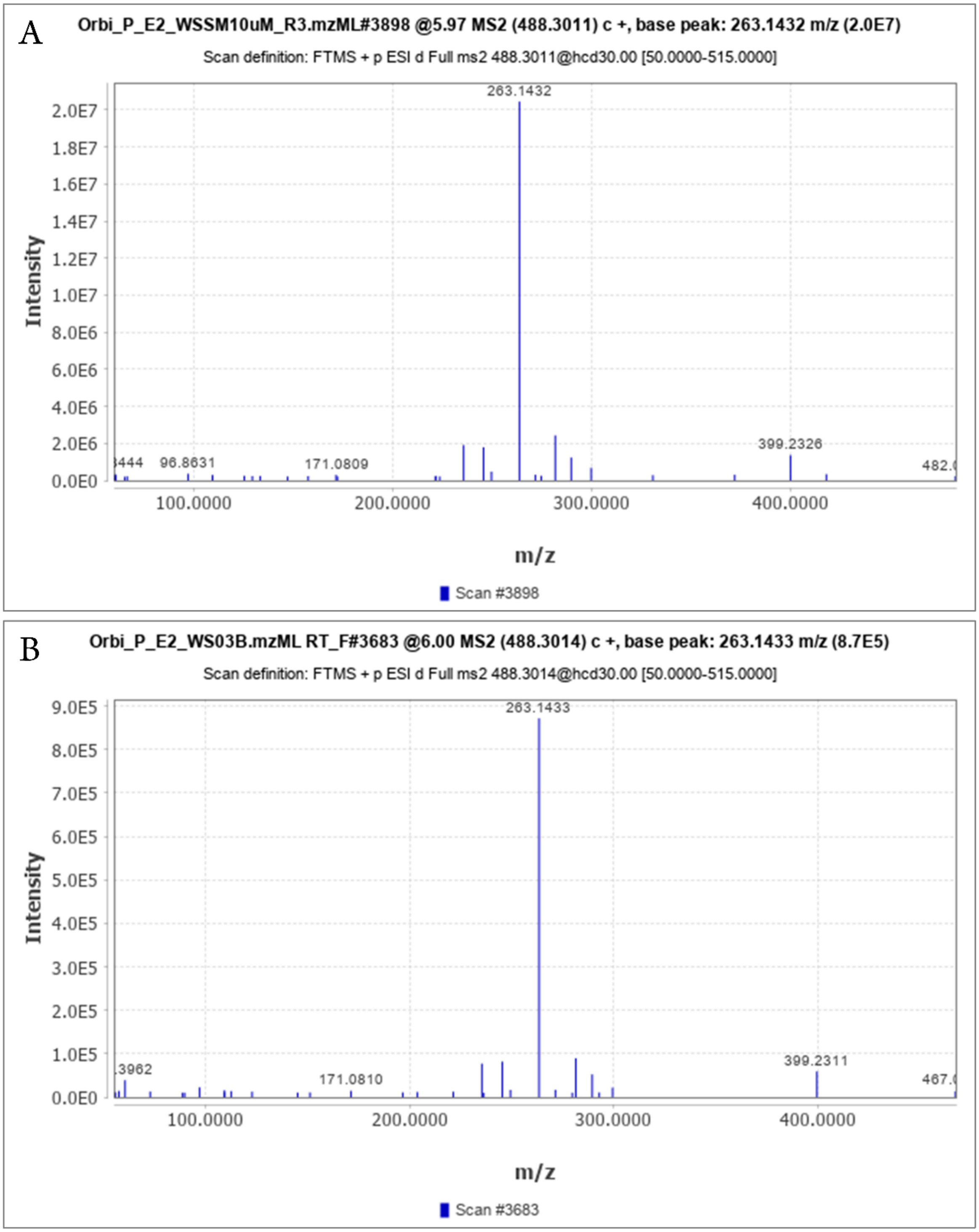

Figure S 35.

Fragmentation spectra from withanone ([M+H+NH3]+): A) in the 10 uM standard mix (WSSM10uM) and B) *Withania somnifera* methanolic extract (WS). The most intense feature for withanone was the ammonium adduct ($[M+H+NH_3]^+$ calc. *m/z* 488.3007), followed by the monodehydrated protonated molecule $[M+H-H_2O]^+$ calc. *m/z* 453.2636) The most intense fragment in both MS/MS spectra was *m/z* 263, resulting from the losses of the lactone moiety and two waters, which is a diagnostic ion for the 6, 7-epoxywithanolides [23] (SI-01 Fig. S39). The diagnostic ions for 5,6- and 6, 7-epoxywithanolides, and 27-hydroxywithanolides were employed to refine the annotation of other withanolides in *W. somnifera* extract as they were observed with these two standards in our instruments (SI-03 Table S2).

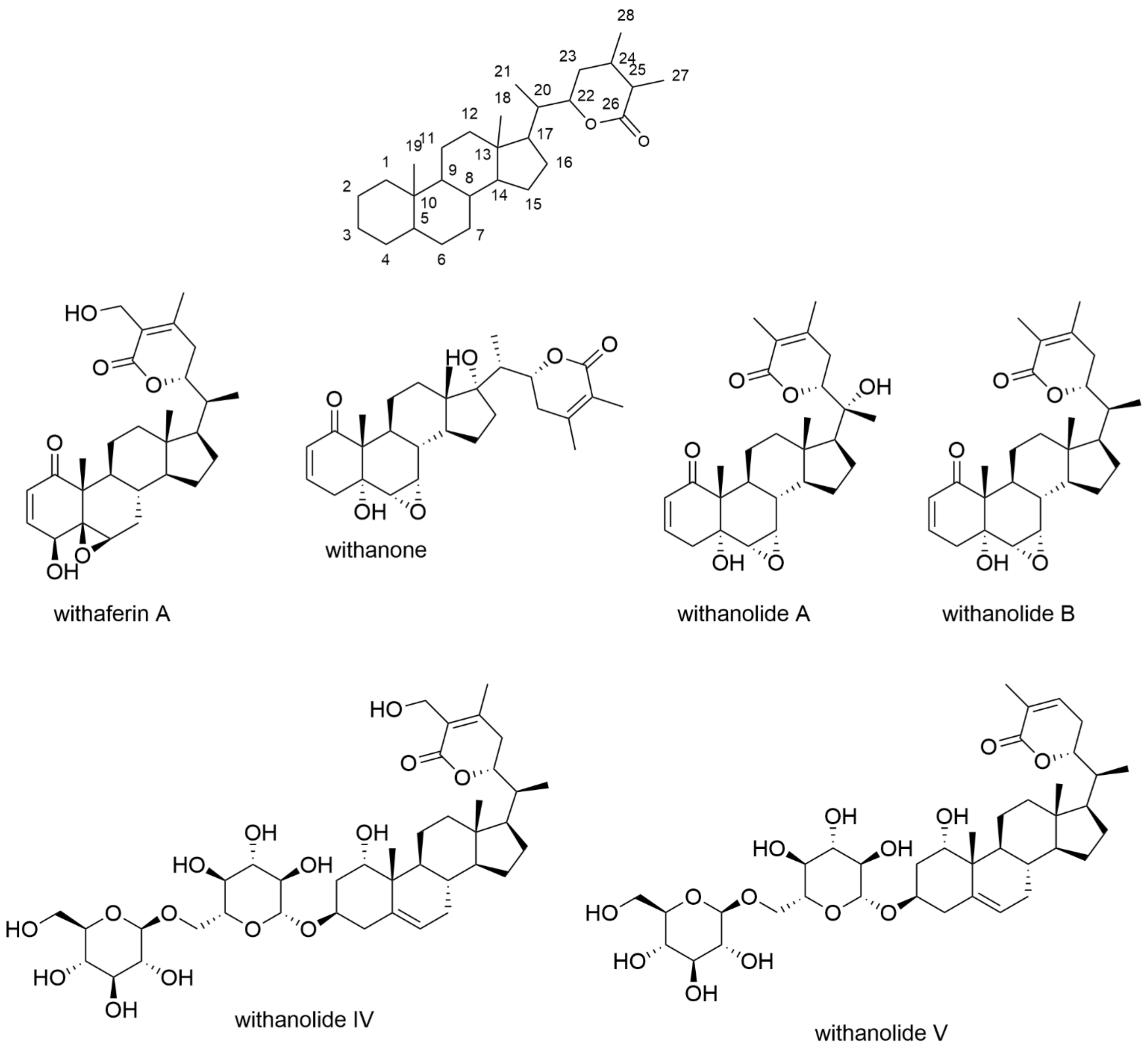

Figure S 36.
Structure of the withanolides analyzed as pure external standards.

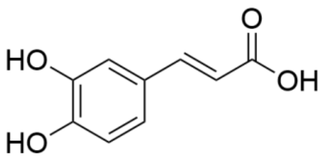

trans-caffeic acid

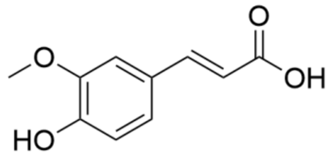

trans-ferulic acid

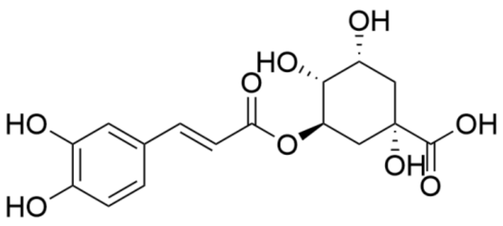

chlorogenic acid

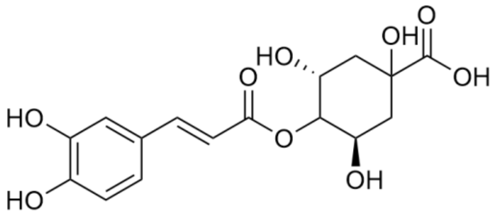

cryptochlorogenic acid

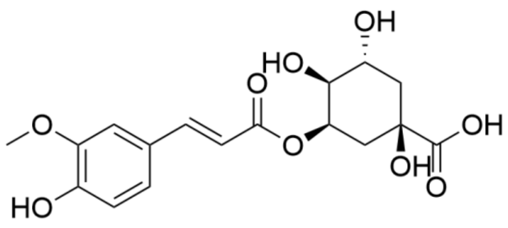

3-feruloylquinic acid

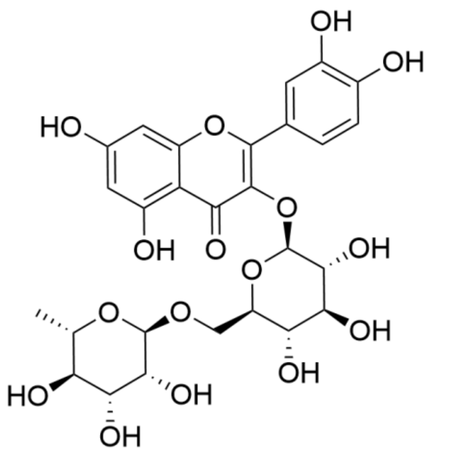

rutin

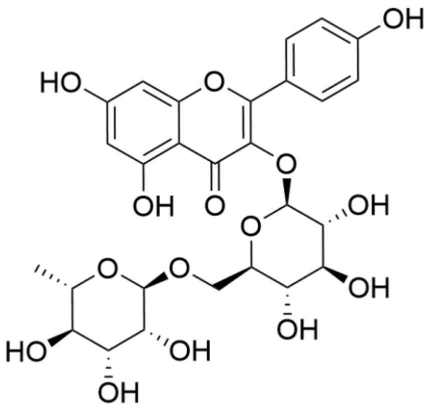

kaempferol-3-*O*-rutinoside

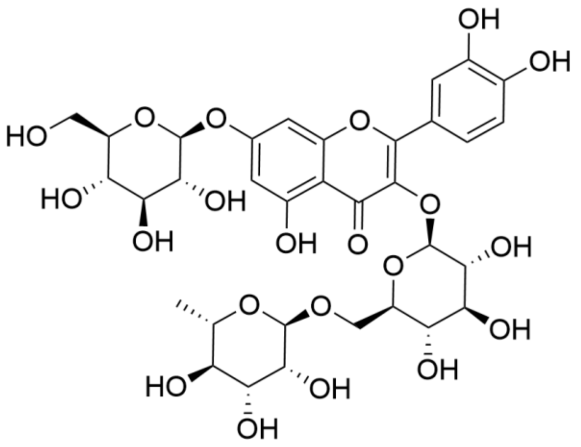

quercetin-3-*O*-rutinoside-7-*O*-glucoside

Figure S 37.
Structure of the flavonol glycosides and cinnamic acid derivatives analyzed as pure external standards.

Table S4: annotation of the features related to the analytes confirmed by external standards and their detection by the 8 teams.

**withanoside V**		**5.20 min**	**Team 1**		**Team 2**		**Team 3**		**Team 4**		**Team 5**		**Team 6**		**Team 7**		**Team 8**	
**#**	***m/z***	**Annotation**	**[M+X]+**	**ID**	**[M+X]+**	**ID**	**[M+X]+**	**ID**	**[M+X]+**	**ID**	**[M+X]+**	**ID**	**[M+X]+**	**ID**	**[M+X]+**	**ID**	**[M+X]+**	**ID**
1	605.3697	$[M+H-(C_6H_{12}O_6-H_2O)]^+$																
2	767.4234	$[M+H]^+$	[M+H]+	✓														
3	784.4493	$[M+H+NH_3]^+$	$[M+H+NH_3]^+$	✓	$[M+H+NH_3]^+$	x	$[M+H+NH_3]^+$	✓			$[M+H+NH_3]^+$	✓	[M+H]+	c			$[M+H+NH_3]^+$	✓
4	789.4042	$[M+Na]^+$																
5	805.3778	$[M+K]^+$																
**withanoside IV**		**4.14 min**	**Team 1**		**Team 2**		**Team 3**		**Team 4**		**Team 5**		**Team 6**		**Team 7**		**Team 8**	
**#**	***m/z***	**Annotation**	**[M+X]+**	**ID**	**[M+X]+**	**ID**	**[M+X]+**	**ID**	**[M+X]+**	**ID**	**[M+X]+**	**ID**	**[M+X]+**	**ID**	**[M+X]+**	**ID**	**[M+X]+**	**ID**
1	441.3004	$[M+H-2(C_6H_{12}O_6-H_2O)-H_2O]^+$																
2	459.3109	$[M+H-2(C_6H_{12}O_6-H_2O)]^+$																
3	621.3654	$[M+H-(C_6H_{12}O_6-H_2O)]^+$																
4	783.4179	[M+H]+																
5	800.4447	$[M+H+NH_3]^+$			$[M+H+NH_3]^+$	✓	$[M+H+NH_3]^+$	✓			$[M+H]^+$	x	$[M+H]^+$	x	$[M+H+NH_3]^+$	c	$[M+H+NH_3]^+$	✓
6	805.3996	$[M+Na]^+$											$[M+H]^+$	x	$[M+Na]^+$	c	$[M+Na]^+$	✓
7	821.3746	$[M+K]^+$															$[M+K]^+$	✓

$[M+X]^+$ means the ion species, ID means its identity ("x" false annotation, "✓" correct annotation, "c" correct chemical class but false annotation). The features in red were not reported by any teams, the ones in blue did not have any MS/MS spectrum.

cryptochlorogenic acid		2.23 min	Team 1		Team 2		Team 3		Team 4		Team 5		Team 6		Team 7		Team 8	
**#**	***m/z***	**Annotation**	**[M+X]+**	**ID**	**[M+X]+**	**ID**	**[M+X]+**	**ID**	**[M+X]+**	**ID**	**[M+X]+**	**ID**	**[M+X]+**	**ID**	**[M+X]+**	**ID**	**[M+X]+**	**ID**
1	163.0389	[M+H-C7H12O6]+	[M+H-H2O]+	c			[M+H-H2O]+	c			[M+H]+	c						
2	353.0864	[M+H-H2app]+																
3	355.1021	[M+H]+			[M+H]+	c	[M+H]+	c			[M+H]+	c	[M+H]+	✓	[M+H]+	c	[M+H]+	c
4	377.0841	[M+Na]+															[M+Na]+	c
**3-feruloylquinic acid**		**2.71 min**	**Team 1**		**Team 2**		**Team 3**		**Team 4**		**Team 5**		**Team 6**		**Team 7**		**Team 8**	
**#**	***m/z***	**Annotation**	**[M+X]+**	**ID**	**[M+X]+**	**ID**	**[M+X]+**	**ID**	**[M+X]+**	**ID**	**[M+X]+**	**ID**	**[M+X]+**	**ID**	**[M+X]+**	**ID**	**[M+X]+**	**ID**
1	177.0546	[M+H-C7H12O6]+									[M+H]+	c						
2	369.1177	[M+H]+																
**kaempferol-3-rutinoside**		**3.20 min**	**Team 1**		**Team 2**		**Team 3**		**Team 4**		**Team 5**		**Team 6**		**Team 7**		**Team 8**	
**#**	***m/z***	**Annotation**	**[M+X]+**	**ID**	**[M+X]+**	**ID**	**[M+X]+**	**ID**	**[M+X]+**	**ID**	**[M+X]+**	**ID**	**[M+X]+**	**ID**	**[M+X]+**	**ID**	**[M+X]+**	**ID**
1	287.0557	[M+H-(C12H22O10-H2O)]+																
2	595.1664	[M+H]+	[M+H]+	✓	[M+H]+	✓	[M+H]+	c	[M+H]+	x	[M+H]+	✓	[M+H]+	c	[M+H]+	✓		
3	617.1489	[M+Na]+							[M+Na]+	x								
4	633.1225	[M+K]+																

[M+X]$^+$ means the ion species, ID means its identity (“x” false annotation, “✓” correct annotation, “c” correct chemical class but false annotation). The features in red were not reported by any teams, the ones in blue did not have any MS/MS spectrum.

quercetin-3-*O*-rutinoside-7-*O*-glucoside		2.17 min	Team 1		Team 2		Team 3		Team 4		Team 5		Team 6		Team 7		Team 8	
**#**	***m/z***	**Annotation**	**[M+X]+**	**ID**	**[M+X]+**	**ID**	**[M+X]+**	**ID**	**[M+X]+**	**ID**	**[M+X]+**	**ID**	**[M+X]+**	**ID**	**[M+X]+**	**ID**	**[M+X]+**	**ID**
1	303.0497	[M+H-(C6H10O5+C12H20O9)]+											[M+H]+	x				
2	773.2140	[M+H]+	[M+H]+	c	[M+H]+	✓			[M+H]+	c	[M+H]+	✓	[M+H]+	✓	[M+H]+	✓	[M+H]+	c
3	788.2249	[M+H+NH3-H2app]+																
4	795.1957	[M+Na]+							[M+H]+	c							[M+H]+	c
**rutin**		**2.96 min**	**Team 1**		**Team 2**		**Team 3**		**Team 4**		**Team 5**		**Team 6**		**Team 7**		**Team 8**	
**#**	***m/z***	**Annotation**	**[M+X]+**	**ID**	**[M+X]+**	**ID**	**[M+X]+**	**ID**	**[M+X]+**	**ID**	**[M+X]+**	**ID**	**[M+X]+**	**ID**	**[M+X]+**	**ID**	**[M+X]+**	**ID**
1	301.0340	[M+H-(C12H22O10-H2O)-H2app]+																
2	303.0493	[M+H-(C12H22O10-H2O)]+					[M+H]+	x	[M+H]+	x			[M+H]+	x	[M+H]+	x		
3	465.1026	[M+H-(C6H12O5-H2O)]+					[M+H]+	c	[M+H]+	c								
4	611.1621	[M+H]+	[M+H]+	✓	[M+H]+	✓			[M+H]+	✓	[M+H]+	✓	[M+H]+	c	[M+H]+	✓	[M+H]+	✓
5	626.1729	[M+H+NH3-H2app]+																
6	633.1438	[M+Na]+	[M+Na]+	✓			[M+Na]+	✓	[M+Na]+	✓								

**feruloyl-tyramine**		**4.04 min**	**Team 1**		**Team 2**		**Team 3**		**Team 4**		**Team 5**		**Team 6**		**Team 7**		**Team 8**	
**#**	***m/z***	**Annotation**	**[M+X]+**	**ID**	**[M+X]+**	**ID**	**[M+X]+**	**ID**	**[M+X]+**	**ID**	**[M+X]+**	**ID**	**[M+X]+**	**ID**	**[M+X]+**	**ID**	**[M+X]+**	**ID**
1	314.1385	[M+H]+	[M+H]+	✓			[M+H]+	✓	[M+H]+	✓	[M+H]+	c			[M+H]+	✓	[M+H]+	✓
2	336.1206	[M+Na]+							[M+Na]+	✓			[M+Na]+	x			[M+Na]+	✓
3	352.0943	[M+K]+															[M+K]+	✓

***N-p*-cumaroyl-tyramin**		**3.92 min**	**Team 1**		**Team 2**		**Team 3**		**Team 4**		**Team 5**		**Team 6**		**Team 7**		**Team 8**	
**#**	***m/z***	**Annotation**	**[M+X]+**	**ID**	**[M+X]+**	**ID**	**[M+X]+**	**ID**	**[M+X]+**	**ID**	**[M+X]+**	**ID**	**[M+X]+**	**ID**	**[M+X]+**	**ID**	**[M+X]+**	**ID**
1	284.1281	[M+H]+			[M+X]+	x	[M+H]+	✓	[M+H]+	✓	[M+H]+	✓	[M+H]+	✓	[M+H-CH2O]+	c	[M+H]+	✓
2	306.1099	[M+Na]+							[M+Na]+	✓			[M+Na]+	✓				
3	322.0838	[M+K]+																

***L*-tryptophan**		**1.83 min**	**Team 1**		**Team 2**		**Team 3**		**Team 4**		**Team 5**		**Team 6**		**Team 7**		**Team 8**	
**#**	***m/z***	**Annotation**	**[M+X]+**	**ID**	**[M+X]+**	**ID**	**[M+X]+**	**ID**	**[M+X]+**	**ID**	**[M+X]+**	**ID**	**[M+X]+**	**ID**	**[M+X]+**	**ID**	**[M+X]+**	**ID**
	159.0916	[M+H-HCOOH]+									[M+H]+	x						
	188.0705	[M+H-NH3]+	[M+H-NH3]+	✓	[M+H]+	x	[M+H-NH3]+	✓	[M+H]+	x			[M+H]+	x	[M+H-NH3]+	✓		
	205.0971	[M+H]+	[M+H]+	✓					[M+H]+	✓	[M+H]+	✓	[M+H+NH3]+	x	[M+H]+	✓	[M+H]+	✓
	227.0789	[M+Na]+							[M+Na]+	✓			[M+Na]+	✓	[M+Na]+	✓		
	243.0529	[M+K]+									[M+K]+	✓						
	409.1867	[2M+H]+											[2M+H]+	✓	[2M+H]+	✓		

***DL*-tyrosine**		**0.81 min**	**Team 1**		**Team 2**		**Team 3**		**Team 4**		**Team 5**		**Team 6**		**Team 7**		**Team 8**	
**#**	***m/z***	**Annotation**	**[M+X]+**	**ID**	**[M+X]+**	**ID**	**[M+X]+**	**ID**	**[M+X]+**	**ID**	**[M+X]+**	**ID**	**[M+X]+**	**ID**	**[M+X]+**	**ID**	**[M+X]+**	**ID**
1	165.0547	[M+H-NH3]+	[M+H-NH3]+	✓					[M+H-NH3]+	✓								
2	182.0813	[M+H]+	[M+H]+	✓					[M+H]+	✓	[M+H]+	✓	[M+H]+	✓	[M+H]+	✓		

Section 12. Viewing features in the metabolite profiling

A

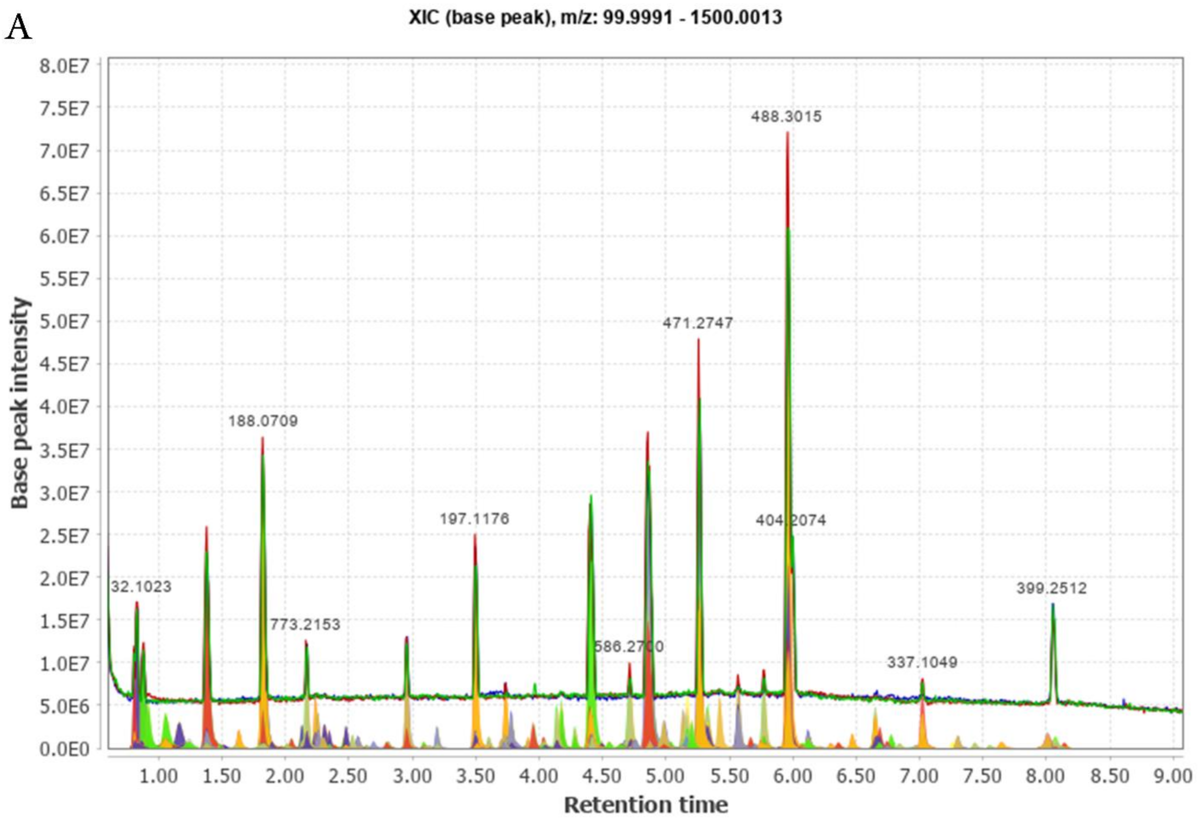

B

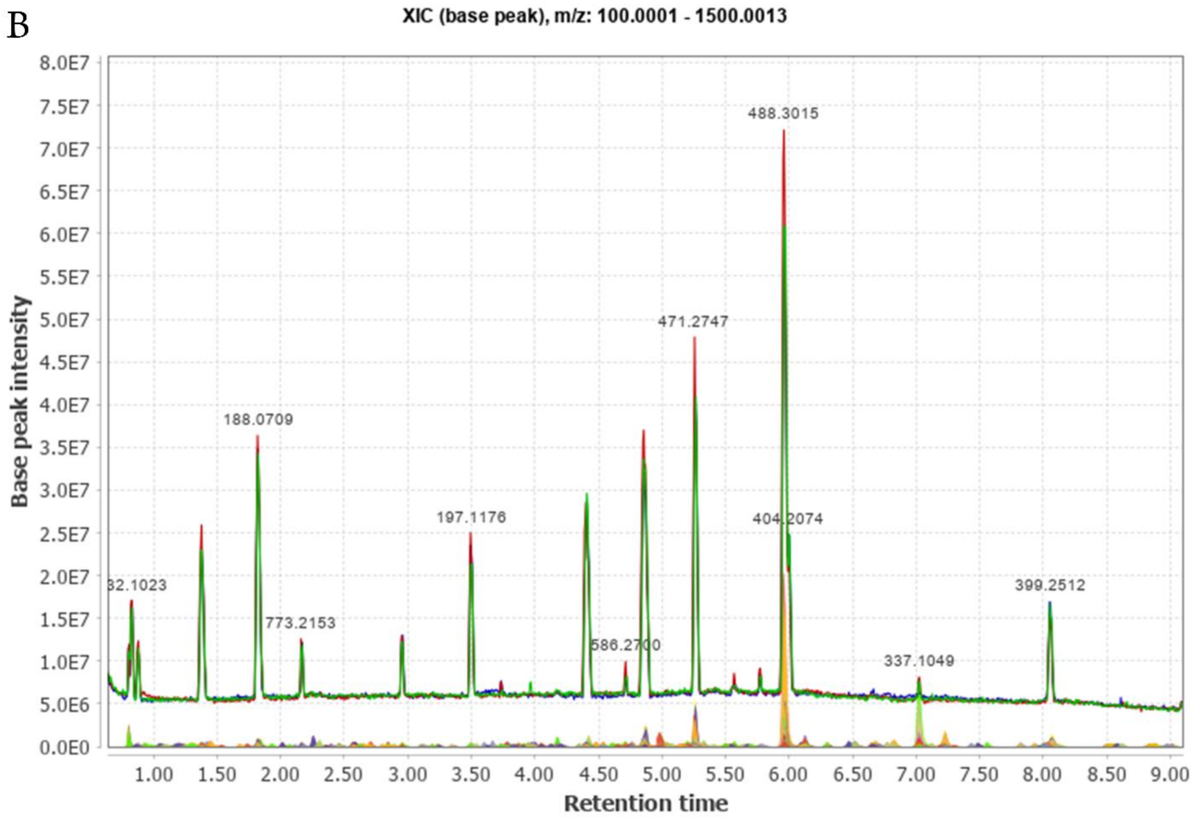

Figure S 38.
A) viewing the high-quality features considered in the Consensus Annotation Table in the process LC-MS data of the team which initiated the project. B) Viewing the remaining high-quality features not included in the Consensus Annotation Table, because they were not reported by any participating team.

Section 13. References

(1) Müller, E.; Huber, C. E.; Brack, W.; Krauss, M.; Schulze, T. Symbolic Aggregate Approximation Improves Gap Filling in High-Resolution Mass Spectrometry Data Processing. *Anal. Chem.* **2020**, *92* (15), 10425-10432. DOI: 10.1021/acs.analchem.0c00899.

(2) Wolfender, J.-L.; Nuzillard, J.-M.; van der Hooft, J. J. J.; Renault, J.-H.; Bertrand, S. Accelerating Metabolite Identification in Natural Product Research: Toward an Ideal Combination of Liquid Chromatography–High-Resolution Tandem Mass Spectrometry and NMR Profiling, in Silico Databases, and Chemometrics. *Anal. Chem.* **2019**, *91* (1), 704-742. DOI: 10.1021/acs.analchem.8b05112.

(3) EuropeanCommission. Decision EC 657/2002 of 12 August 2002 implementing Council Directive 96/23/EC concerning the performance of analytical methods and the interpretation of results. **2002**, *Vol. L221*, 8-36.

(4) Haddad, S. P.; Bobbitt, J. M.; Taylor, R. B.; Lovin, L. M.; Conkle, J. L.; Chambliss, C. K.; Brooks, B. W. Determination of microcystins, nodularin, anatoxin-a, cylindrospermopsin, and saxitoxin in water and fish tissue using isotope dilution liquid chromatography tandem mass spectrometry. *J. Chromatogr. A* **2019**, *1599*, 66-74. DOI: 10.1016/j.chroma.2019.03.066.

(5) Kachman, M.; Habra, H.; Duren, W.; Wigginton, J.; Sajjakulnukit, P.; Michailidis, G.; Burant, C.; Karnovsky, A. Deep annotation of untargeted LC-MS metabolomics data with Binner. *Bioinformatics* **2020**, *36* (6), 1801-1806. DOI: 10.1093/bioinformatics/btz798.

(6) Alseekh, S.; Aharoni, A.; Brotman, Y.; Contrepois, K.; D'Auria, J.; Ewald, J.; J, C. E.; Fraser, P. D.; Giavalisco, P.; Hall, R. D.; et al. Mass spectrometry-based metabolomics: a guide for annotation, quantification and best reporting practices. *Nat. Methods* **2021**, *18* (7), 747-756. DOI: 10.1038/s41592-021-01197-1.

(7) De Vijlder, T.; Valkenborg, D.; Lemiere, F.; Romijn, E. P.; Laukens, K.; Cuyckens, F. A tutorial in small molecule identification via electrospray ionization-mass spectrometry: The practical art of structural elucidation. *Mass Spectrom. Rev.* **2018**, *37* (5), 607-629. DOI: 10.1002/mas.21551.

(8) Hohrenk, L. L.; Itzel, F.; Baetz, N.; Tuerk, J.; Vosough, M.; Schmidt, T. C. Comparison of Software Tools for Liquid Chromatography-High-Resolution Mass Spectrometry Data Processing in Nontarget Screening of Environmental Samples. *Anal. Chem.* **2020**, *92* (2), 1898-1907, 10.1021/acs.analchem.9b04095. DOI: 10.1021/acs.analchem.9b04095.

(9) Pezzatti, J.; Boccard, J.; Codesido, S.; Gagnebin, Y.; Joshi, A.; Picard, D.; Gonzalez-Ruiz, V.; Rudaz, S. Implementation of liquid chromatography-high resolution mass spectrometry methods for untargeted metabolomic analyses of biological samples: A tutorial. *Anal. Chim. Acta* **2020**, *1105*, 28-44. DOI: 10.1016/j.aca.2019.12.062.

(10) Kessner, D.; Chambers, M.; Burke, R.; Agus, D.; Mallick, P. ProteoWizard: open source software for rapid proteomics tools development. *Bioinformatics* **2008**, *24* (21), 2534-2536. DOI: 10.1093/bioinformatics/btn323.

(11) Pluskal, T.; Castillo, S.; Villar-Briones, A.; Oresic, M. MZmine 2: modular framework for processing, visualizing, and analyzing mass spectrometry-based molecular profile data. *BMC Bioinf.* **2010**, *11*, 395. DOI: doi.org/10.1186/1471-2105-11-395.

(12) Myers, O. D.; Sumner, S. J.; Li, S.; Barnes, S.; Du, X. One Step Forward for Reducing False Positive and False Negative Compound Identifications from Mass Spectrometry Metabolomics Data: New Algorithms for Constructing Extracted Ion Chromatograms and Detecting Chromatographic Peaks. *Anal. Chem.* **2017**, *89* (17), 8696-8703. DOI: 10.1021/acs.analchem.7b00947.

(13) Kuhl, C.; Tautenhahn, R.; Boettcher, C.; Larson, T. R.; Neumann, S. CAMERA: An Integrated Strategy for Compound Spectra Extraction and Annotation of Liquid Chromatography/Mass Spectrometry Data Sets. *Anal. Chem.* **2012**, *84* (1), 283-289. DOI: 10.1021/ac202450g.

(14) Fraisier-Vannier, O.; Chervin, J.; Cabanac, G.; Puech, V.; Fournier, S.; Durand, V.; Amiel, A.; André, O.; Benamar, O. A.; Dumas, B.; et al. MS-CleanR: A Feature-Filtering Workflow for Untargeted LC–MS Based Metabolomics. *Anal. Chem.* **2020**, *92* (14), 9971-9981. DOI: 10.1021/acs.analchem.0c01594.

(15) Chambers, M. C.; Maclean, B.; Burke, R.; Amodei, D.; Ruderman, D. L.; Neumann, S.; Gatto, L.; Fischer, B.; Pratt, B.; Egertson, J.; et al. A cross-platform toolkit for mass spectrometry and proteomics. *Nat. Biotechnol.* **2012**, *30* (10), 918-920. DOI: 10.1038/nbt.2377.

(16) Nothias, L. F.; Petras, D.; Schmid, R.; Duhrkop, K.; Rainer, J.; Sarvepalli, A.; Protsyuk, I.; Ernst, M.; Tsugawa, H.; Fleischauer, M.; et al. Feature-based molecular networking in the GNPS analysis environment. *Nat. Methods* **2020**, *17* (9), 905-908. DOI: 10.1038/s41592-020-0933-6.

(17) Wang, M.; Carver, J. J.; Phelan, V. V.; Sanchez, L. M.; Garg, N.; Peng, Y.; Nguyen, D. D.; Watrous, J.; Kapono, C. A.; Luzzatto-Knaan, T.; et al. Sharing and community curation of mass spectrometry data with Global Natural Products Social Molecular Networking. *Nat. Biotechnol.* **2016**, *34* (8), 828-837. DOI: 10.1038/nbt.3597.

(18) Shannon, P.; Markiel, A.; Ozier, O.; Baliga, N. S.; Wang, J. T.; Ramage, D.; Amin, N.; Schwikowski, B.; Ideker, T. Cytoscape: a software environment for integrated models of biomolecular interaction networks. *Genome Res.* **2003**, *13* (11), 2498-2504. DOI: 10.1101/gr.1239303.

(19) Cao, L.; Guler, M.; Tagirdzhanov, A.; Lee, Y. Y.; Gurevich, A.; Mohimani, H. MolDiscovery: learning mass spectrometry fragmentation of small molecules. *Nat Commun* **2021**, *12* (1), 3718. DOI: 10.1038/s41467-021-23986-0.

(20) Guijas, C.; Montenegro-Burke, J. R.; Domingo-Almenara, X.; Palermo, A.; Warth, B.; Hermann, G.; Koellensperger, G.; Huan, T.; Uritboonthai, W.; Aisporna, A. E.; et al. METLIN: A Technology Platform for Identifying Knowns and Unknowns. *Anal. Chem.* **2018**, *90* (5), 3156-3164. DOI: 10.1021/acs.analchem.7b04424.

(21) Horai, H.; Arita, M.; Kanaya, S.; Nihei, Y.; Ikeda, T.; Suwa, K.; Ojima, Y.; Tanaka, K.; Tanaka, S.; Aoshima, K. MassBank: a public repository for sharing mass spectral data for life sciences. *J. Mass Spectrom.* **2010**, *45*. DOI: 10.1002/jms.1777.

(22) Wishart, D. S.; Feunang, Y. D.; Marcu, A.; Guo, A. C.; Liang, K.; Vazquez-Fresno, R.; Sajed, T.; Johnson, D.; Li, C.; Karu, N.; et al. HMDB 4.0: the human metabolome database for 2018. *Nucleic Acids Res.* **2018**, *46* (D1), D608-D617. DOI: 10.1093/nar/gkx1089.

(23) Musharraf, S. G.; Ali, A.; Ali, R. A.; Yousuf, S.; Rahman, A. U.; Choudhary, M. I. Analysis and development of structure-fragmentation relationships in withanolides using an electrospray ionization quadropole time-of-flight tandem mass spectrometry hybrid instrument. *Rapid Commun. Mass Spectrom.* **2011**, *25* (1), 104-114. DOI: 10.1002/rcm.4835.

(24) Tetali, S. D.; Acharya, S.; Ankari, A. B.; Nanakram, V.; Raghavendra, A. S. Metabolomics of Withania somnifera (L.) Dunal: Advances and applications. *J. Ethnopharmacol.* **2021**, *267*, 113469. DOI: 10.1016/j.jep.2020.113469.

(25) Trivedi, M. K.; Panda, P.; Sethi, K. K.; Jana, S. Metabolite Profiling in Withania somnifera Roots Hydroalcoholic Extract Using LC/MS, GC/MS and NMR Spectroscopy. *Chem. Biodivers.* **2017**, *14* (3). DOI: 10.1002/cbdv.201600280.

(26) Girme, A.; Saste, G.; Pawar, S.; Balasubramaniam, A. K.; Musande, K.; Darji, B.; Satti, N. K.; Verma, M. K.; Anand, R.; Singh, R.; et al. Investigating 11 Withanosides and Withanolides by UHPLC-PDA and Mass Fragmentation Studies from Ashwagandha (Withania somnifera). *ACS Omega* **2020**, *5* (43), 27933-27943. DOI: 10.1021/acsomega.0c03266.

(27) Khajuria, R. K.; Suri, K. A.; Gupta, R. K.; Satti, N. K.; Amina, M.; Suri, O. P.; Qazi, G. N. Separation, identification, and quantification of selected withanolides in plant extracts of Withania somnifera by HPLC-UV(DAD)--positive ion electrospray ionisation-mass spectrometry. *J. Sep. Sci.* **2004**, *27* (7-8), 541-546. DOI: 10.1002/jssc.200301690.

(28) Rutz, A.; Sorokina, M.; Galgonek, J.; Mietchen, D.; Willighagen, E.; Gaudry, A.; Graham, J. G.; Stephan, R.; Page, R.; Vondrasek, J.; et al. The LOTUS initiative for open knowledge management in natural products research. *Elife* **2022**, *11*. DOI: 10.7554/eLife.70780.
(29) Alcazar Magana, A.; Wright, K.; Vaswani, A.; Caruso, M.; Reed, R. L.; Bailey, C. F.; Nguyen, T.; Gray, N. E.; Soumyanath, A.; Quinn, J.; et al. Integration of mass spectral fingerprinting analysis with precursor ion (MS1) quantification for the characterisation of botanical extracts: application to extracts of Centella asiatica (L.) Urban. *Phytochem. Anal.* **2020**, Ahead of Print, 10.1002/pca.2936. DOI: 10.1002/pca.2936.
(30) Sumner, L. W.; Amberg, A.; Barrett, D.; Beale, M. H.; Beger, R.; Daykin, C. A.; Fan, T. W. M.; Fiehn, O.; Goodacre, R.; Griffin, J. L.; et al. Proposed minimum reporting standards for chemical analysis. Chemical Analysis Working Group (CAWG) Metabolomics Standards Initiative (MSI). *Metabolomics* **2007**, *3* (3), 211-221. DOI: 10.1007/s11306-007-0082-2.
(31) Tsugawa, H.; Cajka, T.; Kind, T.; Ma, Y.; Higgins, B.; Ikeda, K.; Kanazawa, M.; VanderGheynst, J.; Fiehn, O.; Arita, M. MS-DIAL: data-independent MS/MS deconvolution for comprehensive metabolome analysis. *Nat. Methods* **2015**, *12* (6), 523-526. DOI: 10.1038/nmeth.3393.
(32) Tsugawa, H.; Kind, T.; Nakabayashi, R.; Yukihira, D.; Tanaka, W.; Cajka, T.; Saito, K.; Fiehn, O.; Arita, M. Hydrogen rearrangement rules: computational MS/MS fragmentation and structure elucidation using MS-FINDER software. *Anal. Chem.* **2016**, *88*. DOI: 10.1021/acs.analchem.6b00770.
(33) Olivier-Jimenez, D.; Bouchouireb, Z.; Ollivier, S.; Mocquard, J.; Allard, P.-M.; Bernadat, G.; Chollet-Krugler, M.; Rondeau, D.; Boustie, J.; van der Hooft, J. J. J.; et al. From mass spectral features to molecules in molecular networks: a novel workflow for untargeted metabolomics. **2021**, BioRxiv [Preprint] December 22, 2021. DOI: Available from: 10.1101/2021.12.21.473622.
(34) de Jonge, N. F.; Louwen, J. J. R.; Chekmeneva, E.; Camuzeaux, S.; Vermeir, F. J.; Jansen, R. S.; Huber, F.; van der Hooft, J. J. J. MS2Query: reliable and scalable MS(2) mass spectra-based analogue search. *Nat. Commun.* **2023**, *14* (1), 1752. DOI: 10.1038/s41467-023-37446-4.
(35) Huber, F.; van der Burg, S.; van der Hooft, J. J. J.; Ridder, L. MS2DeepScore: a novel deep learning similarity measure to compare tandem mass spectra. *J. Cheminform.* **2021**, *13* (1), 84. DOI: 10.1186/s13321-021-00558-4.
(36) Huber, F.; Ridder, L.; Verhoeven, S.; Spaaks, J. H.; Diblen, F.; Rogers, S.; van der Hooft, J. J. J. Spec2Vec: Improved mass spectral similarity scoring through learning of structural relationships. *PLoS Comput. Biol.* **2021**, *17* (2), e1008724. DOI: 10.1371/journal.pcbi.1008724.
(37) Bertrand, S.; Guitton, Y.; Roullier, C. Successes and pitfalls in automated dereplication strategy using liquid chromatography coupled to mass spectrometry data: A CASMI 2016 experience. *Phytochem. Lett.* **2017**, *21*, 297-305. DOI: 10.1016/j.phytol.2016.12.025.
(38) Neumann, S.; Nikolic, D.; Schymanski, E.; Shahaf, N. Critical Assessment of Small Molecule Identification: Looking at the 5th Edition of CASMI. *MetaboNews* **2018**, *8* (3), 5-8.
(39) R Core Team. R: A language and environment for statistical computing. *R Foundation for Statistical Computing, Vienna, Austria.* **2015**, URL http://www.R-project.org/.
(40) Tautenhahn, R.; Bottcher, C.; Neumann, S. Highly sensitive feature detection for high resolution LC/MS. *BMC Bioinformatics* **2008**, *9*, 504. DOI: 10.1186/1471-2105-9-504.
(41) Gatto, L.; Gibb, S.; Rainer, J. MSnbase, Efficient and Elegant R-Based Processing and Visualization of Raw Mass Spectrometry Data. *Journal of Proteome Research* **2021**, *20* (1), 1063-1069. DOI: 10.1021/acs.jproteome.0c00313.
(42) Kuhl, C.; Tautenhahn, R.; Böttcher, C.; Larson, T. R.; Neumann, S. CAMERA: An Integrated Strategy for Compound Spectra Extraction and Annotation of Liquid Chromatography/Mass Spectrometry Data Sets. *Analytical chemistry* **2012**, *84* (1), 283-289. DOI: 10.1021/ac202450g (acccessed 2013/10/28).
(43) Libiseller, G.; Dvorzak, M.; Kleb, U.; Gander, E.; Eisenberg, T.; Madeo, F.; Neumann, S.; Trausinger, G.; Sinner, F.; Pieber, T.; et al. IPO: a tool for automated optimization of XCMS parameters. *BMC Bioinf.* **2015**, *16*, 118. DOI: 10.1186/s12859-015-0562-8.
(44) Roullier, C.; Guitton, Y.; Valery, M.; Amand, S.; Prado, S.; Robiou du Pont, T.; Grovel, O.; Pouchus, Y. F. Automated detection of natural halogenated compounds from LC-MS profiles – Application to the isolation of bioactive chlorinated compounds from marine-derived fungi. *Analytical chemistry* **2016**, *88* (18), 9143-9150. DOI: 10.1021/acs.analchem.6b02128.
(45) Stravs, M. A.; Schymanski, E. L.; Singer, H. P.; Hollender, J. Automatic recalibration and processing of tandem mass spectra using formula annotation. *Journal of Mass Spectrometry* **2013**, *48* (1), 89-99 (RMassBank package available from http://bioconductor.org/packages/RMassBank/). DOI: 10.1002/jms.3131.
(46) Chamberlain, S.; Szöcs, E. Taxize: Taxonomic search and retrieval in R. *F1000Research* **2013**, *2*, 191. DOI: 10.12688/f1000research.2-191.v2.
(47) Böcker, S.; Letzel, M. C.; Lipták, Z.; Pervukhin, A. SIRIUS: decomposing isotope patterns for metabolite identification. *Bioinformatics* **2009**, *25* (2), 218-224. DOI: 10.1093/bioinformatics/btn603.
(48) Shafee , T.; Keyes , O.; Signorelli, S. WikidataR: Read-Write API Client Library for Wikidata. **2021**, https://CRAN.R-project.org/package=WikidataR.
(49) Chamberlain, S.; Welty, E. wikitaxa: Taxonomic Information from 'Wikipedia'. **2020**, https://CRAN.R-project.org/package=wikitaxa.
(50) Chapman; Hall. *Dictionary of Natural Products on DVD (23:1)*; CRC Press, Taylor & Francis Group, URL: http://dnp.chemnetbase.com/, 2014.
(51) Kind, T.; Fiehn, O. Seven golden rules for heuristic filtering of molecular formulas obtained by accurate mass spectrometry. *BMC Bioinformatics* **2007**, *8*, 105. DOI: 10.1186/1471-2105-8-105.
(52) Meusel, M.; Hufsky, F.; Panter, F.; Krug, D.; Müller, R.; Böcker, S. Predicting the presence of uncommon elements in unknown biomolecules from isotope patterns. *Analytical chemistry* **2016**, *88* (15), 7556-7566. DOI: 10.1021/acs.analchem.6b01015.
(53) Bertrand, S.; Roullier, C.; Guitton, Y. Successes and Pitfalls in Automated Dereplication Strategy using Mass Spectrometry Data: a CASMI Experience. *Current Metabolomics* **2017**, DOI: 10.2174/2213235X04666160622074357.
(54) Wolfender, J.-L.; Nuzillard, J.-M.; van der Hooft, J. J. J.; Renault, J.-H.; Bertrand, S. Accelerating metabolite identification in natural product research: toward an ideal combination of LC-HRMS/MS and NMR profiling, *in silico* databases and chemometrics. *Analytical chemistry* **2019**, *91* (1), 704-742. DOI: 10.1021/acs.analchem.8b05112.
(55) Wang, M.; Carver, J.; Phelan, V.; Sanchez, L.; Garg, N.; Peng, Y.; Nguyen, D.; Watrous, J.; Kapono, C.; Luzzatto Knaan, T.; et al. Sharing and community curation of mass spectrometry data with Global Natural Products Social Molecular Networking. *Nature biotechnology* **2016**, *34*, 828-837. DOI: 10.1038/nbt.3597.
(56) O'Boyle, N.; Banck, M.; James, C.; Morley, C.; Vandermeersch, T.; Hutchison, G. Open Babel: An open chemical toolbox. *Journal of Cheminformatics* **2011**, *3*, 33. DOI: 10.1186/1758-2946-3-33.
(57) Wang, F.; Liigand, J.; Tian, S.; Arndt, D.; Greiner, R.; Wishart, D. S. CFM-ID 4.0: More Accurate ESI-MS/MS Spectral Prediction and Compound Identification. *Analytical chemistry* **2021**. DOI: 10.1021/acs.analchem.1c01465.
(58) Wang, M.; Bandeira, N. Spectral library generating function for assessing spectrum-spectrum match significance. *Journal of Proteome Research* **2013**, *12* (9), 3944-3951. DOI: 10.1021/pr400230p.

(59) Wang, M.; Carver, J. J.; Phelan, V. V.; Sanchez, L. M.; Garg, N.; Peng, Y.; Nguyen, D. D.; Watrous, J.; Kapono, C. A.; Luzzatto-Knaan, T.; et al. Sharing and community curation of mass spectrometry data with Global Natural Products Social Molecular Networking. *Nat. Biotechnol.* **2016**, *34* (8), 828-837. DOI: 10.1038/nbt.3597.
(60) Nothias, L. F.; Petras, D.; Schmid, R.; Duhrkop, K.; Rainer, J.; Sarvepalli, A.; Protsyuk, I.; Ernst, M.; Tsugawa, H.; Fleischauer, M.; et al. Feature-based molecular networking in the GNPS analysis environment. *Nat Methods* **2020**, *17* (9), 905-908. DOI: 10.1038/s41592-020-0933-6.
(61) Dührkop, K.; Fleischauer, M.; Ludwig, M.; Aksenov, A. A.; Melnik, A. V.; Meusel, M.; Dorrestein, P. C.; Rousu, J.; Böcker, S. SIRIUS 4: a rapid tool for turning tandem mass spectra into metabolite structure information. *Nature Methods* **2019**, *16* (4), 299-302. DOI: 10.1038/s41592-019-0344-8.
(62) Horai, H.; Arita, M.; Kanaya, S.; Nihei, Y.; Ikeda, T.; Suwa, K.; Ojima, Y.; Tanaka, K.; Tanaka, S.; Aoshima, K.; et al. MassBank: a public repository for sharing mass spectral data for life sciences. *J. Mass Spectrom.* **2010**, *45* (7), 703-714. DOI: 10.1002/jms.1777.
(63) Mohimani, H.; Gurevich, A.; Shlemov, A.; Mikheenko, A.; Korobeynikov, A.; Cao, L.; Shcherbin, E.; Nothias, L.-F.; Dorrestein, P. C.; Pevzner, P. A. Dereplication of microbial metabolites through database search of mass spectra. *Nature Communications* **2018**, *9* (1). DOI: 10.1038/s41467-018-06082-8.
(64) Böcker, S.; Dührkop, K. Fragmentation trees reloaded. *Journal of Cheminformatics* **2016**, *8* (1), https://doi.org/10.1186/s13321-13016-10116-13328, [Online]. DOI: 10.1186/s13321-016-0116-8.
(65) Ludwig, M.; Nothias, L.-F.; Dührkop, K.; Koester, I.; Fleischauer, M.; Hoffmann, M. A.; Petras, D.; Vargas, F.; Morsy, M.; Aluwihare, L.; et al. Database-independent molecular formula annotation using Gibbs sampling through ZODIAC. *Nature Machine Intelligence* **2020**, *2* (10), 629-641. DOI: 10.1038/s42256-020-00234-6.
(66) Hoffmann, M. A.; Nothias, L.-F.; Ludwig, M.; Fleischauer, M.; Gentry, E. C.; Witting, M.; Dorrestein, P. C.; Dührkop, K.; Böcker, S. High-confidence structural annotation of metabolites absent from spectral libraries. *Nat. Biotechnol.* **2021**, *40* (3), 411-421. DOI: 10.1038/s41587-021-01045-9.
(67) Dührkop, K.; Shen, H.; Meusel, M.; Rousu, J.; Böcker, S. Searching molecular structure databases with tandem mass spectra using CSI:FingerID. *Proceedings of the National Academy of Sciences* **2015**, *112* (41), 12580-12585. DOI: 10.1073/pnas.1509788112.
(68) Kim, H. W.; Wang, M.; Leber, C. A.; Nothias, L.-F.; Reher, R.; Kang, K. B.; van der Hooft, J. J. J.; Dorrestein, P. C.; Gerwick, W. H.; Cottrell, G. W. NPClassifier: A Deep Neural Network-Based Structural Classification Tool for Natural Products. *J. Nat. Prod.* **2021**, *84* (11), 2795-2807. DOI: 10.1021/acs.jnatprod.1c00399.
(69) Dührkop, K.; Nothias, L.-F.; Fleischauer, M.; Reher, R.; Ludwig, M.; Hoffmann, M. A.; Petras, D.; Gerwick, W. H.; Rousu, J.; Dorrestein, P. C.; et al. Systematic classification of unknown metabolites using high-resolution fragmentation mass spectra. *Nat. Biotechnol.* **2020**, *39* (4), 462-471. DOI: 10.1038/s41587-020-0740-8.
(70) Djoumbou Feunang, Y.; Eisner, R.; Knox, C.; Chepelev, L.; Hastings, J.; Owen, G.; Fahy, E.; Steinbeck, C.; Subramanian, S.; Bolton, E.; et al. ClassyFire: automated chemical classification with a comprehensive, computable taxonomy. *Journal of Cheminformatics* **2016**, *8* (1), https://doi.org/10.1186/s13321-13016-10174-y, [Online]. DOI: 10.1186/s13321-016-0174-y.
(71) Shannon, P.; Markiel, A.; Ozier, O.; Baliga, N. S.; Wang, J. T.; Ramage, D.; Amin, N.; Schwikowski, B.; Ideker, T. Cytoscape: A Software Environment for Integrated Models of Biomolecular Interaction Networks. *Genome Research* **2003**, *13* (11), 2498-2504. DOI: 10.1101/gr.1239303.
(72) Houriet, J.; Vidar, W. S.; Manwill, P. K.; Todd, D. A.; Cech, N. B. How Low Can You Go? Selecting Intensity Thresholds for Untargeted Metabolomics Data Preprocessing. *Anal. Chem.* **2022**, *94* (51), 17964-17971. DOI: 10.1021/acs.analchem.2c04088.
(73) Sumner, L. W.; Amberg, A.; Barrett, D.; Beale, M. H.; Beger, R.; Daykin, C. A.; Fan, T. W. M.; Fiehn, O.; Goodacre, R.; Griffin, J. L.; et al. Proposed minimum reporting standards for chemical analysis. *Metabolomics* **2007**, *3* (3), 211-221. DOI: 10.1007/s11306-007-0082-2.
(74) Schymanski, E. L.; Jeon, J.; Gulde, R.; Fenner, K.; Ruff, M.; Singer, H. P.; Hollender, J. Identifying Small Molecules via High Resolution Mass Spectrometry: Communicating Confidence. *Environ. Sci. Technol.* **2014**, *48* (4), 2097-2098. DOI: 10.1021/es5002105.
(75) Nakamura, Y. *KNApSAcK: a comprehensive species-metabolite relationship database*. 2023. http://www.knapsackfamily.com/KNApSAcK/ (accessed 12.05.2023).
(76) Duhrkop, K.; Fleischauer, M.; Ludwig, M.; Aksenov, A. A.; Melnik, A. V.; Meusel, M.; Dorrestein, P. C.; Rousu, J.; Bocker, S. SIRIUS 4: a rapid tool for turning tandem mass spectra into metabolite structure information. *Nat. Methods* **2019**, *16* (4), 299-302. DOI: 10.1038/s41592-019-0344-8.
