# Supplementary material for: Multilaboratory Untargeted Mass Spectrometry Metabolomics Collaboration to Identify Bottlenecks and Comprehensively Annotate A Single Dataset
Source: Anal Chem. 2025 Jul 22;97(30):16110–22. doi: 10.1021/acs.analchem.4c05577 (PMC12332825; doi:10.1021/acs.analchem.4c05577)
Supplement: Supplementary file 2 [file ac4c05577_si_002.pdf]

Supporting information

A multi-laboratory untargeted mass spectrometry metabolomics collaboration to identify bottlenecks and comprehensively annotate a single dataset

Joelle Houriet[1‡], Preston K. Manwill[1‡], Armando Alcázar Magaña[2], Victoria M. Anderson[1], Mehdi A. Beniddir[3], Samuel Bertrand[4, 5], Jaewoo Choi[6], Trevor N. Clark[7], Leonard Foster[2], Maria Halabalaki[8], Alan K. Jarmusch[9], Niek F. de Jonge[10], Aswad Khadilkar[11], John B. MacMillan[11], Claudia S. Maier[6], Luke C. Marney[6], , Guillaume Marti[12, 13], Eleni V. Mikropoulou[8], Damien Olivier-Jimenez[14], Amélie Perez[12, 13], Justin J. J. van der Hooft[10, 15], Mitja M. Zdouc[10], Roger G. Linington[7], Nadja B. Cech[1]*

‡ co-first authors

[1] Department of Chemistry & Biochemistry, University of North Carolina at Greensboro, Greensboro, North Carolina 27402, USA [2] Life Sciences Institute, Department of Biochemistry and Molecular Biology, University of British Columbia, Vancouver BC, Canada, V6T 1Z4

[3] Équipe "Chimie des substances naturelles" BioCIS, CNRS, Université Paris-Saclay, 17, avenue des Sciences, 91400, Orsay, France

[4] Nantes Université, Institut des Substances et Organismes de la Mer, ISOMER, UR 2160, 44000 Nantes, France,

[5] Nantes Université, École Centrale Nantes, CNRS, LS2N, UMR 6004, 44000 Nantes, France

[6] Department of Chemistry, Oregon State University, Oregon 97331, USA

[7] Department of Chemistry, Simon Fraser University, Burnaby, BC V5A 4Y8, Canada

[8] Division of Pharmacognosy and Natural Products Chemistry, Department of Pharmacy, National and Kapodistrian University of Athens, 157 71 Zographou, Greece

[9] Immunity, Inflammation, and Disease Laboratory, Division of Intramural Research, National Institute of Environmental Health Sciences, National Institutes of Health, Research Triangle Park, NC 27709, USA

[10] Bioinformatics Group, Wageningen University & Research, Wageningen, 6708 PB, the Netherlands

[11] Department of Chemistry & Biochemistry, University of California Santa Cruz, Santa Cruz, California 95064, USA

[12] Laboratoire de Recherche en Sciences Végétales, Metatoul-AgromiX Platform, Université de Toulouse, CNRS, INP, , 31320, Auzeville-Tolosane, France

[13] MetaboHUB-MetaToul, National Infrastructure of Metabolomics and Fluxomics, Toulouse 31400, France

[14] Center for Proteomics and Metabolomics, Leiden University Medical Center, 2333 ZA Leiden, the Netherlands

[15] Department of Biochemistry, University of Johannesburg, Johannesburg 2006, South Africa

*Email: nadja_cech@uncg.edu

KEYWORDS: metabolite annotation, MS[1] in-source features, mass spectrometry metabolomics, preprocessing, Withania somnifera, comparative study

Contents

About the Mass Spectrometry Metabolomics Annotation Collaboration.. S4
Instructions for Data Analysis: Metabolomics Annotation Collaboration... S7
Description of the Dataset.. S14
Links to Mass Spectrometry Evaluation Data... S15
Example Dataset for your Internal Benchmarking... S22

Tables

Table S1. *Camellia sinensis* (CS) reference compounds included in CS standard mixture (CSSM)................... S22

Figures

Figure S1. Heatmap comparing results of the pilot version of this study.. S6

Figure S2. Example of completed .csv file for DDA analysis of green tea (*Camellia sinensis*) extract with a Q-ToF in DDA positive ion mode from Laboratory A.. S10

Figure S3. Example of completed .csv file for DDA analysis of green tea (*Camellia sinensis*) extract with a Q-ToF in DDA positive ion mode from Laboratory B.. S11

Figure S4. An example of how to set up your FTP client to access the MassIVE datasets................................ S15

ABOUT THE MASS SPECTROMETRY METABOLOMICS ANNOTATION COLLABORATION

The landscape of data analysis tools available to researchers doing mass spectrometry metabolomics is complex and ever-changing. To identify effective approaches within this complex landscape, and to find and address existing hurdles in the field, we seek to undertake a practical exercise in mass spectrometry dataset annotation. This project springs from efforts of the NIH-Funded *Center for High Content Functional Annotation of Natural Products* (HiFAN), which seeks to create tools that will enable effective research in the field of natural products. To carry out the "Mass Spectrometry Metabolomics Annotation Collaboration" Dr. Nadja Cech at the University of North Carolina Greensboro and Dr. Roger Linington at Simon Fraser University are inviting a select group of mass spectrometry metabolomics experts to analyze the same dataset, with the goal of annotating as many **analytes** as possible. Here we define "analyte" as a single chemical entity (molecule) present in the sample being analyzed. In a typical LC-MS metabolomics acquisition, multiple features (ions) may be detected for each individual analyte, including the protonated molecule ($[M+H]^+$), salt or solvent clusters, in-source fragments, and isotopes. We are seeking your help to identify effective strategies to reduce this complex set of features down to a simpler set of analytes.

After all participants have had time to evaluate the datasets provided, researchers in the Cech Laboratory will compare the results from each of the participating laboratories and share them with all participants. Our goal is to publish a paper (Target Journals *Analytical Chemistry* or *Journal of Natural Products*) that describes the findings of this collaborative exercise and makes recommendations to others who might seek to effectively annotate metabolomics datasets. Everyone who participates in this project will, of course, be invited (but not obligated) to contribute data and serve as coauthors on this paper.

Proposed Outcomes and Benefits to the Community:

- Annotation guidelines and recommended approaches for the analysis of a botanical natural product extract (to be communicated through a peer-reviewed publication)
- Consensus list of analytes present in a single analysis of a botanical extract (annotations linked to open access datafiles)
  - Useful for future benchmarking studies and tool development
  - Useful to answer the question, "How many chemical entities are there in one botanical extract?

What we're inviting participants to do:

Many participants participated in the orientation/Q&A Call on March 2nd (again thank you all!). For those who missed the call and wish to review it, a video is available here.

https://uncg.zoom.us/rec/share/zopHFlg0UNcg9U_2zxb_SRqMMJQ_ZIizU56Lw4ORebEWOPO6l5bHV_FqSE4byYCU.SXlmNdQP-GOWYKIG?startTime=1646236715000 (Passcode: *%.C7=Rg)

Moving forward, here are the recommendations we have for this collaboration, which are based on our own experience running the pilot test, and on suggestions/discussion during the March 2nd call.

- Select at least one positive ion mode dataset from the various instrument types and acquisition modes available and analyze it with the goal of annotating (with specified levels of confidence) as many analytes as possible. If desired, analyze the corresponding negative ion mode data as well.
- Write a paragraph that describes your approach to data analysis, including methods and parameters for data processing (background subtraction, peak picking, alignment, filtering, etc.) and any tools, databases, or other references used to assign structures. This paragraph will be submitted to us as a .docx file and will serve as a starting point for the methods section of the research publication (should you choose to participate in that portion).
- Complete a survey that will capture the methods and parameters used by each group for data analysis. At the end of the survey, you will be asked to submit a .csv file (template and examples are provided) with information about the analytes identified in the dataset as well as a .docx file with methods used for the analysis and

annotation (example provided). If you choose to analyze more than one dataset (i.e., negative and positive ion mode data, or data from both Q-ToF and Orbi), you can repeat the data analysis process and submit a separate survey/.csv file/.docx file.

- Participate (if possible) in a follow up discussion by Zoom (planned for early May 2022) where we will share the results of the analyses conducted by different laboratories and discuss their relevance to the field of mass spectrometry metabolomics.
- Optional: Participate as a coauthor and data contributor to a publication that describes the results of the collaborative exercise.

What is the time commitment?

To test this project, we tried a pilot version in house (using an extract from green tea). We've given everyone access to those data files so that you can use them for your own benchmarking. In our pilot trial, the data analysis portion of this exercise took approximately 20-30 hours of time each for two postdocs, one in the Cech Laboratory and one in the Linington Laboratory.

How will we evaluate and compare the results from the different laboratories?

- Create workflow figures for each laboratory comparing the different approaches to annotation of the mass spectrometry metabolomics data.
- Compare survey results to capture different approaches used for data analysis.
- Check the analytes reported by each laboratory against a list of ten analytes known to have been detected in each LC-MS run (based on analysis of authentic standards). These standards are only a small subset of the analytes present in each sample, however, comparison against standards will provide an opportunity to check for false negatives.
- Compare numbers of features and numbers of analytes reported across laboratories.
- Create a set of heatmaps comparing the analytes reported across laboratories (by mass and retention time) and their proposed identities. This heatmap will be the first step towards developing the "consensus list" of analytes detected in the sample (**Figure S1**).
- Compare the level of confidence (using a set of annotation guidelines that will be provided) assigned to various analytes across laboratories).
- Create a set of figures from the collective results of the study, to be included in the research publication.
- Have follow-up conversations with the various research groups to discuss and interpret results and understand different approaches to dataset annotation.
- Possibly conduct additional structural confirmation of the consensus list of analytes by purchasing additional standards and analyzing them under the same LC-MS conditions to compare retention time and MS-MS fragmentation data.

We don't have a "right answer" in mind for this collaborative undertaking. We hope to use the exercise as a jumping off point to understand how leaders in the field are addressing the problem of reducing a set of features detected by LC-MS to a list of identified analytes. As we compare the different approaches used towards this goal, we expect that many questions will come up. Is it better to identify a very long list of analytes, or just focus on the ones detected with the highest abundance or the highest level of confidence? How useful is it to dig deeply into the data and annotate minor constituents? Are the analytes that are identified by only one laboratory "real"? What constitutes a false positive? We look forward to discussing these and many more questions with you, the experts, in the context of this undertaking, with the expectation that our conversations will ultimately prove useful to the community of scientists using mass spectrometry to characterize complex mixtures.

<table>
<tr><td>Annotation Level Color Code</td></tr>
<tr><td>Level 2</td></tr>
<tr><td>Level 3</td></tr>
<tr><td>Level 4</td></tr>
</table>

<table>
<tr><th>Standard/Analyte</th><th>Analyte RT (min)</th><th>Exact mass (Da)</th><th>Detected m/z</th><th>Laboratory A</th><th>Laboratory B</th></tr>
<tr><td>theanine, L-</td><td>0.51</td><td>174.10044</td><td>175.1078</td><td></td><td>theanine, L-</td></tr>
<tr><td>gallocatechin</td><td>1.55</td><td>306.07395</td><td>307.0810|307.0808</td><td>gallocatechin</td><td>gallocatechin|epigallocatechin</td></tr>
<tr><td>epigallocatechin</td><td>1.94|1.92</td><td>306.07395</td><td>307.0810|307.0810</td><td>epigallocatechin</td><td>epigallocatechin|gallocatechin</td></tr>
<tr><td>catechin</td><td>2.1|2.11</td><td>290.07904</td><td>291.0850|291.0860</td><td>catechin</td><td>catechin|epicatechin</td></tr>
<tr><td>caffeine</td><td>2.26|2.20</td><td>194.08038</td><td>195.0886|195.0880</td><td>caffeine</td><td>caffeine</td></tr>
<tr><td>epicatechin</td><td>2.39</td><td>290.079</td><td>291.086</td><td>epicatechin</td><td></td></tr>
<tr><td>epigallocatechin gallate</td><td>2.4</td><td>458.0849</td><td>459.091</td><td>epigallocatechin gallate</td><td></td></tr>
<tr><td>gallocatechin gallate</td><td>2.42</td><td>458.0849</td><td>459.093</td><td>gallocatechin gallate</td><td></td></tr>
<tr><td>rutin</td><td>2.81</td><td>610.1534</td><td>611.161</td><td>rutin</td><td></td></tr>
<tr><td>epicatechin gallate</td><td>2.91|2.92</td><td>442.09</td><td>443.098|443.0976</td><td>epicatechin gallate</td><td>epicatechin gallate|epicatechin-3-O-gallate</td></tr>
<tr><td>1</td><td>0.45</td><td>342.11621</td><td>343.1235</td><td></td><td>sucrose|galactinol</td></tr>
<tr><td>2</td><td>0.75</td><td>174.10044</td><td>175.1075</td><td></td><td>unknown small peptide</td></tr>
<tr><td>3</td><td>1.52</td><td>180.06473</td><td>181.0730|181.0718</td><td>theobromine</td><td>theobromine|theophylline</td></tr>
<tr><td>4</td><td>1.76</td><td>320.05322</td><td>321.0602</td><td></td><td>dihydromyricetin (syn. Ampelopsin)</td></tr>
<tr><td>5</td><td>1.92</td><td>610.13226</td><td>611.1392</td><td></td><td>theasinensin C</td></tr>
<tr><td>6</td><td>2.2</td><td>578.14243</td><td>579.1499</td><td></td><td>proanthocyanidin B2</td></tr>
<tr><td>7</td><td>2.29</td><td>594.1585</td><td>595.164</td><td>nicotiflorin</td><td></td></tr>
<tr><td>8</td><td>2.37</td><td></td><td>595.1659</td><td></td><td>unknown analyte</td></tr>
<tr><td>9</td><td>2.45</td><td>552.0904</td><td>553.03</td><td>Epitheaflagallin 3-O-gallate</td><td></td></tr>
<tr><td>10</td><td>2.48</td><td>286.04774</td><td>287.0548</td><td></td><td>kaempferol|fisetin|luteolin</td></tr>
<tr><td>11</td><td>2.51</td><td></td><td>458.0849</td><td>unknown analyte</td><td></td></tr>
<tr><td>12</td><td>2.53</td><td>564.1479</td><td>565.156</td><td>Vicenin 3</td><td></td></tr>
<tr><td>13</td><td>2.6</td><td>480.09039</td><td>481.0981</td><td></td><td>myricetin-3-O-hexoside|myricetin-3'-O-glucoside</td></tr>
<tr><td>14</td><td>2.62</td><td></td><td>319.045</td><td>unknown analyte</td><td></td></tr>
<tr><td>15</td><td>2.68</td><td>772.20621</td><td>773.2130|773.2141</td><td>unknown analyte</td><td>quercetin-3-O-triglycosides (2 structures for the species)</td></tr>
</table>

Figure S1. Heatmap comparing results of the pilot version of this study using a positive ion mode data dependent MS-MS acquisition of an extract from green tea (*Camelia sinensis*) on a Q-ToF. Results are compared of analytes reported by two postdocs in the Cech and Linington Laboratories analyzing the same dataset. Only a subset of the total of 64 analytes detected are included here for ease of visualization.

INSTRUCTIONS FOR DATA ANALYSIS: METABOLOMICS ANNOTATION COLLABORATION

Guidelines for Completion of the Survey(s)

We have prepared an accompanying survey to 1) gather demographic information regarding the participating laboratories and/or individuals, 2) capture the tools, methods, and parameters used by each group during the crucial data processing steps of metabolomics data analysis, 3) capture the tools, methods, and parameters used for annotation, and 4) capture the output statistics for the metabolomics data processing and annotation.

Please choose at least one positive ion mode data type to analyze (full scan, data dependent, or data independent on either the Q-ToF or Orbitrap). If you wish to analyze a second dataset, you can do so, but you should then submit a separate survey with separate attachments for that second datatype. We understand that workflows and parameters differ between data types and ionization modes, thus we have prepared separate surveys for each acquisition mode and polarity. Please follow the respective links to answer the survey for each data type you choose to analyze.

Before you begin, we encourage you to have your metabolomics software/tools open and the methods/parameters that you implemented nearby.

There are two 'file upload' questions at the end of the survey for you to upload 1) a .csv file containing your list of annotated analytes and 2) a .docx file containing a description of your approach used to analyze the dataset. If you choose to analyze more than one data type, you should prepare a separate .csv file and separate .docx file for each data type that you choose to analyze.

Survey links:

Orbitrap datasets surveys

- *W. somnifera* Orbi_POS_FS (positive mode, full scan)
- *W. somnifera* Orbi_POS_DDA (positive mode, data dependent analysis – MS^2)
- *W. somnifera* Orbi_POS_DIA (positive mode, data independent analysis – AIF)
- *W. somnifera* Orbi_NEG_FS (negative mode, full scan)
- *W. somnifera* Orbi_NEG_DDA (negative mode, data dependent analysis – MS^2)
- *W. somnifera* Orbi_NEG_DIA (negative mode, data independent analysis – AIF)

Q-ToF datasets surveys

- *W. somnifera* QToF_POS_FS (positive mode, full scan)
- *W. somnifera* QToF_POS_DDA (positive mode, data dependent analysis – MS^2)
- *W. somnifera* QToF_POS_DIA (positive mode, data independent analysis – MS^e)
- *W. somnifera* QToF_NEG_FS (negative mode, full scan)
- *W. somnifera* QToF_NEG_DDA (negative mode, data dependent analysis – MS^2)
- *W. somnifera* QToF_NEG_DIA (negative mode, data independent analysis – MS^e)

Guidelines and Examples for Completion of the Data Template .csv file

Instructions for naming file:

data_last name of person doing analysis_data type (orbi or qtof)_ion mode (pos or neg)_acquisition mode (FS, DDA, or DIA)_date (year XXXX, month XX, day XX; YYYYMMDD)

Please do not use spaces, dashes, extra underscores, or other punctuation in the filename.

Example:

data_manwill_orbi_pos_DDA_20211123 [Preston Manwill's analysis of positive ion mode DDA Orbitrap data November 23, 2021]
data_clark_qtof_neg_DIA_20220105 [Trevor Clark's analysis of negative ion mode DIA Q-ToF data analyzed on January 5, 2022]

The template consists of columns with labels as shown in the image below. Below are instructions for completing the columns for each **analyte**:

analyte_number	id	inchi	inchikey	family	exact_mass	mf	annotation	orthogonal	feature	rt	mz	area	compiled_features	notes

Instructions for Completion (Please do not modify the format of the table or columns)

Please use a separate row for each analyte. Please note that the term "feature" herein refers to an ion detected by the LC-MS with distinct *m/z* and retention time. The term "analyte" refers to the specific molecule (chemical entity) present in the sample. Even though multiple features may be detected for each analyte, each analyte should be represented in the table as just one row (**Figure S2 and S3**).

analyte_number = Analyte number, assigned in order of increasing retention time. An analyte is defined as a chemical entity, possibly represented in LC-MS by several features.

id = Proposed identity (common name(s)) of the analyte (if known).

inchi = IUPAC International Chemical Identifier (if known, can be found by searching https://chem.nlm.nih.gov/chemidplus/)

inchikey = A hashed version of the full InChI (if known, can be found by searching https://chem.nlm.nih.gov/chemidplus/)

Both InChI and InChIKey can be extracted from ChemDraw by selecting the molecule and choosing Edit -> Copy As -> InChI.

Alternatively, you can search PubChem or ChEMBL by compound name and copy the terms from there.

family = Chemical family to which the analyte belongs (if known, may be included even if analyte identity is not known). Please use the Superclass level of taxonomy from the NPClassifier ontology: https://npclassifier.ucsd.edu/# or https://lotus.naturalproducts.net/.

Examples include flavonoids, sesquiterpenoids, steroids, ornithine alkaloids, etc.

exact_mass = calculated monoisotopic mass for neutral analyte (can be calculated for instance with https://www.chemcalc.org/)

mf = Molecular formula of neutral analyte (matching monoisotopic mass) in order, C, H, N, O, P, S

annotation = level of annotation. Please include analytes in the table with level 2-5 annotation. See definitions below. Please assign to the analyte the annotation level for the feature with the highest confidence. For example, if both the $[M+H]^+$ feature with annotation level 2 and $[M+Na]^+$ feature with annotation level 3 are detected, the analyte is assigned an annotation level of 2 and the data for the $[M+H]^+$ feature are included in the table.

orthogonal = orthogonal information used to annotate. a-e possible, see definitions below, can use more than one letter, separated by a pipe character (|) (i.e., a|d)

rt = experimentally measured retention time (min) at the maximum for the chromatographic peak for the feature used in identification.

mz = experimentally measured mass to charge ratio for the relevant feature

feature = type of feature ($[M+H]^+$, $[M+Na]^+$, etc.) used to annotate the analyte. In the case where multiple features are detected for a given analyte, choose the feature that was most useful in determining structure.

area = experimentally measured peak area for the relevant feature. Average of three replicates (or indicate if only one replicate is used)

compiled_features = the number of different features (or redundant features) that have been observed for the analyte of interest (include $[M+H]^+$, $[M+Na]^+$, in-source fragments, etc. Do not include isotopes). For example, if a particular analyte is detected as $[M+H]^+$, $[M+Na]^+$, and $[M+H-H_2O]^+$, the value entered in this column would be 3. Please note that we do not ask you to provide the list of redundant features in this first round.

Annotation Level Definitions:

Level 0* = Unambiguous 3D Structure: Isolated Compound including full stereochemistry, following natural products guidelines, determination of 3D structure

Level 1* = Confirmed structure by reference standard. MS, MS/MS, RT, reference standard

Level 2 = Probable structure (literature, library): Unambiguous matching literature or library MS/MS spectrum

Level 3 = Probable structure (experimental, in silico): Structure determined based on MS, MS/MS fragments sub-structure matching with experimental data or in silico fragmentation.

Level 4 = Tentative candidate: Class of structures based on MS, MS/MS, experimental data, but positional isomers cannot be distinguished

Level 5 = Unequivocal molecular formula based on MS, isotopes, adducts, RDBE

Level 6: ** = Exact mass of interest, no proposed structure or formula. [Do not include in table]

*Note: Level 1 and Level 2 annotations are not possible with the data provided.

**Note: Do not include level 6 analytes in the table.

Orthogonal Information

Sub-level a: Previously described for the same Species
Sub-level b: Previously described for the same Genus
Sub-level c: Previously described for the same Family
Sub-level d*: match with UV spectrum in literature/library
Sub-level e: retention time prediction
Sub-level f*: ion mobility

*Note: sub-level f not possible with the data provided, sub-level d only available from the raw Orbitrap data.

analyte_nu	id	inchi	inchikey	family	exact_mas	mf	annotatior	orthogona	feature	rt	mz	area	compiled_
1	theobromi	InChI=1S/(	YAPQBXQYLJRXSA-UH		180.0647	C7H8N4O2	4	a	[M+H]+	1.52	181.073		1
2	gallocatec	InChI=1S/(	XMOCLSL(	flavanoid	306.074	C15H14O7	2	a	[M+H]+	1.55	307.081		4
3	epigallocat	InChI=1S/(	XMOCLSL(	flavanoid	306.074	C15H14O7	2	a	[M+H]+	1.94	307.081		7
4	catechin	InChI=1S/(	PFTAWBL(	flavanoid	290.079	C15H14O6	2	a	[M+H]+	2.1	291.085		3
5	Caffeine	InChI=1S/(	RYYVLZVU	alkaloid, xa	194.0804	C8H10N4O	2	a	[M+H]+	2.26	195.0886		6
6	nicotiflorir	InChI=1S/(	RTATXGUCZHCSNG-Q		594.1585	C27H30O1	3	a	[M+H]+	2.29	595.164		1
7	epicatechi	InChI=1S/(	PFTAWBL(	flavanoid	290.079	C15H14O6	2	a	[M+H]+	2.39	291.086		6
8	epigallocat	InChI=1S/(	WMBWRE	flavanoid	458.0849	C22H18O1	2	a	[M+H]+	2.4	459.091		6
9	gallocatec	nChI=1S/C	WMBWRE	flavanoid	458.0849	C22H18O1	2	a	[M+H]+	2.42	459.093		6
10	Epitheafla	InChI=1S/(	CMGRMM	flavanoid	552.0904	C27H20O1	4	a	[M+H]+	2.45	553.03		2
11	unknown analyte						5			2.51	554.034		
12	Vicenin 3	InChI=1S/(	MMDUKUSNQNWVET		564.1479	C26H28O1	4	a	[M+H]+	2.53	565.156		1
13	unknown analyte						5			2.62	319.045		
14	Epicatechi	InChI=1S/(	RHDJFGKN	flavanoid	594.101	C29H22O1	4	a	[M+H]+	2.68	595.166		1
15	unknown analyte						5			2.68	773.213		
16	unknown analyte						5			2.69	303.05		
17	unknown analyte						5			2.78	473.108		
18	unknown analyte						5			2.8	579.172		
19	rutin	InChI=1S/(	IKGXIBQEE	flavanoid g	610.1534	C27H30O1	2	a	[M+H]+	2.81	611.161		4
20	unknown analyte						5			2.87	433.206		
21	Kaempferc	InChI=1S/(	MFIXKYXSI	flavanoid	756.2113	C33H40O2	4	a	[M+H]+	2.89	757.218		2
22	epicatechi	InChI=1S/(	LSHVYAFM	flavanoid	442.09	C22H18O1	2	a	[M+H]+	2.91	443.098		9
23	unknown analyte						5			2.92	305.066		
24	unknown analyte						5			2.92	938.098		
25	unknown analyte						5			2.94	537.036		
26	unknown analyte						5			3.05	595.166		
27	unknown analyte						5			3.11	287.055		
28	unknown analyte						5			3.28	457.113		
29	TR-Saponi	InChI=1S/(	SPHKOEYMOPNIIA-CF		1028.519	C51H80O2	4	a	[M+Na]+	3.52	1051.29		1
30	unknown analyte						5			3.63	889.236		
31	unknown analyte						5			3.63	1051.29		
32	unknown analyte						5			3.67	1035.29		
33	unknown analyte						5			4.47	203.144		
34	Isovitexin	InChI=1S/(	MYXNWGACZJSMBT-		432.1056	C21H20O1	4	a	[M+H]+	4.93	433.207		2
35	unknown analyte						5			7.46	496.339		
36	unknown analyte						5			8.3	667.068		

Figure S2. Example of completed .csv file for DDA analysis of green tea (*Camellia sinensis*) extract with a Q-ToF in DDA positive ion mode from Laboratory A.

analyte_nu	id	inchi	inchikey	family	exact_mas	mf	annotatior	orthogona	feature	rt	mz	area	features de
1	sucrose|galacti	InChI=1S/(	CZMRCDW	disacchari	342.1162	C12H22O1	4		[M+H]+	0.45	343.1235	2.10E+04	2
2	L-theanine	InChI=1S/(	DATAGRP\	amino acic	174.1004	C7H14N2C	2	a	[M+H]+	0.51	175.1078	5.70E+04	3
3	unknown small peptide			amino acic	174.1004	C7H14N2C	5		[M+H]+	0.75	175.1075	6.60E+03	2
4	theobromine|tl	InChI=1S/(	YAPQBXQ\	alkaloid	180.0647	C7H8N4O2	4	a	[M+H]+	1.52	181.0718	1.80E+05	1
5	epigallocatechir	InChI=1S/(	XMOCLSL(	flavanol	306.074	C15H14O7	4	a	[M+H]+	1.55	307.0808	2.60E+04	2
6	dihydromyriceti	InChI=1S/(	KJXSIXMJH	flavanonol	320.0532	C15H12O8	3	a	[M+H]+	1.76	321.0602	1.80E+04	1
7	epigallocatechir	InChI=1S/(	XMOCLSL(	flavanol	306.074	C15H14O7	4	a	[M+H]+	1.92	307.081	1.50E+05	6
8	theasinensin C	InChI=1S/(	JPBGHWK\	dimeric fla	610.1323	C30H26O1	3	a	[M+H]+	1.92	611.1392	9.00E+03	1
9	catechin|epicat	InChI=1S/(	PFTAWBL(	flavanol	290.079	C15H14O6	4	a	[M+H]+	2.11	291.086	4.30E+04	3
10	Proanthocyanid	InChI=1S/(	XFZJEEAO\	proanthoc	578.1424	C30H26O1	3	a	[M+H]+	2.2	579.1499	5.00E+04	1
11	caffeine	InChI=1S/(	RYYVLZVU	alkaloid	194.0804	C8H10N4C	2	a	[M+H]+	2.2	195.088	5.40E+06	2
12	unknown analyte					C27H30O1	5		[M+H]+|is	2.31	595.1659	32157.23	1
13	kaempferol|fise	InChI=1S/(	IYRMWMY	flavonol|fl	286.0477	C15H10O6	4	a	[M+H]+	2.48	287.0548	3.10E+04	1
14	myricetin-3-O-	InChI=1S/(	FOHXFLPXI	flavonol gl	480.0904	C21H20O1	4	a	[M+H]+	2.6	481.0981	3.10E+04	1
15	quercetin-3-O-t	InChI=1S/(	XEFNBVWI	flavonol tr	772.2062	C33H40O2	4	a	[M+H]+	2.68	773.2141	2.00E+05	5
16	unknown analyte					C15H10O7	5		[M+H]+| is	2.68	303.0497	202863.3	1
17	4'-5-7-Trihydro›	InChI=1S/(	LYKXPTCU	flavone dig	578.1636	C27H30O1	3	a	[M+H]+	2.79	579.1716	1.10E+05	3
18	kaempferol rhai	InChI=1S/(	MFIXKYXSI	flavonol tr	756.2113	C33H40O2	3	a	[M+H]+	2.81	757.219	5.00E+04	3
19	unknown analyte					C15H10O7	5		[M+H]+| is	2.9	303.0497	149501.6	1
20	epicatechin-3-C	InChI=1S/(	LSHVYAFN	flavanol	442.09	C22H18O1	3	a	[M+H]+	2.92	443.0976	1.90E+05	6
21	kaempferol|fise	InChI=1S/(	IYRMWMY	flavonol|fl	286.0477	C15H10O1	4	a	[M+H]+	2.97	287.0544	6.50E+03	1
22	kaempferol|fise	InChI=1S/(	IYRMWMY	flavonol|fl	286.0477	C15H10O1	4	a	[M+H]+	3.19	287.0545	3.50E+04	1
23	epigallocatechir	InChI=1S/(	XGTBMCG	flavanol	456.1057	C23H20O1	3	a	[M+H]+	3.26	457.1137	1.70E+04	1
24	naringenin	InChI=1S/(	FTVWIRXF	flavanone	272.0685	C15H12O5	3	a	[M+H]+	3.27	273.0755	1.70E+04	1
25	Camelliquerceti	InChI=1S/(	YDPDTPHA	flavonol	1050.285	C47H54O2	4	a	[M+H]+	3.51	1051.294	4.30E+04	1
26	Camelliquerceti	InChI=1S/(	YDPDTPHA	flavonol	1050.285	C47H54O2	4	a	[M+H]+	3.62	1051.294	1.30E+04	3
27	unknown analyte					C14H18O	5		[M+H]+|is	4.48	203.1427	219378.3	1
28	unknown analyte					C20H32O1	5		[M+H]+| is	4.95	433.2069	62148.66	2
29	unknown analyte					C22H28O1	5		[M+Na]+|i	5.49	475.1577	39624.36	1
30	unknown analyte					C24H24O1	5		[M+H]+|is	5.7	473.1442	97590.65	1
31	unknown analyte					C24H24O6	5		[M+H]+|is	5.83	409.1619	53889.87	1
32	unknown analyte					C31H45NC	5		[M+H]+|is	7.47	496.3402	40123.24	1
33	unknown analyte					C38H62O1	5		[M+H]+|is	8.13	743.4192	14438.15	1
34	unknown analyte					C27H26O2	5		[M+H]+|is	8.13	383.2039	104965.4	1
35	arbutin	InChI=1S/(	BJRNKVDF	simple phe	272.0896	C12H16O7	3	a	[M+H]+	8.78	273.0964	3.10E+05	3
36	unknown analyte					C22H32O8	5		[M+H]+|is	8.87	425.2147	293084.9	1

Figure S3. Example of completed .csv file for DDA analysis of green tea (*Camellia sinensis*) extract with a Q-ToF in DDA positive ion mode from Laboratory B. (Same dataset as for Figure 2, different analyst).

Guidelines and Examples for Completion of the Data Processing and Annotation Narrative .docx File

We will invite you to upload a .docx file containing a description of your approach used to analyze the ***W. somnifera*** **(WS)** dataset(s), including methods and parameters for data processing (feature detection - peak picking, alignment, feature reduction - background subtraction, filtering, etc.) and any tools, databases, or other references used to assign structures (annotation steps). This will serve as a starting point for the methods section of the research publication, should you choose to participate in that portion.

Instructions for naming file:
last name of person doing analysis_data type (orbi or qtof)_ion mode (pos or neg)_acquisition mode (FS, DDA, or DIA)_date (year XXXX, month XX, day XX; YYYYMMDD)

Please do not use spaces, dashes, extra underscores, or other punctuation in the filename.

Naming Example:
narrative_manwill_orbi_pos_DDA_20211123 [Preston Manwill's analysis of positive ion mode DDA Orbitrap data November 23, 2021]
narrative_clark_qtof_neg_DIA_20220105 [Trevor Clark's analysis of negative ion mode DIA Q-ToF data analyzed on January 5, 2022]

Narrative Example:

The [instrument] [ion mode] [acquisition mode] RAW MS data were converted to .mzML using ProteoWizzard [1] and loaded to MZmine 2.53 [2]. The ADAP workflow [3] was employed with the following parameters: the mass detection step kept the ions that were above a noise level of XXX (and 0 for MS/MS). Chromatogram builder was employed with minimum group size in numbers of scan of 5, an *m/z* tolerance of XXX Da, and group intensity thresholds of XXX, and a minimum highest intensity of XXX. The chromatogram deconvolution was performed with a single noise threshold of XXX, the coefficient over area threshold was set at XXX, the RT wavelet scales from XXX to XXX min, the peak duration range from XXX to XXX min, and a minimum peak height of XXX. The MS/MS scan pairing parameters were set at XXX Da and XXX min. The feature lists were deisotoped using the isotope peak grouper with an *m/z* tolerance of XXX, a RT tolerance of XXX, a maximum charge of XXX, and the representative isotope used was the most intense. Peak alignment was obtained using the "join aligner" method with an *m/z* tolerance of XXX Da, an absolute RT tolerance of XXX min, a weight for *m/z* of XXX, a weight for RT of XXX, and an isotope pattern comparison of a minimum of XXX %. Gap-filling was applied with the "same RT and *m/z* range gap filler" module with an *m/z* tolerance set at XXX Da. Then, the "duplicate peak filter" module was applied with an *m/z* tolerance of XXX Da and an RT of XXX min to eliminate duplicates. When adapted, the "features list rows filter" module was employed to reduce the feature list to features with an MS/MS spectrum. The "identification" module was employed to annotate the peak list, including the "custom database search" which compiled the $[M+H]^+$ and some common adducts of compounds previously described for the same botanical species, genus, and family, and the "adduct search" which compiled adducts referenced in several sources[4-8]. This compilation contains common charge carriers (H^+, Na^+, K^+, Li^+), neutral gains and losses, and a combination of them, presented according to [4].

Next, the feature lists were exported and processed in Excel 16. Mean, standard deviation, and relative standard deviation (RSD) were first calculated for each set of replicates, as well as for all aligned samples (green tea and blank samples). Blank filtering consisted in 1) eliminating features not detected in green tea samples but only in blanks (mean in green tea samples equal to 0), 2) eliminating features detected in all samples (green tea and blank samples) with an RSD below XXX% (real features detected in all samples, i.e. solvent contaminants), and 3) eliminate feature with a blank ratio equal to or higher than XXX% (feature area ratio between green tea and blank, based on[9]. Then, the RSD filtering eliminated green tea features with an RSD above XXX%.

(1) Kessner, D.; Chambers, M.; Burke, R.; Agus, D.; Mallick, P. ProteoWizard: open source software for rapid proteomics tools development. *Bioinformatics* **2008**, *24* (21), 2534-2536. DOI: 10.1093/bioinformatics/btn323 From American Chemical Society . All Rights Reserved. CAPLUS.

(2) Pluskal, T.; Castillo, S.; Villar-Briones, A.; Oresic, M. MZmine 2: modular framework for processing, visualizing, and analyzing mass spectrometry-based molecular profile data. *BMC bioinformatics* **2010**, *11*, 395. DOI: doi.org/10.1186/1471-2105-11-395 From U.S. National Library of Medicine. MEDLINE.

(3) Myers, O. D.; Sumner, S. J.; Li, S.; Barnes, S.; Du, X. One Step Forward for Reducing False Positive and False Negative Compound Identifications from Mass Spectrometry Metabolomics Data: New Algorithms for Constructing Extracted Ion Chromatograms and Detecting Chromatographic Peaks. *Analytical Chemistry* **2017**, *89* (17), 8696-8703. DOI: 10.1021/acs.analchem.7b00947.

(4) Kachman, M.; Habra, H.; Duren, W.; Wigginton, J.; Sajjakulnukit, P.; Michailidis, G.; Burant, C.; Karnovsky, A. Deep annotation of untargeted LC-MS metabolomics data with Binner. *Bioinformatics* **2020**, *36* (6), 1801-1806. DOI: 10.1093/bioinformatics/btz798.

(5) Kuhl, C.; Tautenhahn, R.; Boettcher, C.; Larson, T. R.; Neumann, S. CAMERA: An Integrated Strategy for Compound Spectra Extraction and Annotation of Liquid Chromatography/Mass Spectrometry Data Sets. *Analytical Chemistry (Washington, DC, United States)* **2012**, *84* (1), 283-289. DOI: 10.1021/ac202450g From American Chemical Society . All Rights Reserved. CAPLUS.

(6) Fiehn, O. *Mass Spectrometry Adduct Calculator* Fiehn Lab, UC Davis, 2016. https://fiehnlab.ucdavis.edu/staff/kind/metabolomics/ms-adduct-calculator/ (accessed 20.08.2020).

(7) Draper, J.; Enot, D. P.; Parker, D.; Beckmann, M.; Snowdon, S.; Lin, W.; Zubair, H. Metabolite signal identification in accurate mass metabolomics data with MZedDB, an interactive m/z annotation tool utilising predicted ionisation behaviour 'rules'. *BMC bioinformatics* **2009**, *10*, 227. DOI: 10.1186/1471-2105-10-227.

(8) Damont, A.; Olivier, M. F.; Warnet, A.; Lyan, B.; Pujos-Guillot, E.; Jamin, E. L.; Debrauwer, L.; Bernillon, S.; Junot, C.; Tabet, J. C.; et al. Proposal for a chemically consistent way to annotate ions arising from the analysis of reference compounds under ESI conditions: A prerequisite to proper mass spectral database constitution in metabolomics. *J Mass Spectrom* **2019**, *54* (6), 567-582. DOI: 10.1002/jms.4372. Li, H.-J.; Deinzer, M. L. Tandem Mass Spectrometry for Sequencing Proanthocyanidins. *Analytical Chemistry* **2007**, *79* (4), 1739-1748. DOI: 10.1021/ac061823v.

(9) Fraisier-Vannier, O.; Chervin, J.; Cabanac, G.; Puech, V.; Fournier, S.; Durand, V.; Amiel, A.; André, O.; Benamar, O. A.; Dumas, B.; et al. MS-CleanR: A Feature-Filtering Workflow for Untargeted LC–MS Based Metabolomics. *Analytical Chemistry* **2020**, *92* (14), 9971-9981. DOI: 10.1021/acs.analchem.0c01594.

DESCRIPTION OF THE DATASET

We are providing access to a set of data obtained by analyzing three replicate methanolic extracts of the plant ashwagandha (*Withania somnifera*). We are not asking you to collect any data, just to analyze this existing data. The dataset includes data acquired with three acquisition modes, full scan (FS), data-dependent MS-MS acquisition (DDA), and data-independent fragment acquisition (DIA; MS^e/AIF) on two different platforms, a Waters Synapt G2-Si and a Thermo Q-Exactive Plus Orbitrap mass spectrometer. We have collected both positive and negative ion mode datasets, which equates to a total of twelve different data types for each extract replicate, each acquired with a different acquisition mode or instrument platform. All of the data were acquired with the same LC-MS conditions and column on a Waters Acquity UPLC. The dataset also includes triplicate solvent blanks and triplicate extraction (process) blanks for every acquisition mode and platform. Participants will have the opportunity to choose which acquisition mode (full scan, DDA, DIA - MS^e/AIF) and platform (Waters Synapt G2-Si or Thermo Q-Exactive Plus Orbitrap) they prefer to analyze. Please choose at least one positive ion mode, and then analyze and submit the results using the survey (with .docx and .csv files attached) as described under "Instructions for Data Analysis". If you wish to analyze a second data type (i.e., the corresponding negative ion mode data), please complete a separate survey with separate attachments and submit the results separately.

Chromatographic conditions used:

Chromatographic separation was performed using an Acquity UPLC I-Class with an Acquity HSS T3 C18 column 1.8 µm (2.1 x 100 mm). Mobile phase A was 100% CH_3CN and mobile phase B was 100% H_2O, with both containing 0.01% HCOOH. Gradient elution mode was as follows: 0 - 0.3 min, 5% A; 0.3 – 9.1 min, 5% to 90% A; 9.1 – 10.7 min, 90 to 98% A; 10.7 – 11.0 min, 98% A; 11.01 – 12.8 min, 5% A. 0.5 mL/min, column temperature 40°C, injection volume 5 µL.

MS Data acquisition:

Q-ToF: Data were acquired on a Synapt G2-Si hybrid quadrupole-traveling wave ion mobility (TWIM)-time-of-flight (TOF) mass spectrometer equipped with an electrospray ionization (ESI) source. Acquisitions were made in either ESI+ for positive ionization mode or ESI – for negative ionization mode; voltage 3.0 kV; cone voltage 35 V; source offset 50 V; source temperature 150°C; desolvation temperature 300°C; cone gas flow 30 L/h; desolvation gas flow 600 L/h. Mass measurements were recorded using either Full Scan (FS), FS – Data Dependent Acquisition (DDA), or FS – Data Independent Acquisition (DIA) experiments. Detection was performed in the *m/z* range 50-1500 with a scan rate of 0.1 Hz in both MS1 and MS2 experiments. Leucine enkephalin was employed as the lockspray solution at a concentration of 200 pg/µL at 0.10 Hz with scans to average set to 3. In DDA mode precursor selection was set to 3 with an intensity threshold of 5.0×10^3 for selection and a real-time exclusion set to 3.0 seconds. Collision energy for DDA and DIA was set to 30 eV.

Orbi: Data were acquired on a Q Exactive Plus Hybrid Quadrupole-Orbitrap mass spectrometer (Thermo Fisher Scientific) equipped with a heated electrospray ionization (HESI) source. The ion source was operated in either positive or negative ionization mode using the following parameters: spray voltage of 3.5 kV, heater temperature of 450 °C, capillary temperature of 275 °C, S-Lens RF level of 50, sheath gas, auxiliary gas, and spare gas of 55, 15, and 3 (arbitrary units), respectively. Nitrogen was used as the source gas and as the collision gas. Mass measurements were recorded using either Full Scan (FS), FS – Data Dependent Acquisition (DDA), or FS – Data Independent Acquisition (DIA) experiments. Detection was performed in the *m/z* range 100-1500 with a resolving power of 35,000, an AGC target of 1.0×10^6, and a maximum injection time (IT) of 100 milliseconds. The DDA occurred with a resolving power of 17,500, an AGC target of 1.0×10^5, a maximum IT of 50 milliseconds, an isolation window of 1.0 Da, a collision energy of 30 eV, and an intensity threshold of 1.6×10^5. The DIA occurred with a resolving power of 35,000, an AGC target of 1.0×10^6, a maximum IT of 100 milliseconds, and a collision energy of 30 eV.

Data conversion:

All .raw files were converted to .mzML using ProteoWizard MSConvert using default settings with the following exceptions: binary encoding precision set to 32-bit, did not use zlib compression, the filter Peak Picking for vendor msLevel 1-2 added as first step of processing.

LINKS TO MASS SPECTROMETRY EVALUATION DATA

All datasets can be accessible via links in this document.

The data were acquired using two mass spectrometry platforms, a Waters Synapt G2-Si (QToF) and a Thermo Q-Exactive Plus Orbitrap (Orbi).

The data were acquired using three acquisition modes, full scan (FS/E1), data-dependent MS-MS acquisition (DDA/E2), and data-independent fragment acquisition (DIA/E3; MS^e or AIF).

We have collected both positive (Pos/P) and negative (Neg/N) ion mode datasets, which equates to a total of 12 different data types. A full list of all data files is at the end of this document.

We have created two MassIVE links, one for the Orbitrap datasets and another for the Q-ToF datasets. Each MassIVE link contains 12 folders for each polarity, acquisition, and file type. These folders are labeled with the instrument (Orbi or QToF), the polarity [Positive (POS/P) or Negative (NEG/N)], the acquisition type [Full scan (FS/E1), data dependent (DDA/E2), or data independent (DIA/E3)], and the file type (Raw or mzML). Each folder contains 9 files: the extraction blanks (EXBLANK), methanol:H_2O/solvent blanks (Wasteblank) and the botanical sample of interest, *Withania somnifera* (WS03) for the evaluation dataset, each in triplicate (ABC or 456).

The links will take you to the MassIVE website and respective datasets. We recommend you copy the URL from the webpage and paste it into your preferred file transfer protocol (FTP) client program (**Fig. S4** or MassIVE documentation).

Orbitrap *W. somnifera* Evaluation dataset: MassIVE MSV000089047 [doi:10.25345/C5ZK55Q3W]

Password: orbi

Q-ToF *W. somnifera* Evaluation dataset: MassIVE MSV000089033 [doi:10.25345/C5S17SW57]

Password: qtof

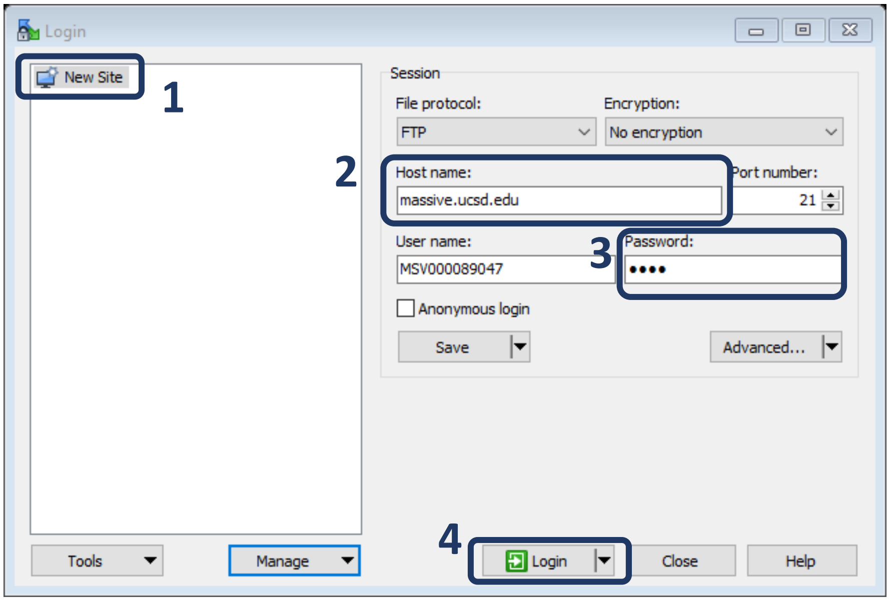

Figure S4. An example of how to set up your FTP client to access the MassIVE datasets. 1. Select New Site, 2. Paste the MassIVE URL, 3. Enter the password, and 4. Login

Full List of Evaluation Data Files for the MS Metabolomics Annotation Collaboration

WS QToF Evaluation Dataset

**naming structure**	_X	_XX	_samplename	.raw or .mzml
instrument	N = Negative mode	E1 = Full scan	Wasteblank = Methanol:H2O blank	
	P = Positive mode	E2 = DDA	EXBLANK = extraction blank	
		E3 = DIA	Alphanumeric code for plant sample	
			CS = Example dataset	
			WS = Evaluation dataset	

Full list of data files.

Qtof_P_E1_Wasteblank4.raw	positive	Full scan	MeOH:H2O blank	.raw
Qtof_P_E1_Wasteblank5.raw	positive	Full scan	MeOH:H2O blank	.raw
Qtof_P_E1_Wasteblank6.raw	positive	Full scan	MeOH:H2O blank	.raw
Qtof_P_E1_EXBLANKA.raw	positive	Full scan	Extraction blank	.raw
Qtof_P_E1_EXBLANKB.raw	positive	Full scan	Extraction blank	.raw
Qtof_P_E1_EXBLANKC.raw	positive	Full scan	Extraction blank	.raw
Qtof_P_E1_WS03A.raw	positive	Full scan	Evaluation sample	.raw
Qtof_P_E1_WS03B.raw	positive	Full scan	Evaluation sample	.raw
Qtof_P_E1_WS03C.raw	positive	Full scan	Evaluation sample	.raw
Qtof_P_E1_Wasteblank4.mzml	positive	Full scan	MeOH:H2O blank	.mzml
Qtof_P_E1_Wasteblank5.mzml	positive	Full scan	MeOH:H2O blank	.mzml
Qtof_P_E1_Wasteblank6.mzml	positive	Full scan	MeOH:H2O blank	.mzml
Qtof_P_E1_EXBLANKA.mzml	positive	Full scan	Extraction blank	.mzml
Qtof_P_E1_EXBLANKB.mzml	positive	Full scan	Extraction blank	.mzml
Qtof_P_E1_EXBLANKC.mzml	positive	Full scan	Extraction blank	.mzml
Qtof_P_E1_WS03A.mzml	positive	Full scan	Evaluation sample	.mzml
Qtof_P_E1_WS03B.mzml	positive	Full scan	Evaluation sample	.mzml
Qtof_P_E1_WS03C.mzml	positive	Full scan	Evaluation sample	.mzml
Qtof_N_E1_Wasteblank4.raw	negative	Full scan	MeOH:H2O blank	.raw
Qtof_N_E1_Wasteblank5.raw	negative	Full scan	MeOH:H2O blank	.raw
Qtof_N_E1_Wasteblank6.raw	negative	Full scan	MeOH:H2O blank	.raw
Qtof_N_E1_EXBLANKA.raw	negative	Full scan	Extraction blank	.raw
Qtof_N_E1_EXBLANKB.raw	negative	Full scan	Extraction blank	.raw
Qtof_N_E1_EXBLANKC.raw	negative	Full scan	Extraction blank	.raw
Qtof_N_E1_WS03A.raw	negative	Full scan	Evaluation sample	.raw
Qtof_N_E1_WS03B.raw	negative	Full scan	Evaluation sample	.raw
Qtof_N_E1_WS03C.raw	negative	Full scan	Evaluation sample	.raw

Qtof_N_E1_Wasteblank4.mzml	negative	Full scan	MeOH:H2O blank	.mzml
Qtof_N_E1_Wasteblank5.mzml	negative	Full scan	MeOH:H2O blank	.mzml
Qtof_N_E1_Wasteblank6.mzml	negative	Full scan	MeOH:H2O blank	.mzml
Qtof_N_E1_EXBLANKA.mzml	negative	Full scan	Extraction blank	.mzml
Qtof_N_E1_EXBLANKB.mzml	negative	Full scan	Extraction blank	.mzml
Qtof_N_E1_EXBLANKC.mzml	negative	Full scan	Extraction blank	.mzml
Qtof_N_E1_WS03A.mzml	negative	Full scan	Evaluation sample	.mzml
Qtof_N_E1_WS03B.mzml	negative	Full scan	Evaluation sample	.mzml
Qtof_N_E1_WS03C.mzml	negative	Full scan	Evaluation sample	.mzml
Qtof_P_E2_Wasteblank4.raw	positive	DDA	MeOH:H2O blank	.raw
Qtof_P_E2_Wasteblank5.raw	positive	DDA	MeOH:H2O blank	.raw
Qtof_P_E2_Wasteblank6.raw	positive	DDA	MeOH:H2O blank	.raw
Qtof_P_E2_EXBLANKA.raw	positive	DDA	Extraction blank	.raw
Qtof_P_E2_EXBLANKB.raw	positive	DDA	Extraction blank	.raw
Qtof_P_E2_EXBLANKC.raw	positive	DDA	Extraction blank	.raw
Qtof_P_E2_WS03A.raw	positive	DDA	Evaluation sample	.raw
Qtof_P_E2_WS03B.raw	positive	DDA	Evaluation sample	.raw
Qtof_P_E2_WS03C.raw	positive	DDA	Evaluation sample	.raw
Qtof_P_E2_Wasteblank4.mzml	positive	DDA	MeOH:H2O blank	.mzml
Qtof_P_E2_Wasteblank5.mzml	positive	DDA	MeOH:H2O blank	.mzml
Qtof_P_E2_Wasteblank6.mzml	positive	DDA	MeOH:H2O blank	.mzml
Qtof_P_E2_EXBLANKA.mzml	positive	DDA	Extraction blank	.mzml
Qtof_P_E2_EXBLANKB.mzml	positive	DDA	Extraction blank	.mzml
Qtof_P_E2_EXBLANKC.mzml	positive	DDA	Extraction blank	.mzml
Qtof_P_E2_WS03A.mzml	positive	DDA	Evaluation sample	.mzml
Qtof_P_E2_WS03B.mzml	positive	DDA	Evaluation sample	.mzml
Qtof_P_E2_WS03C.mzml	positive	DDA	Evaluation sample	.mzml
Qtof_N_E2_Wasteblank4.raw	negative	DDA	MeOH:H2O blank	.raw
Qtof_N_E2_Wasteblank5.raw	negative	DDA	MeOH:H2O blank	.raw
Qtof_N_E2_Wasteblank6.raw	negative	DDA	MeOH:H2O blank	.raw
Qtof_N_E2_EXBLANKA.raw	negative	DDA	Extraction blank	.raw
Qtof_N_E2_EXBLANKB.raw	negative	DDA	Extraction blank	.raw
Qtof_N_E2_EXBLANKC.raw	negative	DDA	Extraction blank	.raw
Qtof_N_E2_WS03A.raw	negative	DDA	Evaluation sample	.raw
Qtof_N_E2_WS03B.raw	negative	DDA	Evaluation sample	.raw
Qtof_N_E2_WS03C.raw	negative	DDA	Evaluation sample	.raw
Qtof_N_E2_Wasteblank4.mzml	negative	DDA	MeOH:H2O blank	.mzml
Qtof_N_E2_Wasteblank5.mzml	negative	DDA	MeOH:H2O blank	.mzml
Qtof_N_E2_Wasteblank6.mzml	negative	DDA	MeOH:H2O blank	.mzml
Qtof_N_E2_EXBLANKA.mzml	negative	DDA	Extraction blank	.mzml
Qtof_N_E2_EXBLANKB.mzml	negative	DDA	Extraction blank	.mzml
Qtof_N_E2_EXBLANKC.mzml	negative	DDA	Extraction blank	.mzml
Qtof_N_E2_WS03A.mzml	negative	DDA	Evaluation sample	.mzml
Qtof_N_E2_WS03B.mzml	negative	DDA	Evaluation sample	.mzml

Qtof_N_E2_WS03C.mzml	negative	DDA	Evaluation sample	.mzml
Qtof_P_E3_Wasteblank4.raw	positive	DIA	MeOH:H2O blank	.raw
Qtof_P_E3_Wasteblank5.raw	positive	DIA	MeOH:H2O blank	.raw
Qtof_P_E3_Wasteblank6.raw	positive	DIA	MeOH:H2O blank	.raw
Qtof_P_E3_EXBLANKA.raw	positive	DIA	Extraction blank	.raw
Qtof_P_E3_EXBLANKB.raw	positive	DIA	Extraction blank	.raw
Qtof_P_E3_EXBLANKC.raw	positive	DIA	Extraction blank	.raw
Qtof_P_E3_WS03A.raw	positive	DIA	Evaluation sample	.raw
Qtof_P_E3_WS03B.raw	positive	DIA	Evaluation sample	.raw
Qtof_P_E3_WS03C.raw	positive	DIA	Evaluation sample	.raw
Qtof_P_E3_Wasteblank4.mzml	positive	DIA	MeOH:H2O blank	.mzml
Qtof_P_E3_Wasteblank5.mzml	positive	DIA	MeOH:H2O blank	.mzml
Qtof_P_E3_Wasteblank6.mzml	positive	DIA	MeOH:H2O blank	.mzml
Qtof_P_E3_EXBLANKA.mzml	positive	DIA	Extraction blank	.mzml
Qtof_P_E3_EXBLANKB.mzml	positive	DIA	Extraction blank	.mzml
Qtof_P_E3_EXBLANKC.mzml	positive	DIA	Extraction blank	.mzml
Qtof_P_E3_WS03A.mzml	positive	DIA	Evaluation sample	.mzml
Qtof_P_E3_WS03B.mzml	positive	DIA	Evaluation sample	.mzml
Qtof_P_E3_WS03C.mzml	positive	DIA	Evaluation sample	.mzml
Qtof_N_E3_Wasteblank4.raw	negative	DIA	MeOH:H2O blank	.raw
Qtof_N_E3_Wasteblank5.raw	negative	DIA	MeOH:H2O blank	.raw
Qtof_N_E3_Wasteblank6.raw	negative	DIA	MeOH:H2O blank	.raw
Qtof_N_E3_EXBLANKA.raw	negative	DIA	Extraction blank	.raw
Qtof_N_E3_EXBLANKB.raw	negative	DIA	Extraction blank	.raw
Qtof_N_E3_EXBLANKC.raw	negative	DIA	Extraction blank	.raw
Qtof_N_E3_WS03A.raw	negative	DIA	Evaluation sample	.raw
Qtof_N_E3_WS03B.raw	negative	DIA	Evaluation sample	.raw
Qtof_N_E3_WS03C.raw	negative	DIA	Evaluation sample	.raw
Qtof_N_E3_Wasteblank4.mzml	negative	DIA	MeOH:H2O blank	.mzml
Qtof_N_E3_Wasteblank5.mzml	negative	DIA	MeOH:H2O blank	.mzml
Qtof_N_E3_Wasteblank6.mzml	negative	DIA	MeOH:H2O blank	.mzml
Qtof_N_E3_EXBLANKA.mzml	negative	DIA	Extraction blank	.mzml
Qtof_N_E3_EXBLANKB.mzml	negative	DIA	Extraction blank	.mzml
Qtof_N_E3_EXBLANKC.mzml	negative	DIA	Extraction blank	.mzml
Qtof_N_E3_WS03A.mzml	negative	DIA	Evaluation sample	.mzml
Qtof_N_E3_WS03B.mzml	negative	DIA	Evaluation sample	.mzml
Qtof_N_E3_WS03C.mzml	negative	DIA	Evaluation sample	.mzml

**naming structure**	_X	_XX	_samplename	.raw or .mzml
instrument	N = Negative mode	E1 = Full scan	Wasteblank = Methanol:H2O blank	
	P = Positive mode	E2 = DDA	EXBLANK = extraction blank	
		E3 = DIA	Alphanumeric code for plant sample	
			CS = Example dataset	
			WS = Evaluation dataset	

Full list of data files.

Orbi_P_E1_Wasteblank4.raw	positive	Full scan	MeOH:H2O blank	.raw
Orbi_P_E1_Wasteblank5.raw	positive	Full scan	MeOH:H2O blank	.raw
Orbi_P_E1_Wasteblank6.raw	positive	Full scan	MeOH:H2O blank	.raw
Orbi_P_E1_EXBLANKA.raw	positive	Full scan	Extraction blank	.raw
Orbi_P_E1_EXBLANKB.raw	positive	Full scan	Extraction blank	.raw
Orbi_P_E1_EXBLANKC.raw	positive	Full scan	Extraction blank	.raw
Orbi_P_E1_WS03A.raw	positive	Full scan	Evaluation sample	.raw
Orbi_P_E1_WS03B.raw	positive	Full scan	Evaluation sample	.raw
Orbi_P_E1_WS03C.raw	positive	Full scan	Evaluation sample	.raw
Orbi_P_E1_Wasteblank4.mzml	positive	Full scan	MeOH:H2O blank	.mzml
Orbi_P_E1_Wasteblank5.mzml	positive	Full scan	MeOH:H2O blank	.mzml
Orbi_P_E1_Wasteblank6.mzml	positive	Full scan	MeOH:H2O blank	.mzml
Orbi_P_E1_EXBLANKA.mzml	positive	Full scan	Extraction blank	.mzml
Orbi_P_E1_EXBLANKB.mzml	positive	Full scan	Extraction blank	.mzml
Orbi_P_E1_EXBLANKC.mzml	positive	Full scan	Extraction blank	.mzml
Orbi_P_E1_WS03A.mzml	positive	Full scan	Evaluation sample	.mzml
Orbi_P_E1_WS03B.mzml	positive	Full scan	Evaluation sample	.mzml
Orbi_P_E1_WS03C.mzml	positive	Full scan	Evaluation sample	.mzml
Orbi_N_E1_Wasteblank4.raw	negative	Full scan	MeOH:H2O blank	.raw
Orbi_N_E1_Wasteblank5.raw	negative	Full scan	MeOH:H2O blank	.raw
Orbi_N_E1_Wasteblank6.raw	negative	Full scan	MeOH:H2O blank	.raw
Orbi_N_E1_EXBLANKA.raw	negative	Full scan	Extraction blank	.raw
Orbi_N_E1_EXBLANKB.raw	negative	Full scan	Extraction blank	.raw
Orbi_N_E1_EXBLANKC.raw	negative	Full scan	Extraction blank	.raw
Orbi_N_E1_WS03A.raw	negative	Full scan	Evaluation sample	.raw
Orbi_N_E1_WS03B.raw	negative	Full scan	Evaluation sample	.raw
Orbi_N_E1_WS03C.raw	negative	Full scan	Evaluation sample	.raw
Orbi_N_E1_Wasteblank4.mzml	negative	Full scan	MeOH:H2O blank	.mzml
Orbi_N_E1_Wasteblank5.mzml	negative	Full scan	MeOH:H2O blank	.mzml
Orbi_N_E1_Wasteblank6.mzml	negative	Full scan	MeOH:H2O blank	.mzml

Orbi_N_E1_EXBLANKA.mzml	negative	Full scan	Extraction blank	.mzml
Orbi_N_E1_EXBLANKB.mzml	negative	Full scan	Extraction blank	.mzml
Orbi_N_E1_EXBLANKC.mzml	negative	Full scan	Extraction blank	.mzml
Orbi_N_E1_WS03A.mzml	negative	Full scan	Evaluation sample	.mzml
Orbi_N_E1_WS03B.mzml	negative	Full scan	Evaluation sample	.mzml
Orbi_N_E1_WS03C.mzml	negative	Full scan	Evaluation sample	.mzml
Orbi_P_E2_Wasteblank4.raw	positive	DDA	MeOH:H2O blank	.raw
Orbi_P_E2_Wasteblank5.raw	positive	DDA	MeOH:H2O blank	.raw
Orbi_P_E2_Wasteblank6.raw	positive	DDA	MeOH:H2O blank	.raw
Orbi_P_E2_EXBLANKA.raw	positive	DDA	Extraction blank	.raw
Orbi_P_E2_EXBLANKB.raw	positive	DDA	Extraction blank	.raw
Orbi_P_E2_EXBLANKC.raw	positive	DDA	Extraction blank	.raw
Orbi_P_E2_WS03A.raw	positive	DDA	Evaluation sample	.raw
Orbi_P_E2_WS03B.raw	positive	DDA	Evaluation sample	.raw
Orbi_P_E2_WS03C.raw	positive	DDA	Evaluation sample	.raw
Orbi_P_E2_Wasteblank4.mzml	positive	DDA	MeOH:H2O blank	.mzml
Orbi_P_E2_Wasteblank5.mzml	positive	DDA	MeOH:H2O blank	.mzml
Orbi_P_E2_Wasteblank6.mzml	positive	DDA	MeOH:H2O blank	.mzml
Orbi_P_E2_EXBLANKA.mzml	positive	DDA	Extraction blank	.mzml
Orbi_P_E2_EXBLANKB.mzml	positive	DDA	Extraction blank	.mzml
Orbi_P_E2_EXBLANKC.mzml	positive	DDA	Extraction blank	.mzml
Orbi_P_E2_WS03A.mzml	positive	DDA	Evaluation sample	.mzml
Orbi_P_E2_WS03B.mzml	positive	DDA	Evaluation sample	.mzml
Orbi_P_E2_WS03C.mzml	positive	DDA	Evaluation sample	.mzml
Orbi_N_E2_Wasteblank4.raw	negative	DDA	MeOH:H2O blank	.raw
Orbi_N_E2_Wasteblank5.raw	negative	DDA	MeOH:H2O blank	.raw
Orbi_N_E2_Wasteblank6.raw	negative	DDA	MeOH:H2O blank	.raw
Orbi_N_E2_EXBLANKA.raw	negative	DDA	Extraction blank	.raw
Orbi_N_E2_EXBLANKB.raw	negative	DDA	Extraction blank	.raw
Orbi_N_E2_EXBLANKC.raw	negative	DDA	Extraction blank	.raw
Orbi_N_E2_WS03A.raw	negative	DDA	Evaluation sample	.raw
Orbi_N_E2_WS03B.raw	negative	DDA	Evaluation sample	.raw
Orbi_N_E2_WS03C.raw	negative	DDA	Evaluation sample	.raw
Orbi_N_E2_Wasteblank4.mzml	negative	DDA	MeOH:H2O blank	.mzml
Orbi_N_E2_Wasteblank5.mzml	negative	DDA	MeOH:H2O blank	.mzml
Orbi_N_E2_Wasteblank6.mzml	negative	DDA	MeOH:H2O blank	.mzml
Orbi_N_E2_EXBLANKA.mzml	negative	DDA	Extraction blank	.mzml
Orbi_N_E2_EXBLANKB.mzml	negative	DDA	Extraction blank	.mzml
Orbi_N_E2_EXBLANKC.mzml	negative	DDA	Extraction blank	.mzml
Orbi_N_E2_WS03A.mzml	negative	DDA	Evaluation sample	.mzml
Orbi_N_E2_WS03B.mzml	negative	DDA	Evaluation sample	.mzml
Orbi_N_E2_WS03C.mzml	negative	DDA	Evaluation sample	.mzml
Orbi_P_E3_Wasteblank4.raw	positive	DIA	MeOH:H2O blank	.raw

Orbi_P_E3_Wasteblank5.raw	positive	DIA	MeOH:H2O blank	.raw
Orbi_P_E3_Wasteblank6.raw	positive	DIA	MeOH:H2O blank	.raw
Orbi_P_E3_EXBLANKA.raw	positive	DIA	Extraction blank	.raw
Orbi_P_E3_EXBLANKB.raw	positive	DIA	Extraction blank	.raw
Orbi_P_E3_EXBLANKC.raw	positive	DIA	Extraction blank	.raw
Orbi_P_E3_WS03A.raw	positive	DIA	Evaluation sample	.raw
Orbi_P_E3_WS03B.raw	positive	DIA	Evaluation sample	.raw
Orbi_P_E3_WS03C.raw	positive	DIA	Evaluation sample	.raw
Orbi_P_E3_Wasteblank4.mzml	positive	DIA	MeOH:H2O blank	.mzml
Orbi_P_E3_Wasteblank5.mzml	positive	DIA	MeOH:H2O blank	.mzml
Orbi_P_E3_Wasteblank6.mzml	positive	DIA	MeOH:H2O blank	.mzml
Orbi_P_E3_EXBLANKA.mzml	positive	DIA	Extraction blank	.mzml
Orbi_P_E3_EXBLANKB.mzml	positive	DIA	Extraction blank	.mzml
Orbi_P_E3_EXBLANKC.mzml	positive	DIA	Extraction blank	.mzml
Orbi_P_E3_WS03A.mzml	positive	DIA	Evaluation sample	.mzml
Orbi_P_E3_WS03B.mzml	positive	DIA	Evaluation sample	.mzml
Orbi_P_E3_WS03C.mzml	positive	DIA	Evaluation sample	.mzml
Orbi_N_E3_Wasteblank4.raw	negative	DIA	MeOH:H2O blank	.raw
Orbi_N_E3_Wasteblank5.raw	negative	DIA	MeOH:H2O blank	.raw
Orbi_N_E3_Wasteblank6.raw	negative	DIA	MeOH:H2O blank	.raw
Orbi_N_E3_EXBLANKA.raw	negative	DIA	Extraction blank	.raw
Orbi_N_E3_EXBLANKB.raw	negative	DIA	Extraction blank	.raw
Orbi_N_E3_EXBLANKC.raw	negative	DIA	Extraction blank	.raw
Orbi_N_E3_WS03A.raw	negative	DIA	Evaluation sample	.raw
Orbi_N_E3_WS03B.raw	negative	DIA	Evaluation sample	.raw
Orbi_N_E3_WS03C.raw	negative	DIA	Evaluation sample	.raw
Orbi_N_E3_Wasteblank4.mzml	negative	DIA	MeOH:H2O blank	.mzml
Orbi_N_E3_Wasteblank5.mzml	negative	DIA	MeOH:H2O blank	.mzml
Orbi_N_E3_Wasteblank6.mzml	negative	DIA	MeOH:H2O blank	.mzml
Orbi_N_E3_EXBLANKA.mzml	negative	DIA	Extraction blank	.mzml
Orbi_N_E3_EXBLANKB.mzml	negative	DIA	Extraction blank	.mzml
Orbi_N_E3_EXBLANKC.mzml	negative	DIA	Extraction blank	.mzml
Orbi_N_E3_WS03A.mzml	negative	DIA	Evaluation sample	.mzml
Orbi_N_E3_WS03B.mzml	negative	DIA	Evaluation sample	.mzml
Orbi_N_E3_WS03C.mzml	negative	DIA	Evaluation sample	.mzml

EXAMPLE DATASET FOR YOUR INTERNAL BENCHMARKING

Here we provide two MassIVE links for *Camelia sinensis* (CS02), an Example Dataset. The CS dataset includes 12 files per data type: the extraction blanks (EXBLANK), methanol:H2O/solvent blanks (Wasteblank), the botanical sample of interest, *Camelia sinensis* (CS02), and a mixture of standard compounds at a concentration of 10 µM (CSSM10uM) (**Table S1**), each in triplicate (ABC or 456). The CS-Example dataset may be used for practice/benchmarking but will not be included in the Metabolomics Annotation Collaboration and follow-up survey.

Orbitrap *C. sinensis* Example dataset: MassIVE MSV000089046 [doi:10.25345/C53B5WB8W]

Password: orbi

Q-ToF *C. sinensis* Example dataset: MassIVE MSV000089034 [doi:10.25345/C5N87331W]

Password: qtof

Table S1. *Camellia sinensis* (CS) reference compounds included in CS standard mixture (CSSM). All compounds were prepared to the final concentration of 10 µM in a solution of Methanol:H_2O (1:1).

CAS #	Name	Mol. Weight	Molecular formula	Monoisotopic mass	RT in Lab A	RT in Lab B
331-39-5	caffeic acid	180.157	$C_9H_8O_4$	180.0423	2.34	2.52
327-97-9	chlorogenic acid	354.309	$C_{16}H_{18}O_9$	354.0951	2.01	2.19
501-98-4	coumaric acid	164.158	$C_9H_8O_3$	164.0473	2.82	3.01
490-46-0	epicatechin, (–)-	290.268	$C_{15}H_{14}O_6$	290.0790	2.40	2.58
1257-08-5	epicatechin gallate, (–)-	442.372	$C_{22}H_{18}O_{10}$	442.0900	2.92	3.11
970-74-1	epigallocatechin, (–)-	306.267	$C_{15}H_{14}O_7$	306.0740	1.93	2.10
149-91-7	gallic acid	170.12	$C_7H_6O_5$	170.0215	1.11	1.26
970-73-0	gallocatechin, (–)-	306.267	$C_{15}H_{14}O_7$	306.0740	1.56	1.74
4233-96-9	gallocatechin gallate, (–)-	458.372	$C_{22}H_{18}O_{11}$	458.0849	2.52	2.70
520-18-3	kaempferol	286.236	$C_{15}H_{10}O_6$	286.0477	4.59	4.80
529-44-2	myricetin	318.235	$C_{15}H_{10}O_8$	318.0376	3.47	3.66
153-18-4	rutin	610.518	$C_{27}H_{30}O_{16}$	610.1534	2.81	2.97
3081-61-6	theanine, *L*-	174.198	$C_7H_{14}N_2O_3$	174.1004	0.53	0.59
58-08-2	caffeine	194.191	$C_8H_{10}N_4O_2$	194.0804	2.20	2.36
117-39-5	quercetin	302.236	$C_{15}H_{10}O_7$	302.0427	4.05	4.25
154-23-4	catechin, (+)-	290.268	$C_{15}H_{14}O_6$	290.0790	2.11	2.29
989-51-5	epigallocatechin gallate	458.372	$C_{22}H_{18}O_{11}$	458.0849	2.42	2.60

*CS QToF Example Dataset**

For your own benchmarking only – do not submit analyses of these data to the group)

**naming structure**	_X	_XX	_samplename	.raw or .mzml
instrument	N = Negative mode	E1 = Full scan	Wasteblank = Methanol:H2O blank	
	P = Positive mode	E2 = DDA	EXBLANK = extraction blank	
		E3 = DIA	Alphanumeric code for plant sample	
			CS = Example dataset	
			WS = Evaluation dataset	
			CSSM10uM = mixture of 17 standards	

Full list of data files.

Qtof_P_E1_Wasteblank4.raw	positive	Full scan	MeOH:H2O blank	.raw
Qtof_P_E1_Wasteblank5.raw	positive	Full scan	MeOH:H2O blank	.raw
Qtof_P_E1_Wasteblank6.raw	positive	Full scan	MeOH:H2O blank	.raw
Qtof_P_E1_EXBLANKA.raw	positive	Full scan	Extraction blank	.raw
Qtof_P_E1_EXBLANKB.raw	positive	Full scan	Extraction blank	.raw
Qtof_P_E1_EXBLANKC.raw	positive	Full scan	Extraction blank	.raw
Qtof_P_E1_CS02A.raw	positive	Full scan	Example sample	.raw
Qtof_P_E1_CS02B.raw	positive	Full scan	Example sample	.raw
Qtof_P_E1_CS02C.raw	positive	Full scan	Example sample	.raw
Qtof_P_E1_CSSM10uMA.raw	positive	Full scan	C. sinensis standard mixture	.raw
Qtof_P_E1_CSSM10uMB.raw	positive	Full scan	C. sinensis standard mixture	.raw
Qtof_P_E1_CSSM10uMC.raw	positive	Full scan	C. sinensis standard mixture	.raw
Qtof_P_E1_Wasteblank4.mzml	positive	Full scan	MeOH:H2O blank	.mzml
Qtof_P_E1_Wasteblank5.mzml	positive	Full scan	MeOH:H2O blank	.mzml
Qtof_P_E1_Wasteblank6.mzml	positive	Full scan	MeOH:H2O blank	.mzml
Qtof_P_E1_EXBLANKA.mzml	positive	Full scan	Extraction blank	.mzml
Qtof_P_E1_EXBLANKB.mzml	positive	Full scan	Extraction blank	.mzml
Qtof_P_E1_EXBLANKC.mzml	positive	Full scan	Extraction blank	.mzml
Qtof_P_E1_CS02A.mzml	positive	Full scan	Example sample	.mzml
Qtof_P_E1_CS02B.mzml	positive	Full scan	Example sample	.mzml
Qtof_P_E1_CS02C.mzml	positive	Full scan	Example sample	.mzml
Qtof_P_E1_CSSM10uMA.mzml	positive	Full scan	C. sinensis standard mixture	.mzml
Qtof_P_E1_CSSM10uMB.mzml	positive	Full scan	C. sinensis standard mixture	.mzml
Qtof_P_E1_CSSM10uMC.mzml	positive	Full scan	C. sinensis standard mixture	.mzml
Qtof_N_E1_Wasteblank4.raw	negative	Full scan	MeOH:H2O blank	.raw
Qtof_N_E1_Wasteblank5.raw	negative	Full scan	MeOH:H2O blank	.raw
Qtof_N_E1_Wasteblank6.raw	negative	Full scan	MeOH:H2O blank	.raw
Qtof_N_E1_EXBLANKA.raw	negative	Full scan	Extraction blank	.raw

Qtof_N_E1_EXBLANKB.raw	negative	Full scan	Extraction blank	.raw
Qtof_N_E1_EXBLANKC.raw	negative	Full scan	Extraction blank	.raw
Qtof_N_E1_CS02A.raw	negative	Full scan	Example sample	.raw
Qtof_N_E1_CS02B.raw	negative	Full scan	Example sample	.raw
Qtof_N_E1_CS02C.raw	negative	Full scan	Example sample	.raw
Qtof_N_E1_CSSM10uMA.raw	negative	Full scan	C. sinensis standard mixture	.raw
Qtof_N_E1_CSSM10uMB.raw	negative	Full scan	C. sinensis standard mixture	.raw
Qtof_N_E1_CSSM10uMC.raw	negative	Full scan	C. sinensis standard mixture	.raw
Qtof_N_E1_Wasteblank4.mzml	negative	Full scan	MeOH:H2O blank	.mzml
Qtof_N_E1_Wasteblank5.mzml	negative	Full scan	MeOH:H2O blank	.mzml
Qtof_N_E1_Wasteblank6.mzml	negative	Full scan	MeOH:H2O blank	.mzml
Qtof_N_E1_EXBLANKA.mzml	negative	Full scan	Extraction blank	.mzml
Qtof_N_E1_EXBLANKB.mzml	negative	Full scan	Extraction blank	.mzml
Qtof_N_E1_EXBLANKC.mzml	negative	Full scan	Extraction blank	.mzml
Qtof_N_E1_CS02A.mzml	negative	Full scan	Example sample	.mzml
Qtof_N_E1_CS02B.mzml	negative	Full scan	Example sample	.mzml
Qtof_N_E1_CS02C.mzml	negative	Full scan	Example sample	.mzml
Qtof_N_E1_CSSM10uMA.mzml	negative	Full scan	C. sinensis standard mixture	.mzml
Qtof_N_E1_CSSM10uMB.mzml	negative	Full scan	C. sinensis standard mixture	.mzml
Qtof_N_E1_CSSM10uMC.mzml	negative	Full scan	C. sinensis standard mixture	.mzml
Qtof_P_E2_Wasteblank4.raw	positive	DDA	MeOH:H2O blank	.raw
Qtof_P_E2_Wasteblank5.raw	positive	DDA	MeOH:H2O blank	.raw
Qtof_P_E2_Wasteblank6.raw	positive	DDA	MeOH:H2O blank	.raw
Qtof_P_E2_EXBLANKA.raw	positive	DDA	Extraction blank	.raw
Qtof_P_E2_EXBLANKB.raw	positive	DDA	Extraction blank	.raw
Qtof_P_E2_EXBLANKC.raw	positive	DDA	Extraction blank	.raw
Qtof_P_E2_CS02A.raw	positive	DDA	Example sample	.raw
Qtof_P_E2_CS02B.raw	positive	DDA	Example sample	.raw
Qtof_P_E2_CS02C.raw	positive	DDA	Example sample	.raw
Qtof_P_E2_CSSM10uMA.raw	positive	DDA	C. sinensis standard mixture	.raw
Qtof_P_E2_CSSM10uMB.raw	positive	DDA	C. sinensis standard mixture	.raw
Qtof_P_E2_CSSM10uMC.raw	positive	DDA	C. sinensis standard mixture	.raw
Qtof_P_E2_Wasteblank4.mzml	positive	DDA	MeOH:H2O blank	.mzml
Qtof_P_E2_Wasteblank5.mzml	positive	DDA	MeOH:H2O blank	.mzml
Qtof_P_E2_Wasteblank6.mzml	positive	DDA	MeOH:H2O blank	.mzml
Qtof_P_E2_EXBLANKA.mzml	positive	DDA	Extraction blank	.mzml
Qtof_P_E2_EXBLANKB.mzml	positive	DDA	Extraction blank	.mzml
Qtof_P_E2_EXBLANKC.mzml	positive	DDA	Extraction blank	.mzml
Qtof_P_E2_CS02A.mzml	positive	DDA	Example sample	.mzml
Qtof_P_E2_CS02B.mzml	positive	DDA	Example sample	.mzml
Qtof_P_E2_CS02C.mzml	positive	DDA	Example sample	.mzml
Qtof_P_E2_CSSM10uMA.mzml	positive	DDA	C. sinensis standard mixture	.mzml
Qtof_P_E2_CSSM10uMB.mzml	positive	DDA	C. sinensis standard mixture	.mzml
Qtof_P_E2_CSSM10uMC.mzml	positive	DDA	C. sinensis standard mixture	.mzml

Qtof_N_E2_Wasteblank4.raw	negative	DDA	MeOH:H2O blank	.raw
Qtof_N_E2_Wasteblank5.raw	negative	DDA	MeOH:H2O blank	.raw
Qtof_N_E2_Wasteblank6.raw	negative	DDA	MeOH:H2O blank	.raw
Qtof_N_E2_EXBLANKA.raw	negative	DDA	Extraction blank	.raw
Qtof_N_E2_EXBLANKB.raw	negative	DDA	Extraction blank	.raw
Qtof_N_E2_EXBLANKC.raw	negative	DDA	Extraction blank	.raw
Qtof_N_E2_CS02A.raw	negative	DDA	Example sample	.raw
Qtof_N_E2_CS02B.raw	negative	DDA	Example sample	.raw
Qtof_N_E2_CS02C.raw	negative	DDA	Example sample	.raw
Qtof_N_E2_CSSM10uMA.raw	negative	DDA	C. sinensis standard mixture	.raw
Qtof_N_E2_CSSM10uMB.raw	negative	DDA	C. sinensis standard mixture	.raw
Qtof_N_E2_CSSM10uMC.raw	negative	DDA	C. sinensis standard mixture	.raw
Qtof_N_E2_Wasteblank4.mzml	negative	DDA	MeOH:H2O blank	.mzml
Qtof_N_E2_Wasteblank5.mzml	negative	DDA	MeOH:H2O blank	.mzml
Qtof_N_E2_Wasteblank6.mzml	negative	DDA	MeOH:H2O blank	.mzml
Qtof_N_E2_EXBLANKA.mzml	negative	DDA	Extraction blank	.mzml
Qtof_N_E2_EXBLANKB.mzml	negative	DDA	Extraction blank	.mzml
Qtof_N_E2_EXBLANKC.mzml	negative	DDA	Extraction blank	.mzml
Qtof_N_E2_CS02A.mzml	negative	DDA	Example sample	.mzml
Qtof_N_E2_CS02B.mzml	negative	DDA	Example sample	.mzml
Qtof_N_E2_CS02C.mzml	negative	DDA	Example sample	.mzml
Qtof_N_E2_CSSM10uMA.mzml	negative	DDA	C. sinensis standard mixture	.mzml
Qtof_N_E2_CSSM10uMB.mzml	negative	DDA	C. sinensis standard mixture	.mzml
Qtof_N_E2_CSSM10uMC.mzml	negative	DDA	C. sinensis standard mixture	.mzml
Qtof_P_E3_Wasteblank4.raw	positive	DIA	MeOH:H2O blank	.raw
Qtof_P_E3_Wasteblank5.raw	positive	DIA	MeOH:H2O blank	.raw
Qtof_P_E3_Wasteblank6.raw	positive	DIA	MeOH:H2O blank	.raw
Qtof_P_E3_EXBLANKA.raw	positive	DIA	Extraction blank	.raw
Qtof_P_E3_EXBLANKB.raw	positive	DIA	Extraction blank	.raw
Qtof_P_E3_EXBLANKC.raw	positive	DIA	Extraction blank	.raw
Qtof_P_E3_CS02A.raw	positive	DIA	Example sample	.raw
Qtof_P_E3_CS02B.raw	positive	DIA	Example sample	.raw
Qtof_P_E3_CS02C.raw	positive	DIA	Example sample	.raw
Qtof_P_E3_CSSM10uMA.raw	positive	DIA	C. sinensis standard mixture	.raw
Qtof_P_E3_CSSM10uMB.raw	positive	DIA	C. sinensis standard mixture	.raw
Qtof_P_E3_CSSM10uMC.raw	positive	DIA	C. sinensis standard mixture	.raw
Qtof_P_E3_Wasteblank4.mzml	positive	DIA	MeOH:H2O blank	.mzml
Qtof_P_E3_Wasteblank5.mzml	positive	DIA	MeOH:H2O blank	.mzml
Qtof_P_E3_Wasteblank6.mzml	positive	DIA	MeOH:H2O blank	.mzml
Qtof_P_E3_EXBLANKA.mzml	positive	DIA	Extraction blank	.mzml
Qtof_P_E3_EXBLANKB.mzml	positive	DIA	Extraction blank	.mzml
Qtof_P_E3_EXBLANKC.mzml	positive	DIA	Extraction blank	.mzml
Qtof_P_E3_CS02A.mzml	positive	DIA	Example sample	.mzml
Qtof_P_E3_CS02B.mzml	positive	DIA	Example sample	.mzml
Qtof_P_E3_CS02C.mzml	positive	DIA	Example sample	.mzml

Qtof_P_E3_CSSM10uMA.mzml	positive	DIA	C. sinensis standard mixture	.mzml
Qtof_P_E3_CSSM10uMB.mzml	positive	DIA	C. sinensis standard mixture	.mzml
Qtof_P_E3_CSSM10uMC.mzml	positive	DIA	C. sinensis standard mixture	.mzml
Qtof_N_E3_Wasteblank4.raw	negative	DIA	MeOH:H2O blank	.raw
Qtof_N_E3_Wasteblank5.raw	negative	DIA	MeOH:H2O blank	.raw
Qtof_N_E3_Wasteblank6.raw	negative	DIA	MeOH:H2O blank	.raw
Qtof_N_E3_EXBLANKA.raw	negative	DIA	Extraction blank	.raw
Qtof_N_E3_EXBLANKB.raw	negative	DIA	Extraction blank	.raw
Qtof_N_E3_EXBLANKC.raw	negative	DIA	Extraction blank	.raw
Qtof_N_E3_CS02A.raw	negative	DIA	Example sample	.raw
Qtof_N_E3_CS02B.raw	negative	DIA	Example sample	.raw
Qtof_N_E3_CS02C.raw	negative	DIA	Example sample	.raw
Qtof_N_E3_CSSM10uMA.raw	negative	DIA	C. sinensis standard mixture	.raw
Qtof_N_E3_CSSM10uMB.raw	negative	DIA	C. sinensis standard mixture	.raw
Qtof_N_E3_CSSM10uMC.raw	negative	DIA	C. sinensis standard mixture	.raw
Qtof_N_E3_Wasteblank4.mzml	negative	DIA	MeOH:H2O blank	.mzml
Qtof_N_E3_Wasteblank5.mzml	negative	DIA	MeOH:H2O blank	.mzml
Qtof_N_E3_Wasteblank6.mzml	negative	DIA	MeOH:H2O blank	.mzml
Qtof_N_E3_EXBLANKA.mzml	negative	DIA	Extraction blank	.mzml
Qtof_N_E3_EXBLANKB.mzml	negative	DIA	Extraction blank	.mzml
Qtof_N_E3_EXBLANKC.mzml	negative	DIA	Extraction blank	.mzml
Qtof_N_E3_CS02A.mzml	negative	DIA	Example sample	.mzml
Qtof_N_E3_CS02B.mzml	negative	DIA	Example sample	.mzml
Qtof_N_E3_CS02C.mzml	negative	DIA	Example sample	.mzml
Qtof_N_E3_CSSM10uMA.mzml	negative	DIA	C. sinensis standard mixture	.mzml
Qtof_N_E3_CSSM10uMB.mzml	negative	DIA	C. sinensis standard mixture	.mzml
Qtof_N_E3_CSSM10uMC.mzml	negative	DIA	C. sinensis standard mixture	.mzml

*CS Orbi Example Dataset**

**For your own benchmarking only – do not submit analysis of these data to the group*

**naming structure**	_X	_XX	_samplename	.raw or .mzml
instrument	N = Negative mode	E1 = Full scan	Wasteblank = Methanol:H2O blank	
	P = Positive mode	E2 = DDA	EXBLANK = extraction blank	
		E3 = DIA	Alphanumeric code for plant sample	
			CS = Example dataset	
			WS = Evaluation dataset	
			CSSM10uM = mixture of 17 standards	

Full list of data files.

Orbi_P_E1_Wasteblank4.raw	positive	Full scan	MeOH:H2O blank	.raw
Orbi_P_E1_Wasteblank5.raw	positive	Full scan	MeOH:H2O blank	.raw
Orbi_P_E1_Wasteblank6.raw	positive	Full scan	MeOH:H2O blank	.raw
Orbi_P_E1_EXBLANKA.raw	positive	Full scan	Extraction blank	.raw
Orbi_P_E1_EXBLANKB.raw	positive	Full scan	Extraction blank	.raw
Orbi_P_E1_EXBLANKC.raw	positive	Full scan	Extraction blank	.raw
Orbi_P_E1_CS02A.raw	positive	Full scan	Example sample	.raw
Orbi_P_E1_CS02B.raw	positive	Full scan	Example sample	.raw
Orbi_P_E1_CS02C.raw	positive	Full scan	Example sample	.raw
Orbi_P_E1_CSSM10uMA.raw	positive	Full scan	C. sinensis standard mixture	.raw
Orbi_P_E1_CSSM10uMB.raw	positive	Full scan	C. sinensis standard mixture	.raw
Orbi_P_E1_CSSM10uMC.raw	positive	Full scan	C. sinensis standard mixture	.raw
Orbi_P_E1_Wasteblank4.mzml	positive	Full scan	MeOH:H2O blank	.mzml
Orbi_P_E1_Wasteblank5.mzml	positive	Full scan	MeOH:H2O blank	.mzml
Orbi_P_E1_Wasteblank6.mzml	positive	Full scan	MeOH:H2O blank	.mzml
Orbi_P_E1_EXBLANKA.mzml	positive	Full scan	Extraction blank	.mzml
Orbi_P_E1_EXBLANKB.mzml	positive	Full scan	Extraction blank	.mzml
Orbi_P_E1_EXBLANKC.mzml	positive	Full scan	Extraction blank	.mzml
Orbi_P_E1_CS02A.mzml	positive	Full scan	Example sample	.mzml
Orbi_P_E1_CS02B.mzml	positive	Full scan	Example sample	.mzml
Orbi_P_E1_CS02C.mzml	positive	Full scan	Example sample	.mzml
Orbi_P_E1_CSSM10uMA.raw	positive	Full scan	C. sinensis standard mixture	.mzml
Orbi_P_E1_CSSM10uMB.raw	positive	Full scan	C. sinensis standard mixture	.mzml
Orbi_P_E1_CSSM10uMC.raw	positive	Full scan	C. sinensis standard mixture	.mzml
Orbi_N_E1_Wasteblank4.raw	negative	Full scan	MeOH:H2O blank	.raw
Orbi_N_E1_Wasteblank5.raw	negative	Full scan	MeOH:H2O blank	.raw
Orbi_N_E1_Wasteblank6.raw	negative	Full scan	MeOH:H2O blank	.raw
Orbi_N_E1_EXBLANKA.raw	negative	Full scan	Extraction blank	.raw

Orbi_N_E1_EXBLANKB.raw	negative	Full scan	Extraction blank	.raw
Orbi_N_E1_EXBLANKC.raw	negative	Full scan	Extraction blank	.raw
Orbi_N_E1_CS02A.raw	negative	Full scan	Example sample	.raw
Orbi_N_E1_CS02B.raw	negative	Full scan	Example sample	.raw
Orbi_N_E1_CS02C.raw	negative	Full scan	Example sample	.raw
Orbi_N_E1_CSSM10uMA.raw	negative	Full scan	C. sinensis standard mixture	.raw
Orbi_N_E1_CSSM10uMB.raw	negative	Full scan	C. sinensis standard mixture	.raw
Orbi_N_E1_CSSM10uMC.raw	negative	Full scan	C. sinensis standard mixture	.raw
Orbi_N_E1_Wasteblank4.mzml	negative	Full scan	MeOH:H2O blank	.mzml
Orbi_N_E1_Wasteblank5.mzml	negative	Full scan	MeOH:H2O blank	.mzml
Orbi_N_E1_Wasteblank6.mzml	negative	Full scan	MeOH:H2O blank	.mzml
Orbi_N_E1_EXBLANKA.mzml	negative	Full scan	Extraction blank	.mzml
Orbi_N_E1_EXBLANKB.mzml	negative	Full scan	Extraction blank	.mzml
Orbi_N_E1_EXBLANKC.mzml	negative	Full scan	Extraction blank	.mzml
Orbi_N_E1_CS02A.mzml	negative	Full scan	Example sample	.mzml
Orbi_N_E1_CS02B.mzml	negative	Full scan	Example sample	.mzml
Orbi_N_E1_CS02C.mzml	negative	Full scan	Example sample	.mzml
Orbi_N_E1_CSSM10uMA.raw	negative	Full scan	C. sinensis standard mixture	.mzml
Orbi_N_E1_CSSM10uMB.raw	negative	Full scan	C. sinensis standard mixture	.mzml
Orbi_N_E1_CSSM10uMC.raw	negative	Full scan	C. sinensis standard mixture	.mzml
Orbi_P_E2_Wasteblank4.raw	positive	DDA	MeOH:H2O blank	.raw
Orbi_P_E2_Wasteblank5.raw	positive	DDA	MeOH:H2O blank	.raw
Orbi_P_E2_Wasteblank6.raw	positive	DDA	MeOH:H2O blank	.raw
Orbi_P_E2_EXBLANKA.raw	positive	DDA	Extraction blank	.raw
Orbi_P_E2_EXBLANKB.raw	positive	DDA	Extraction blank	.raw
Orbi_P_E2_EXBLANKC.raw	positive	DDA	Extraction blank	.raw
Orbi_P_E2_CS02A.raw	positive	DDA	Example sample	.raw
Orbi_P_E2_CS02B.raw	positive	DDA	Example sample	.raw
Orbi_P_E2_CS02C.raw	positive	DDA	Example sample	.raw
Orbi_P_E2_CSSM10uMA.raw	positive	DDA	C. sinensis standard mixture	.raw
Orbi_P_E2_CSSM10uMB.raw	positive	DDA	C. sinensis standard mixture	.raw
Orbi_P_E2_CSSM10uMC.raw	positive	DDA	C. sinensis standard mixture	.raw
Orbi_P_E2_Wasteblank4.mzml	positive	DDA	MeOH:H2O blank	.mzml
Orbi_P_E2_Wasteblank5.mzml	positive	DDA	MeOH:H2O blank	.mzml
Orbi_P_E2_Wasteblank6.mzml	positive	DDA	MeOH:H2O blank	.mzml
Orbi_P_E2_EXBLANKA.mzml	positive	DDA	Extraction blank	.mzml
Orbi_P_E2_EXBLANKB.mzml	positive	DDA	Extraction blank	.mzml
Orbi_P_E2_EXBLANKC.mzml	positive	DDA	Extraction blank	.mzml
Orbi_P_E2_CS02A.mzml	positive	DDA	Example sample	.mzml
Orbi_P_E2_CS02B.mzml	positive	DDA	Example sample	.mzml
Orbi_P_E2_CS02C.mzml	positive	DDA	Example sample	.mzml
Orbi_P_E2_CSSM10uMA.raw	positive	DDA	C. sinensis standard mixture	.mzml
Orbi_P_E2_CSSM10uMB.raw	positive	DDA	C. sinensis standard mixture	.mzml
Orbi_P_E2_CSSM10uMC.raw	positive	DDA	C. sinensis standard mixture	.mzml

Orbi_N_E2_Wasteblank4.raw	negative	DDA	MeOH:H2O blank	.raw
Orbi_N_E2_Wasteblank5.raw	negative	DDA	MeOH:H2O blank	.raw
Orbi_N_E2_Wasteblank6.raw	negative	DDA	MeOH:H2O blank	.raw
Orbi_N_E2_EXBLANKA.raw	negative	DDA	Extraction blank	.raw
Orbi_N_E2_EXBLANKB.raw	negative	DDA	Extraction blank	.raw
Orbi_N_E2_EXBLANKC.raw	negative	DDA	Extraction blank	.raw
Orbi_N_E2_CS02A.raw	negative	DDA	Example sample	.raw
Orbi_N_E2_CS02B.raw	negative	DDA	Example sample	.raw
Orbi_N_E2_CS02C.raw	negative	DDA	Example sample	.raw
Orbi_N_E2_CSSM10uMA.raw	negative	DDA	C. sinensis standard mixture	.raw
Orbi_N_E2_CSSM10uMB.raw	negative	DDA	C. sinensis standard mixture	.raw
Orbi_N_E2_CSSM10uMC.raw	negative	DDA	C. sinensis standard mixture	.raw
Orbi_N_E2_Wasteblank4.mzml	negative	DDA	MeOH:H2O blank	.mzml
Orbi_N_E2_Wasteblank5.mzml	negative	DDA	MeOH:H2O blank	.mzml
Orbi_N_E2_Wasteblank6.mzml	negative	DDA	MeOH:H2O blank	.mzml
Orbi_N_E2_EXBLANKA.mzml	negative	DDA	Extraction blank	.mzml
Orbi_N_E2_EXBLANKB.mzml	negative	DDA	Extraction blank	.mzml
Orbi_N_E2_EXBLANKC.mzml	negative	DDA	Extraction blank	.mzml
Orbi_N_E2_CS02A.mzml	negative	DDA	Example sample	.mzml
Orbi_N_E2_CS02B.mzml	negative	DDA	Example sample	.mzml
Orbi_N_E2_CS02C.mzml	negative	DDA	Example sample	.mzml
Orbi_N_E2_CSSM10uMA.raw	negative	DDA	C. sinensis standard mixture	.mzml
Orbi_N_E2_CSSM10uMB.raw	negative	DDA	C. sinensis standard mixture	.mzml
Orbi_N_E2_CSSM10uMC.raw	negative	DDA	C. sinensis standard mixture	.mzml
Orbi_P_E3_Wasteblank4.raw	positive	DIA	MeOH:H2O blank	.raw
Orbi_P_E3_Wasteblank5.raw	positive	DIA	MeOH:H2O blank	.raw
Orbi_P_E3_Wasteblank6.raw	positive	DIA	MeOH:H2O blank	.raw
Orbi_P_E3_EXBLANKA.raw	positive	DIA	Extraction blank	.raw
Orbi_P_E3_EXBLANKB.raw	positive	DIA	Extraction blank	.raw
Orbi_P_E3_EXBLANKC.raw	positive	DIA	Extraction blank	.raw
Orbi_P_E3_CS02A.raw	positive	DIA	Example sample	.raw
Orbi_P_E3_CS02B.raw	positive	DIA	Example sample	.raw
Orbi_P_E3_CS02C.raw	positive	DIA	Example sample	.raw
Orbi_P_E3_CSSM10uMA.raw	positive	DIA	C. sinensis standard mixture	.raw
Orbi_P_E3_CSSM10uMB.raw	positive	DIA	C. sinensis standard mixture	.raw
Orbi_P_E3_CSSM10uMC.raw	positive	DIA	C. sinensis standard mixture	.raw
Orbi_P_E3_Wasteblank4.mzml	positive	DIA	MeOH:H2O blank	.mzml
Orbi_P_E3_Wasteblank5.mzml	positive	DIA	MeOH:H2O blank	.mzml
Orbi_P_E3_Wasteblank6.mzml	positive	DIA	MeOH:H2O blank	.mzml
Orbi_P_E3_EXBLANKA.mzml	positive	DIA	Extraction blank	.mzml
Orbi_P_E3_EXBLANKB.mzml	positive	DIA	Extraction blank	.mzml
Orbi_P_E3_EXBLANKC.mzml	positive	DIA	Extraction blank	.mzml
Orbi_P_E3_CS02A.mzml	positive	DIA	Example sample	.mzml
Orbi_P_E3_CS02B.mzml	positive	DIA	Example sample	.mzml
Orbi_P_E3_CS02C.mzml	positive	DIA	Example sample	.mzml

Orbi_P_E3_CSSM10uMA.raw	positive	DIA	C. sinensis standard mixture	.mzml
Orbi_P_E3_CSSM10uMB.raw	positive	DIA	*C. sinensis* standard mixture	.mzml
Orbi_P_E3_CSSM10uMC.raw	positive	DIA	*C. sinensis* standard mixture	.mzml
Orbi_N_E3_Wasteblank4.raw	negative	DIA	MeOH:H2O blank	.raw
Orbi_N_E3_Wasteblank5.raw	negative	DIA	MeOH:H2O blank	.raw
Orbi_N_E3_Wasteblank6.raw	negative	DIA	MeOH:H2O blank	.raw
Orbi_N_E3_EXBLANKA.raw	negative	DIA	Extraction blank	.raw
Orbi_N_E3_EXBLANKB.raw	negative	DIA	Extraction blank	.raw
Orbi_N_E3_EXBLANKC.raw	negative	DIA	Extraction blank	.raw
Orbi_N_E3_CS02A.raw	negative	DIA	Example sample	.raw
Orbi_N_E3_CS02B.raw	negative	DIA	Example sample	.raw
Orbi_N_E3_CS02C.raw	negative	DIA	Example sample	.raw
Orbi_N_E3_CSSM10uMA.raw	negative	DIA	C. sinensis standard mixture	.raw
Orbi_N_E3_CSSM10uMB.raw	negative	DIA	C. sinensis standard mixture	.raw
Orbi_N_E3_CSSM10uMC.raw	negative	DIA	C. sinensis standard mixture	.raw
Orbi_N_E3_Wasteblank4.mzml	negative	DIA	MeOH:H2O blank	.mzml
Orbi_N_E3_Wasteblank5.mzml	negative	DIA	MeOH:H2O blank	.mzml
Orbi_N_E3_Wasteblank6.mzml	negative	DIA	MeOH:H2O blank	.mzml
Orbi_N_E3_EXBLANKA.mzml	negative	DIA	Extraction blank	.mzml
Orbi_N_E3_EXBLANKB.mzml	negative	DIA	Extraction blank	.mzml
Orbi_N_E3_EXBLANKC.mzml	negative	DIA	Extraction blank	.mzml
Orbi_N_E3_CS02A.mzml	negative	DIA	Example sample	.mzml
Orbi_N_E3_CS02B.mzml	negative	DIA	Example sample	.mzml
Orbi_N_E3_CS02C.mzml	negative	DIA	Example sample	.mzml
Orbi_N_E3_CSSM10uMA.raw	negative	DIA	C. sinensis standard mixture	.mzml
Orbi_N_E3_CSSM10uMB.raw	negative	DIA	C. sinensis standard mixture	.mzml
Orbi_N_E3_CSSM10uMC.raw	negative	DIA	C. sinensis standard mixture	.mzml
